# Investigating the Cytotoxicity of Ru(II) Polypyridyl Complexes by Changing the Electronic Structure of Salicylaldehyde Ligands

**DOI:** 10.1021/acs.inorgchem.3c03414

**Published:** 2023-12-29

**Authors:** Maryam Taghizadeh Shool, Hadi Amiri Rudbari, José V. Cuevas-Vicario, Andrea Rodríguez-Rubio, Claudio Stagno, Nunzio Iraci, Thomas Efferth, Ejlal A. Omer, Tanja Schirmeister, Olivier Blacque, Nakisa Moini, Esmail Sheibani, Nicola Micale

**Affiliations:** † Department of Chemistry, University of Isfahan, 81746-73441 Isfahan, Iran; ‡ Departamento de Química, Facultad de Ciencias, Universidad de Burgos, Plaza Misael Bañuelos s/n, 09001 Burgos, Spain; § Department of Chemical, Biological, Pharmaceutical and Environmental Sciences, University of Messina, Viale Ferdinando Stagno D’Alcontres 31, I-98166 Messina, Italy; ∥ Department of Pharmaceutical Biology, Institute of Pharmaceutical and Biomedical Sciences, Johannes Gutenberg University, Staudinger Weg 5, 55128 Mainz, Germany; ⊥ Department of Medicinal Chemistry, Institute of Pharmaceutical and Biomedical Sciences, Johannes Gutenberg University, Staudinger Weg 5, 55128 Mainz, Germany; # Department of Chemistry, University of Zurich, Winterthurerstrasse 190, CH-8057 Zurich, Switzerland; ¶ Department of Chemistry, Faculty Chemistry, Alzahra University, Vanak, P.O. Box 1993891176, 1993891176 Tehran, Iran

## Abstract

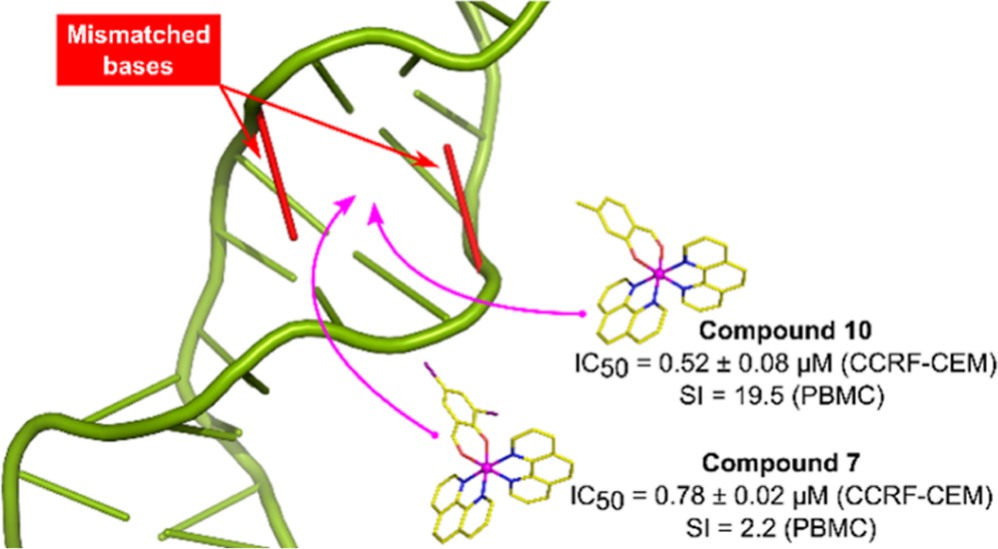

A novel class of Ru(II)-based polypyridyl complexes with an auxiliary salicylaldehyde ligand [Ru(phen)_2_(X-Sal)]BF_4_ {X: H (**1**), 5-Cl (**2**), 5-Br (**3**), 3,5-Cl_2_ (**4**), 3,5-Br_2_ (**5**), 3-Br,5-Cl (**6**), 3,5-I_2_ (**7**), 5-NO_2_ (**8**), 5-Me (**9**), 4-Me (**10**), 4-OMe (**11**), and 4-DEA (**12**), has been synthesized and characterized by elemental analysis, FT-IR, and ^1^H/^13^C NMR spectroscopy. The molecular structure of **4**, **6**, **9**, **10**, and **11** was determined by single-crystal X-ray diffraction analysis which revealed structural similarities. DFT and TD-DFT calculations showed that they also possess similar electronic structures. Absorption/emission spectra were recorded for **2**, **3**, **10**, and **11**. All Ru-complexes, unlike the pure ligands and the complex lacking the salicylaldehyde component, displayed outstanding antiproliferative activity in the screening test (10 μM) against CCRF-CEM leukemia cells underlining the crucial role of the presence of the auxiliary ligand for the biological activity. The two most active derivatives, namely **7** and **10**, were selected for continuous assays showing IC_50_ values in the submicromolar and micromolar range against drug-sensitive CCRF-CEM and multidrug-resistant CEM/ADR5000 leukemia cells, respectively. These two compounds were investigated in silico for their potential binding to duplex DNA well-matched and mismatched base pairs, since they showed remarkable selectivity indexes (2.2 and 19.5 respectively) on PBMC cells.

## Abstract 2

A panel of Ru(II)-based complexes with general formula [Ru(phen)_2_(X-Sal)]BF_4_ and distorted octahedral geometry was synthesized and characterized (elemental analysis and spectroscopic methods). All Ru-complexes, unlike pure ligands and complex lacking the salicylaldehyde auxiliary component, displayed outstanding antiproliferative activity against CCRF-CEM leukemia cells. The top two compounds showed remarkable IC_50_ values (submicromolar on CCRF-CEM) and selectivity indexes (up to 19.5 on PBMC). In silico studies indicated they bind preferentially to the anticancer target duplex DNA mismatched base pairs.

## Introduction

Cancer still represents a leading cause of death globally. According to the WHO reports, it accounted for nearly 10 million deaths in the year 2020, or nearly one in six deaths.^1^ However, if diagnosed and treated promptly, many types of cancer can be cured effectively.^2^ The most common methods of cancer treatment include surgery, radiotherapy, chemotherapy, hormonal treatments, and targeted (small-molecule drugs or monoclonal antibodies) and biological therapies (which eventually affect the immune system). In this context, metal-based compounds have noticeable potential as chemotherapeutic agents,^3,4^ in particular platinum-based drugs (cisplatin and its best-known derivatives carboplatin and oxaliplatin) which still represent the most used metal-based chemotherapeutics for the treatment of various tumors. Although these drugs are very effective in killing cancer cells, they bring about many toxic side-effects and drug resistance phenomena,^5−7^ factors that have directed the pharmaceutical industry toward the search for alternative nonplatinum-based metal compounds as cytostatic agents.^8,9^

**Figure 1:**
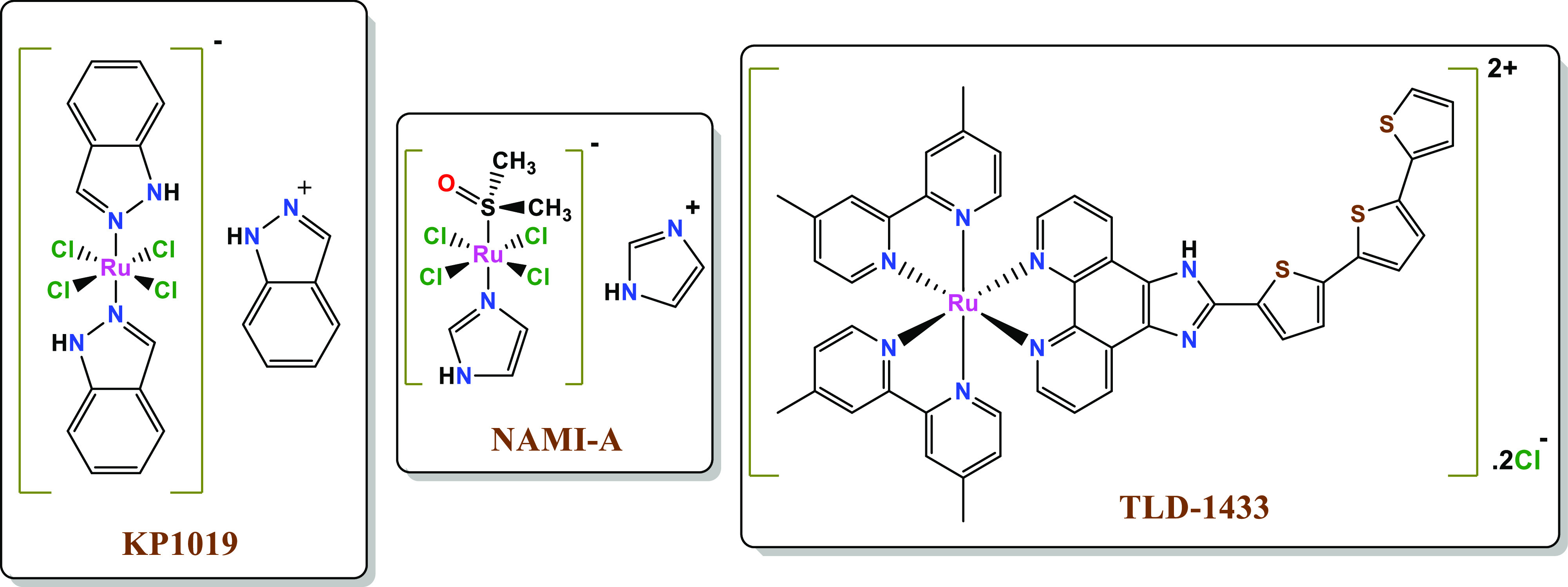
Chemical structure of **KP1019**, **NAMI-A**, and **TLD-1433**.

In this regard, ruthenium-based complexes are considered as reasonable candidates for anticancer drug design as they have several advantages over platinum-based drugs including: (1) several accessible and stable oxidation states under physiological conditions; (2) iron-mimicking ability in binding specific proteins which results in up-regulation of transferrin receptors on the cell surface and eventually in higher accumulation of ruthenium inside tumor cells compared to healthy cells; (3) various activation mechanisms combined with high biological activity; and (4) slow ligand exchange in vivo.^10−12^ As of yet, a number of ruthenium-based complexes, such as ([RuCl_4_(DMSO)(Im)]ImH; Im = imidazole) and (*trans*-[RuCl_4_(Ind)_2_]IndH; Ind = indole) (known as **NAMI-A** and **KP1019**, respectively; Figure 1), have shown promising anticancer activity and completed phase I and II clinical studies, which spurred efforts to develop new Ru-based compounds for the treatment of cancer.^13−15^

The oldest Ru(II)-based complexes endowed with polypyridyl ligands and whose biological activity has been investigated, namely [Ru(bpy)_3_](ClO_4_)_2_ and [Ru(phen)_3_](ClO_4_)_2_, date back to the 1950s.^16^ Since then, polypyridyl ligands with multiple covalently bonded pyridine groups have been extensively used in medicinal inorganic chemistry due to their unique photochemical, physicochemical, and biological properties.^17^ The most important of the Ru(II)-based polypyridyl complexes developed so far is considered the photosensitizer **TLD-1433** which has completed phase I and II clinical trials for photodynamic therapy treatment of the nonmuscle invasive bladder cancer (Figure 1).^18,19^

**Figure 2:**
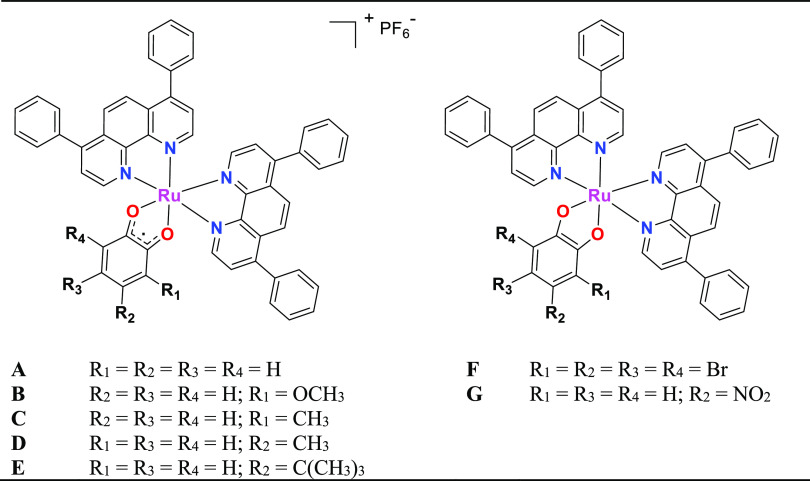
Chemical structure of the complexes **[Ru(DIP)**_**2**_**(sq)](PF**_**6**_**)** developed by Notaro et al.^20^

On the basis of previous studies, it has been determined that the chemical/electronic characteristics and position of the substituents in the structure of the ligand play a crucial role in the cytotoxic behavior of this type of complexes. To get insights into their structure–activity relationships, Notaro et al. have recently studied Ru(II) polypyridyl complexes with substituted catecholate ligands bearing either electron-donating (EDG) or electron-withdrawing groups (EWG), namely **[Ru(DIP)**_**2**_**(sq)](PF**_**6**_**)** (Figure 2).^20^ As indicated in this work, the difference in electron density on the catecholate ligand induces a variation in its oxidation state when coordinating with the metal center. The variation of the oxidation state of the ligand affects the physicochemical properties and biological activity of the resulting complexes. The cytotoxicity data revealed that the complexes with ligands bearing EDG show much higher bioactivity as compared to complexes with ligands bearing EWG as a substitution pattern. The complex with—OMe substitution (i.e., **B**; Figure 2) turned out to be the most promising compound of this series.^20^

In 2020, we have successfully synthesized a set of copper(II)-based complexes with general formula Cu(diimine)(x-Sal)(NO_3_) and investigated the effects of various halogen atoms on the diimine ligand and their impact on the antiproliferative activity against two different cancer cell lines.^21^ The obtained results highlighted that the bpy derivatives are valid candidates for further in vitro and in vivo studies. We also synthesized a new set of chiral Ru(II) polypyridyl complexes, namely Δ/Λ-[Ru(bpy)_2_(X,Y-Sal)]BF_4_, where X,Y-Sal is halogenated salicylaldehyde with chloride and/or bromide substitutions in 3 and 5 positions.^22^ We also found that the type, number, and position of the halogen substituents are important factors in determining the cytotoxicity of these compounds.

**Scheme 1:**
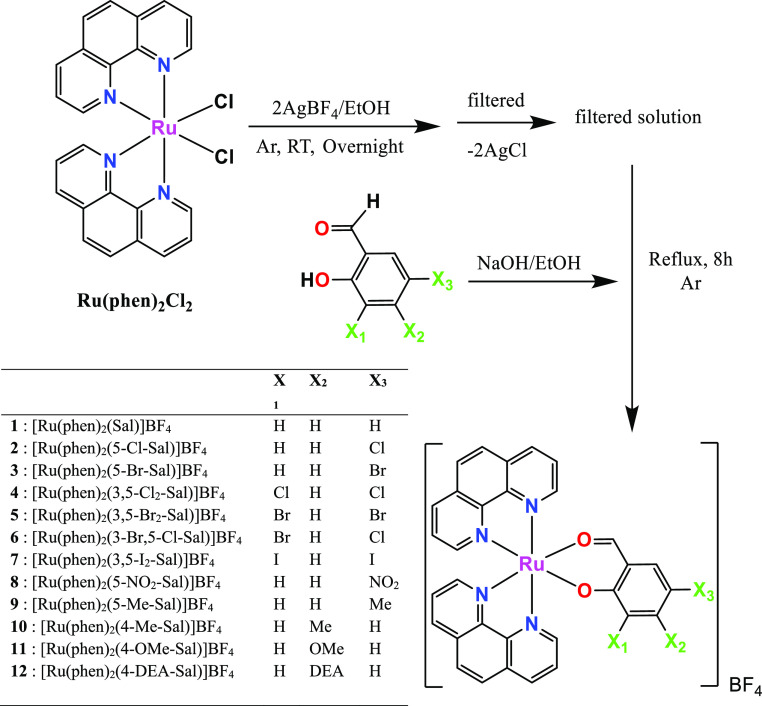
Synthetic Route and Chemical Structure of the Newly Synthesized Ru(II) Complexes [Ru(phen)_2_(X-Sal)]BF_4_ (**1**–**12**)

Herein, we report synthesis, structural characterization, photophysical properties, antiproliferative activity, SAR analysis of the ligand substitution pattern, and computational study on the expected biomolecular target (i.e., DNA) of a new set of 12 Ru(II) polypyridyl complexes obtained from the starting complex **Ru(phen)**_**2**_**Cl**_**2**_ and substituted salicylaldehyde ligands (Scheme 1).

## Experimental Section

### Chemicals and Instrumentation

Ruthenium(III)-chloride hydrate, 1,10-phenanthroline, sodium hydroxide, silver tetrafluoroborate, salicylaldehyde, 5-chlorosalicylaldehyde, 3,5-chlorosalicylaldehyde, 5-bromosalicylaldehyde, 3,5-bromosalicylaldehyde, 3-bromo-5-chlorosalicylaldehyde, 3,5-diiodosalicylaldehyde, 4-(diethylamino)salicylaldehyde, 5-nitrosalicylaldehyde, 4-methoxysalicylaldehyde, 5-methoxysalicylaldehyde, 4-methylsalicylaldehyde, 5-methylsalicylaldehyde were obtained from Sigma-Aldrich and used without any further purification. The solvents obtained from the same commercial source, however, were subjected to a distillation process before being used for the synthesis of the complexes. *cis*-**Ru(phen)**_**2**_**Cl**_**2**_ was prepared according to literature procedures.^23^ FT-IR spectra were recorded using KBr pellets on a JASCO FT/IR-6300 spectrometer (4000–400 cm^–1^). Elemental analyses were carried out using both the LECO’s CHNS-932 and PerkinElmer 7300 DV elemental analyzer. NMR spectra were recorded in quartz NMR tube by means of Bruker high resolution Avance NEO 4500 (500 MHz) and Bruker Avance III HD 400 MHz spectrometer and using DMSO-*d*_6_ as a solvent at 295 K.

### Synthesis of the Complexes

All [Ru(phen)_2_L]BF_4_ complexes were obtained by a general synthetic method. Briefly, the starting complex **Ru(phen)**_**2**_**Cl**_**2**_ (1 mmol) and AgBF_4_ (2 mmol) were dissolved in ethanol. The resulting reaction solution was then stirred overnight at room temperature and under argon atmosphere. After that, the reaction mixture was filtered to remove the AgCl that had formed in the meantime and to the resulting orange-red solution were added in sequence an ethanol solution of substituted salicylaldehyde (**HL**) (1 mmol) and NaOH (1 mmol). The new reaction mixture was stirred at reflux and under argon atmosphere for ∼8 h. The solvent was then removed under vacuum providing a solid which was dissolved in the minimum amount of chloroform and precipitated with *n*-hexane.

#### [Ru(phen)_2_(Sal)]BF_4_ (**1**)

Yield 89%. Anal. Calcd for C_31_H_21_BF_4_N_4_O_2_Ru: C, 55.62; H, 3.16; N, 8.37. Found: C, 55.65; H, 3.15; N, 8.39. IR (KBr, cm^–1^): 1605 (s, C=O), 1057 (s, B–F). ^1^H NMR (400 MHz, DMSO-*d*_6_): δ ppm 9.19 (dd, 1H, H^6^ or H^21^), 9.17 (s, 1H, H^1^), 9.10 (dd, 1H, H^6^ or H^21^), 8.87 (dd, 1H, H^8^ or H^19^), 8.83 (dd, 1H, H^8^ or H^19^), 8.49 (m, 2H, H^11^ and H^16^), 8.38 (d, 2H, H^9^ and H^18^), 8.29 (m, 2H, H^10^ and H^17^), 8.18 (dd, 1H, H^7^ or H^20^ or H^13^ or H^14^), 8.11 (m, 2H, H^7^ or H^20^ or H^13^ or H^14^), 8.04 (d, 1H, H^7^ or H^20^ or H^13^ or H^14^), 7.52 (td, 2H, H^12^, H^15^), 7.42 (dd, 1H, H^2^), 7.20 (m, 1H, H^3^ or H^4^), 6.52 (d, 1H, H^5^), 6.44 (t, 1H, H^3^ or H^4^). ^13^C NMR (100 MHz, DMSO-*d*_6_): δ ppm 190.1 (C^1^), 170.3 (C^23^), 154.7 (C^13^ or C^14^), 154.3 (C^13^ or C^14^), 151.1 (C^6^ or C^21^), 151.0 (C^6^ or C^21^), 149.9 (C^27^ or C^28^), 149.3 (C^27^ or C^28^), 148.3 (C^24^ or C^31^), 148.1 (C^24^or C^31^), 137.3 (C^2^ or C^5^), 136.1 (C^2^ or C^5^), 136.0 (C^8^ or C^19^), 135.6 (C^8^ or C^19^), 134.3 (C^11^ or C^16^), 134.1 (C^11^ or C^16^), 130.0, 129.9, 129.9, and 129.7 (C^25^, C^26^, C^29^ and C^30^), 127.7, 127.6 (C^9^, C^10^, C^17^ and C^18^), 125.8 (C^7^ or C^20^), 125.5 (C^7^ or C^20^), 124.8 (C^12^ or C^15^), 124.7 (C^12^ or C^15^), 124.2(C^22^), 122.2 (C^3^ or C^4^), 114.2 (C^3^ or C^4^).

#### [Ru(phen)_2_(5-Cl-Sal)]BF_4_ (**2**)

Yield 84%. Anal. Calcd for C_31_H_20_BClF_4_N_4_O_2_Ru: C, 52.90; H, 2.86; N, 7.96. Found: C, 52.94; H, 2.89; N, 7.97. IR (KBr, cm^–1^): 1580 (s, C=O), 1058 (s, B–F). ^1^H NMR (500 MHz, DMSO-*d*_6_): δ ppm 9.21–9.17 (m, 2H, H^1^ and H^5^), 9.08 (dd, *J* = 5.1, 1.3 Hz, 1H, H^20^), 8.88 (dd, *J* = 8.3, 1.3 Hz, 1H, H^18^), 8.83 (dd, *J* = 8.2, 1.3 Hz, 1H, H^7^), 8.50 (dd, *J* = 8.2, 1.2 Hz, 1H, H^10^ or H^15^), 8.47 (dd, *J* = 8.2, 1.2 Hz, 1H, H^10^ or H^15^), 8.38 (m, 1.8 Hz, 2H, H^8^ and H^17^), 8.29 (d, *J* = 6.3 Hz, 1H, H^9^ or H^16^), 8.27 (d, *J* = 6.2 Hz, 1H, H^9^ or H^16^), 8.18 (dd, *J* = 8.3, 5.1 Hz, 1H, H^19^), 8.11 (dd, *J* = 8.2, 5.2 Hz, 1H, H^6^), 8.08 (dd, *J* = 5.4, 1.2 Hz, 1H, H^12^ or H^13^), 8.02 (dd, *J* = 5.3, 1.2 Hz, 1H, H^12^ or H^13^), 7.55–7.48 (m, 3H, H^2^ and H^11^ and H^14^), 7.17 (dd, *J* = 9.4, 2.9 Hz, 1H, H^4^), 6.53 (d, *J* = 9.4 Hz, 1H, H^3^). ^13^C NMR (126 MHz, DMSO-*d*_6_): δ ppm 189.7 (C^1^), 168.9 (C^23^), 154.8 (C^12^ or C^13^), 154.4 (C^12^ or C^13^), 151.3 (C^5^ or C^20^), 151.1 (C^5^ or C^20^), 149.8 (C^27^ or C^28^), 149.3 (C^27^ or C^28^), 148.3 (C^24^ or C^31^), 148.1 (C^24^ or C^31^), 136.3 (C^7^ or C^18^), 135. 9 (C^7^ or C^18^), 135.5 (C^4^), 134.8 (C^2^), 134.5 (C^10^ or C^15^), 134.3 (C^10^ or C^15^), 130.1, 130.0, 129.9, and 129.7 (C^25^, C^26^, C^29^ and C^30^), 127.7 and 127.6 (C^8^, C^9^, C^16^ and C^17^), 126. 8(C^3^), 125.9 (C^6^ or C^19^), 125.6 (C^6^ or C^19^), 124.9 (C^11^ or C^14^), 124.7 (C^11^ or C^14^), 122.8 (C^21^), 117.1 (C^22^).

#### [Ru(phen)_2_(5-Br-Sal)]BF_4_ (**3**)

Yield 81%. Anal. Calcd for C_31_H_20_BBrF_4_N_4_O_2_Ru: C, 49.76; H, 2.69; N, 7.49. Found: C, 49.78; H, 2.72; N, 7.53.IR (KBr, cm^–1^): 1648 (s, C=O), 1060 (s, B–F). ^1^H NMR (500 MHz, DMSO-*d*_6_): δ ppm 9.18 (m, 2H, H^1^ and H^5^), 9.08 (dd, *J* = 5.1, 1.3 Hz, 1H, H^20^), 8.88 (dd, *J* = 8.3, 1.3 Hz, 1H, H^18^), 8.83 (dd, *J* = 8.2, 1.2 Hz, 1H, H^7^), 8.50 (dd, *J* = 8.2, 1.1 Hz, 1H, H^10^ or H^15^), 8.47 (dd, *J* = 8.2, 1.1 Hz, 1H, H^10^ or H^15^), 8.38 (m, 2H, H^8^ and H^17^), 8.27 (m, 2H, H^9^ and H^16^), 8.18 (dd, *J* = 8.3, 5.1 Hz, 1H, H^19^), 8.11 (dd, *J* = 8.2, 5.2 Hz, 1H, H^6^), 8.08 (dd, *J* = 5.3, 1.1 Hz, 1H, H^12^ or H^13^), 8.02 (dd, *J* = 5.4, 1.1 Hz, 1H, H^12^ or H^13^), 7.65 (d, *J* = 2.8 Hz, 1H, H^2^), 7.51 (m, 2H, H^11^ and H^14^), 7.24 (dd, *J* = 9.4, 2.8 Hz, 1H, H^4^), 6.47 (d, *J* = 9.4 Hz, 1H, H^3^). ^13^C NMR (126 MHz, DMSO-*d*_6_): δ ppm 189.79 (C^1^), 169.1(C^23^), 154.8 (C^12^ or C^13^), 154.4 (C^12^ or C^13^), 151.3 (C^5^ or C^20^), 151.1 (C^5^ or C^20^), 149.8 (C^27^ or C^28^), 149.3 (C^27^ or C^28^), 148.3 (C^24^ or C^31^), 148.1 (C^24^ or C^31^) 138.1 (C^2^ or C^4^), 137.9 (C^2^ or C^4^), 136.4 (C^7^ or C^18^), 135.9 (C^7^ or C^18^), 134.5 (C^10^ or C^15^), 134.3 (C^10^ or C^15^), 130.1, 130.0, 129. 9 and 129.8 (C^25^, C^26^, C^29^ and C^30^), 127.7 and 127.6 (C^8^, C^9^, C^16^ and C^17^), 127.1 (C^3^), 125.9 (C^6^ or C^19^), 125.6 (C^6^ or C^19^), 124.9 (C^11^ or C^14^), 124.7 (C^11^ or C^14^), 123.8 (C^21^), 104.1 (C^22^).

#### [Ru(phen)_2_(3,5-Cl_2_-Sal)]BF_4_ (**4**)

Yield 85%. Anal. Calcd for C_31_H_19_BCl_2_F_4_N_4_O_2_Ru: C, 50.43; H, 2.59; N, 7.59. Found: C, 50.46; H, 2.61; N, 7.60. IR (KBr, cm^–1^): 1592 (s, C=O), 1061(s, B–F). ^1^H NMR (400 MHz, DMSO-*d*_6_): δ ppm 9.33 (s, 1H, H^1^), 9.22 (dd, 1H, H^4^ or H^19^), 9.00 (dd, 1H, H^4^ or H^19^), 8.91 (dd, 1H, H^17^ or H^6^), 8.86 (dd, 1H, H^17^ or H^6^), 8.51 (t, 2H, H^5^ and H^18^), 8.39 (m, 2H, H^14^ and H^9^), 8.29 (m, 2H, H^7^ and H^16^ or H^8^ and H^15^ or H^11^ and H^12^), 8.21 (dd, 1H, H^7^ and H^16^ or H^8^ and H^15^ or H^11^ and H^12^), 8.12 (m, 2H, H^7^ and H^16^ or H^8^ and H^15^ or H^11^ and H^12^), 8.06 (dd, 1H, H^7^ and H^16^ or H^8^ and H^15^ or H^11^ and H^1^), 7.58 (dd, 2H, H^2^ and H^3^), 7.54 (dd, 2H, H^10^ and H^13^). ^13^C NMR (100 MHz, DMSO-*d*_6_): δ ppm 190.9 (C^1^), 162.3 (C^23^), 155.0 (C^11^ or C^12^), 154.4 (C^11^ or C^12^), 151.3 (C^4^ or C^19^), 150.8 (C^4^or C^19^), 149.8 (C^27^ or C^28^), 149.2 (C^27^ or C^28^), 148.2 (C^24^ or C^31^), 148.0 (C^24^ or C^31^), 136.5 (C^6^or C^17^), 136.1 (C^6^or C^17^), 136.6 (C^2^ or C^3^), 134.5 (C^2^ or C^3^), 134.3 (C^9^ or C^14^), 133. 8 (C^9^ or C^14^), 130.0, 130.0, 129.9, and 129.6 (C^25^, C^26^, C^29^ and C^30^), 129.1 (C^21^ or C^22^), 127.7 and 126.6 (C^7^, C^8^, C^15^ and C^16^), 126.1 (C^5^or C^18^), 125.6 (C^5^or C^18^), 124.9 (C^10^ or C^13^), 124. 7 (C^10^ or C^13^), 123.0 (C^20^), 116.0 (C^21^ or C^22^).

#### [Ru(phen)_2_(3,5-Br_2_-Sal)]BF_4_ (**5**)

Yield 92%. Anal. Calcd for C_31_H_19_BBr_2_F_4_N_4_O_2_Ru: C, 45.01; H, 2.32; N, 6.77. Found: C, 45.04; H, 2.30; N, 6.79. IR (KBr, cm^–1^): 15.86 (s, C=O), 1059 (s, B–F). ^1^H NMR (400 MHz, DMSO-*d*_6_): δ ppm 9.33 (s, 1H, H^1^), 9.22 (dd, 1H, H^4^ or H^19^), 8.98 (dd, 1H, H^4^ or H^19^), 8.91 (dd, 1H, H^17^ or H^6^), 8.85 (dd, 1H, H^17^ or H^6^), 8.50 (m, 2H, H^9^ and H^14^), 8.39 (t, 2H, H^18^ and H^5^), 8.29 (m, 2H, H^7^ and H^16^ or H^8^ and H^15^ or H^11^ and H^12^), 8.23 (dd, 1H, H^7^ and H^16^ or H^8^ and H^15^ or H^11^ and H^12^), 8.13 (m, 2H, H^7^ and H^16^ or H^8^ and H^15^ or H^11^ and H^12^), 8.07 (dd, 1H, H^7^ and H^16^ or H^8^ and H^15^ or H^11^ and H^1^), 7.76 (dd, 2H, H^2^ and H^3^), 7.54 (dd, 2H, H^10^ and H^13^). ^13^C NMR (100 MHz, DMSO-*d*_6_): δ ppm 191.0 (C^1^), 162.9 (C^23^), 155.0 (C^11^ or C^12^), 154.4 (C^11^ or C^12^), 151.3 (C^4^or C^19^), 150.8 (C^4^or C^19^), 149.8 (C^27^ or C^28^), 149.2 (C^27^ or C^28^), 148.2 (C^24^ or C^31^), 148.0 (C^24^ or C^31^), 139.0 (C^2^ or C^3^), 138.3 (C^2^ or C^3^), 136.5 1 (C^6^or C^17^), 136.1 1 (C^6^or C^17^), 134.6 (C^9^ or C^14^), 134.5 (C^9^ or C^14^), 130.0, 129.9, and 129.6 (C^25^, C^26^, C^29^ and C^30^), 127.7, 127.6, and 127.6 (C^7^, C^8^, C^15^ and C^16^), 126.1 (C^5^or C^18^), 125.6 (C^5^or C^18^), 124.9 (C^10^ or C^13^), 124.6 (C^10^ or C^13^), 123.5 (C^20^), 120.6 (C^21^ or C^22^), 103.2 (C^21^ or C^22^).

#### [Ru(phen)_2_(3-Br,5Cl-Sal)]BF_4_ (**6**)

Yield 80%. Anal. Calcd for C_31_H_19_BBrClF_4_N_4_O_2_Ru: C, 47.57; H, 2.45; N, 8.05. Found: C, 47.60; H,2.46; N, 8.08. IR (KBr, cm^–1^): 1586(s, C=O), 1061(s, B–F). ^1^H NMR (400 MHz, DMSO-*d*_6_): δ ppm 9.33 (s, 1H, H^1^), 9.21 (dd, 1H, H^4^ or H^19^), 8.98 (dd, 1H, H^4^ or H^19^), 8.90 (dd, 1H, H^17^ or H^6^), 8.85 (dd, 1H, H^17^ or H^6^), 8.50 (m, 2H, H^9^ and H^14^), 8.39 (t, 2H, H^18^ and H^5^), 8.30 (m, 2H, H^7^ and H^16^ or H^8^ and H^15^ or H^11^ and H^12^), 8.23 (dd, 1H, H^7^ and H^16^ or H^8^ and H^15^ or H^11^ and H^12^), 8.13 (m, 2H, H^7^ and H^16^ or H^8^ and H^15^ or H^11^ and H^12^), 8.06 (dd, 1H, H^7^ and H^16^ or H^8^ and H^15^ or H^11^ and H^1^), 7.70 (dd, 2H, H^2^ or H^3^), 7.63 (dd, 2H, H^2^ or H^3^), 7.54 (dd, 2H, H^10^ and H^13^). ^13^C NMR (100 MHz, DMSO-*d*_6_): δ ppm 191.0 (C^1^), 162.8 (C^23^), 155.0 (C^11^ or C^12^), 154.4 (C^11^ or C^12^), 151.3 (C^4^or C^19^), 150.8 (C^4^or C^19^), 149.8 (C^27^ or C^28^), 149.2 (C^27^ or C^28^), 148.2 (C^24^ or C^31^), 148.0 (C^24^ or C^31^), 136.8 (C^2^ or C^3^), 136.5 (C^2^ or C^3^), 136.1 (C^6^or C^17^), 135.0 (C^6^or C^17^), 134.6 (C^9^ or C^14^), 134.5 (C^9^ or C^14^), 130.0, 129.9, and 129.58 (C^25^, C^26^, C^29^ and C^30^), 127.7, 127.6, and 127.6 (C^7^, C^8^, C^15^ and C^16^), 126.1 (C^5^or C^18^), 125.6 (C^5^or C^18^), 124.9 (C^10^ or C^13^), 124.6 (C^10^ or C^13^), 122.4 (C^20^), 120.3 (C^21^ or C^22^), 116.6 (C^21^ or C^22^).

#### [Ru(phen)_2_(3,5-I_2_-Sal)]BF_4_ (**7**)

Yield 94%. Anal. Calcd for C_31_H_19_BF_4_I_2_N_4_O_2_Ru: C, 40.42; H, 2.08; N, 6.08. Found: C, 40.47; H, 2.11; N, 6.09. IR (KBr, cm^–1^): 1568 (s, C=O), 1073 (s, B–F). ^1^H NMR (400 MHz, DMSO-*d*_6_): δ ppm 9.26 (s, 1H, H^1^), 9.19 (dd, 1H, H^4^ or H^19^), 8.94 (dd, 1H, H^4^ or H^19^), 8.91 (dd, 1H, H^17^ or H^6^), 8.84 (dd, 1H, H^17^ or H^6^), 8.51 (m, 2H, H^9^ and H^14^), 8.39 (m, 2H, H^18^ and H^5^), 8.29 (m, 2H, H^7^ and H^16^ or H^8^ and H^15^ or H^11^ and H^12^), 8.23 (m, 2H, H^7^ and H^16^ or H^8^ and H^15^ or H^11^ and H^12^), 8.09 (m, 2H, H^7^ and H^16^ or H^8^ and H^15^ or H^11^ and H^1^), 7.95 (dd, 1H, H^2^ or H^3^), 7.85 (dd, 1H, H^2^ or H^3^), 7.54 (m, 2H, H^10^ and H^13^). ^13^C NMR (100 MHz, DMSO-*d*_6_): δ ppm 191.0 (C^1^), 164.9 (C^23^), 155.0 (C^11^ or C^12^), 154.4 (C^11^ or C^12^), 151.3 (C^4^or C^19^), 150.8 (C^4^or C^19^), 149.8 (C^27^ or C^28^), 149.5 (C^27^ or C^28^), 149.4 (C^2^ or C^3^), 148.2 (C^24^ or C^31^), 148.1 (C^24^ or C^31^), 145.5 (C^2^ or C^3^), 136.5 (C^6^or C^17^), 136.0 (C^6^or C^17^), 134.5 (C^9^ or C^14^), 134.39 (C^9^ or C^14^), 130.0, 129.9, 129.9 and 129.5(C^25^, C^26^, C^29^ and C^30^), 127.7, 127.6, 127.6 and 127.5 6 (C^7^, C^8^, C^15^ and C^16^), 126.1 (C^5^or C^18^), 125.5 (C^5^or C^18^), 125.0 (C^10^ or C^13^), 124.5 (C^10^ or C^13^), 123.1 (C^20^), 100.4 (C^21^ or C^22^) 74.0 (C^21^ or C^22^).

#### [Ru(phen)_2_(5-NO_2_-Sal)]BF_4_ (**8**)

Yield 87%. Anal. Calcd for C_31_H_20_BF_4_N_5_O_4_Ru: C, 52.12; H, 2.28; N, 9.80. Found: C, 52.13; H, 2.30; N, 9.84.IR (KBr, cm^–1^): 1594 (s, C=O), 1062 (s, B–F). ^1^H NMR (400 MHz, DMSO-*d*_6_): δ ppm 9.52 (s, 1H, H^1^), 9.24 (d, 1H, H^5^ or H^20^), 9.08 (d, 1H, H^5^ or H^20^), 8.91 (d, 1H, H^7^ or H^18^), 8.87 (d, 1H, H^7^ or H^18^), 8.66 (d, 1H, H^2^), 8.51 (t, 2H, H^10^ and H^15^), 8.39 (dd, 2H, H^6^ and H^19^), 8.31 (m, 2H, H^8^ and H^17^ or H^9^ and H^16^ or H^12^ or H^13^), 8.19 (m, 1H, H^8^ and H^17^ or H^9^ and H^16^ or H^12^ or H^13^), 8.12 (m, 2H, H^8^ and H^17^ or H^9^ and H^16^ or H^12^ or H^13^), 8.05 (d, 1H, H^8^ and H^17^ or H^9^ and H^16^ or H^12^ or H^13^), 7.92 (dd, 1H, H^4^), 7.53 (dt, 2H, H^11^ and H^14^), 6.60 (d, 1H, H^3^). ^13^C NMR (100 MHz, DMSO-*d*_6_): δ ppm 190.3 (C^1^), 170.1 (C^23^), 153.5 (C^12^ or C^13^), 153.2 (C^12^ or C^13^), 151.1 (C^5^ or C^20^), 150.9 (C^5^ or C^20^), 149.3 (C^27^or C^28^), 148.8 (C^27^ or C^28^), 147.7 (C^24^ or C^31^), 147.2 (C^24^ or C^31^), 139.6 (C^7^ or C^18^), 139.1 (C^7^ or C^18^), 136.9 (C^4^), 135.2 (C^2^), 134.0 (C^10^ or C^15^), 133.7 (C^10^ or C^15^), 131.0, 130.9, 130.8, and 130.7 (C^25^, C^26^, C^29^ and C^30^), 127.9, 127.6, and 127.2 (C^8^, C^9^, C^16^ and C^17^), 126.1 (C^3^), 125.9 (C^6^ or C^19^), 125.5 (C^6^ or C^19^), 124.7 (C^11^ or C^14^), 124.2 (C^11^ or C^14^), 123.0 (C^21^), 116.3 (C^22^).

#### [Ru(phen)_2_(5-Me-Sal)]BF_4_ (**9**)

Yield 84%. Anal. Calcd For C_32_H_23_BF_4_N_4_O_2_Ru: C, 56.24; H, 3.39; N, 8.20. Found: C, 56.29; H, 3.42; N, 8.24. IR (KBr, cm^–1^): 1618 (s, C=O), 1058 (s, B–F). ^1^H NMR (400 MHz, DMSO-*d*_6_): δ ppm 9.16 (dd, 1H, H^20^), 9.07 (m, 2H, H^1^ and H^5^), 8.85 (dd, 1H, H^7^), 8.81 (dd, 1H, H^18^), 8.48 (dd, 1H, H^10^ or H^15^), 8.45 (dd, 1H, H^10^ or H^15^), 8.37 (m, 2H, H^8^ and H^17^), 8.27 (dd, 2H, H^9^ and H^16^), 8.16 (dd, 1H, H^6^), 8.09 (m, 2H, H^19^ and H^12^ or H^13^), 8.02 (dd, 1H, H^12^ or H^13^), 7.51 (td, 2H, H^11^ and H^14^), 7.15 (d, 1H, H^2^), 7.03 (dd, 1H, H^4^), 6.44 (d, 1H, H^3^), 2.10 (s, 3H, Me). ^13^C NMR (126 MHz, DMSO-*d*_6_): δ ppm 187.8 (C^1^), 170.8 (C^23^), 153.9 (C^12^ or C^13^), 153.4 (C^12^ or C^13^), 151.1 (C^5^ or C^20^), 149.8 (C^5^ or C^20^), 149.4 (C^27^ or C^28^), 149.1 (C^27^ or C^28^), 148.4(C^24^ or C^31^), 148.1(C^24^or C^31^), 147.2 (C^22^), 137.2 (C^2^), 136.2 (C^7^or C^18^), 135.8 (C^7^or C^18^), 133.8 (C^10^ or C^15^), 133.2 0 (C^10^ or C^15^), 130.2, 130.1, 129.9, and 129.8 (C^25^, C^26^, C^29^ and C^30^), 128.1, 127.9, and 127.9 (C^8^, C^9^, C^16^ and C^17^), 125.3 (C^6^or C^19^), 125.0 (C^6^or C^19^), 124.9 (C^11^ or C^14^), 124.4 (C^11^ or C^14^), 124.0 (C^4^), 119.3 (C^21^), 114.7 (C^3^), 21.0 (Me).

#### [Ru(phen)_2_(4-Me-Sal)]BF_4_ (**10**)

Yield 84%. Anal. Calcd For C_32_H_23_BF_4_N_4_O_2_Ru: C, 56.24; H, 3.39; N, 8.20. Found: C, 56.27; H, 3.41; N, 8.23. IR (KBr, cm^–1^): 1622 (s, C=O), 1058 (s, B–F). ^1^H NMR (500 MHz, DMSO-*d*_6_): δ ppm 9.16 (dd, *J* = 5.2, 1.2 Hz, 1H, H^20^), 9.09 (dd, *J* = 5.1, 1.3 Hz, 1H, H^5^), 9.03 (s, 1H, H^1^), 8.85 (dd, *J* = 8.3, 1.3 Hz, 1H, H^7^), 8.81 (dd, *J* = 8.2, 1.2 Hz, 1H, H^18^), 8.48 (dd, *J* = 8.3, 1.2 Hz, 1H, H^10^ or H^15^), 8.46 (dd, *J* = 8.2, 1.2 Hz, 1H, H^10^ or H^15^), 8.37 (d, *J* = 8.9 Hz, 2H, H^8^ and H^17^), 8.28 (d, *J* = 3.3 Hz, 1H, H^9^ or H^16^), 8.26 (d, *J* = 3.3 Hz, 1H, H^9^ or H^16^), 8.17 (dd, *J* = 8.2, 5.1 Hz, 1H, H^6^), 8.12–8.07 (m, 2H, H^19^ and H^12^ or H^13^), 8.02 (dd, *J* = 5.4, 1.2 Hz, 1H, H^12^ or H^13^), 7.51 (td, *J* = 8.1, 5.3 Hz, 2H, H^11^ and H^14^), 7.28 (d, *J* = 8.3 Hz, 1H, H^2^), 6.35 (s, 1H, H^4^), 6.28 (dd, *J* = 8.3, 1.5 Hz, 1H, H^3^), 1.99 (d, *J* = 0.8 Hz, 3H, Me). ^13^C NMR (126 MHz, DMSO-*d*_6_): δ ppm 188.8 (C^1^), 170.3 (C^23^), 154.6 (C^12^ or C^13^), 154.2 (C^12^ or C^13^), 151.3 (C^5^ or C^20^), 151.1 (C^5^ or C^20^), 149.9 (C^27^ or C^28^), 149.4 (C^27^ or C^28^), 148.4 (C^31^), 148.2 (C^25^), 147.0 (C^22^), 137.0 (C^2^), 136.0 (C^7^), 135.5 (C^18^), 134.2 (C^10^ or C^15^), 134.0 (C^10^ or C^15^), 130.0, 129.9, 129.9, and 129.7 (C^24^, C^26^, C^29^ and C^30^), 127.7, 127.6, and 127.6 (C^8^, C^9^, C^16^ and C^17^), 125.8 (C^6^), 125.5 (C^19^), 124.8 (C^11^ or C^14^), 124.6 (C^11^ or C^14^), 123.8 (C^4^), 120.2 (C^21^), 116.5 (C^3^), 21.4 (Me).

#### [Ru(phen)_2_(4-OMe-Sal)]BF_4_ (**11**)

Yield 90%. Anal. Calcd For C_32_H_23_BF_4_N_4_O_3_Ru: C, 54.95; H, 3.31; N, 8.01. Found: C, 54.93; H, 3.34; N, 8.05.IR (KBr, cm^–1^): 1612 (s, C=O), 1058 (s, B–F). ^1^H NMR (500 MHz, DMSO-*d*_6_): δ ppm 9.18 (dd, *J* = 5.2, 1.2 Hz, 1H, H^20^), 9.12 (dd, *J* = 5.2, 1.3 Hz, 1H, H^5^), 8.88 (s, 1H, H^1^), 8.84 (dd, *J* = 8.4, 1.3 Hz, 1H, H^7^), 8.82 (dd, *J* = 8.2, 1.2 Hz, 1H, H^18^), 8.46 (ddd, *J* = 8.2, 5.7, 1.2 Hz, 2H, H^10^ and H^15^), 8.38 (d, *J* = 6.1 Hz, 1H, H^8^ or H^17^), 8.36 (d, *J* = 6.0 Hz, 1H, H^8^ or H^17^), 8.28 (d, *J* = 2.2 Hz, 1H, H^9^ or H^16^), 8.26 (d, *J* = 2.2 Hz, 1H, H^9^ or H^16^), 8.18 (dd, *J* = 8.2, 5.2 Hz, 1H, H^6^), 8.12 (dd, *J* = 8.2, 5.2 Hz, 1H, H^19^), 8.04 (ddd, *J* = 7.6, 5.4, 1.2 Hz, 2H, H^12^ and H^13^), 7.51 (ddd, *J* = 8.1, 5.4, 1.2 Hz, 2H, H^11^ and H^14^), 7.27 (d, *J* = 9.1 Hz, 1H, H^2^), 6.10 (dd, *J* = 9.0, 2.4 Hz, 1H, H^3^), 6.02 (d, *J* = 2.4 Hz, 1H, H^4^), 3.60 (s, 3H, OMe). ^13^C NMR (126 MHz, DMSO-*d*_6_): δ ppm 187.0 (C^1^), 172.3 (C^23^), 166.1 (C^22^), 154.6 (C^12^ or C^13^), 154.3(C^12^ or C^13^), 151.2 (C^20^), 151.2 (C^5^), 150.0 (C^27^ or C^28^), 149.5 (C^27^ or C^28^), 148.4 (C^24^ or C^31^), 148.3(C^24^ or C^31^), 138.5 (C^2^), 135.9 (C^7^ or C^18^), 135.5 (C^7^ or C^18^), 134.2 (C^10^ or C^15^), 134.0 (C^10^ or C^15^), 130.1, 130.0, 129.9, and 129.8 (C^25^, C^26^, C^29^ and C^30^), 127.7, 127.6, and 127.5 (C^8^, C^9^, C^16^ and C^17^) 125.8 (C^6^ or C^19^), 125.6 (C^6^ or C^19^), 124.8 (C^11^ or C^14^), 124.7 (C^11^ or C^14^), 117.2 (C^21^), 107.3 (C^3^), 103.8 (C^4^), 55.3 (Me).

#### [Ru(phen)_2_(4-DEA-Sal)]BF_4_ (**12**)

Yield 90%. Anal. Calcd for C_35_H_30_BF_4_N_5_O_2_Ru: C, 56.77; H, 4.08; N, 9.46. Found: C, 56.83; H, 4.12; N, 9.43. IR (KBr, cm^–1^): 1611 (s, C=O), 1059 (s, B–F). ^1^H NMR (400 MHz, DMSO-*d*_6_): δ ppm 9.17 (m, 2H, H^20^ and H^5^), 8.79 (td, 2H, H^7^and H^18^), 8.52 (s, 1H, H^1^), 8.43 (d, 2H, H^10^ and H^15^), 8.34 (dd, 2H, H^8^ and H^17^), 8.25 (dd, 2H, H^9^ and H^16^), 8.18 (dd, 1H, H^6^), 8.11 (dd, 1H, H^19^), 8.01 (dd, 1H, H^12^ or H^13^), 7.96 (dd, 1H, H^12^ or H^13^), 7.48 (m, 2H, H^11^ and H^14^), 7.07(d, 1H, H^2^), 6.08 (dd, 1H, H^3^), 5.58 (d, 1H, H^4^), 3.24 (m, 4H, −(CH_2_)−), 1.00 (t, 6H, Me). ^13^C NMR (126 MHz, DMSO-*d*_6_): δ ppm 188.0 (C^1^), 171.9 (C^23^), 162.4 (C^22^), 153.8 (C^12^ or C^13^), 153.2 (C^12^ or C^13^), 151.0 (C^5^ or C^20^), 150.9 (C^5^ or C^20^), 149.7 (C^27^or C^28^), 149.4 (C^27^ or C^28^), 148.5 (C^24^ or C^31^), 148.0 (C^24^ or C^31^), 139.5 (C^2^), 133.3 (C^7^ or C^18^), 133.0 (C^7^ or C^18^), 131.8 (C^10^ or C^15^), 131.7 (C^10^ or C^15^), 130.3, 130.2 129.9 and 129.8 (C^25^, C^26^, C^29^ and C^30^), 127.9, 127.6, and 127.4 (C^8^, C^9^, C^16^ and C^17^), 126.9 (C^6^ or C^19^), 126.6 (C^6^ or C^19^), 125.3 (C^11^ or C^14^), 125.2 (C^11^ or C^14^), 123.8 (C^21^), 105.3 (C^3^), 104.1 (C^4^), 44.3 (CH_2_), 24.0 (Me).

### Single-Crystal X-ray Details

X-ray quality single-crystals for complexes **4** and **6** were obtained by slow evaporation of concentrated ethanol/acetonitrile (1:1) solution in the refrigerator after 4–5 days, while for complexes **9**, **10**, and **11** were grown by slow diffusion of diethyl ether into a concentrated solution of the sample in methanol at room temperature.

For the complexes **9**, **10**, and **11**, the single-crystal X-ray diffraction data were collected at 160(1) K on a Rigaku OD Synergy-Hypix diffractometer using the copper X-ray radiation (λ = 1.54184 Å) from a dual wavelength X-ray source and an Oxford Instruments Cryojet XL cooler. For the complexes **4** and **6** instead, these data were collected at 160(1) K on a Rigaku OD XtaLAB Synergy, Dualflex, Pilatus 200 K diffractometer using a single wavelength X-ray source (Cu Kα radiation: λ = 1.54184 Å) from a microfocus sealed X-ray tube and an Oxford liquid-nitrogen Cryostream cooler and at 160(1) K on a Rigaku OD SuperNova/Atlas area-detector diffractometer using Cu K_α_ radiation (l = 1.54184 Å) from a microfocus X-ray source and an Oxford Instruments Cryojet XL cooler, respectively.

The most suitable single-crystal was selected and mounted on a flexible loop fixed on a goniometer head using polybutene oil and immediately transferred to the diffractometer. Pre-experiment, data collection, data reduction, and analytical absorption correction^24^ were performed using the program *CrysAlisPro*(25) implemented in the *Olex2* software.^26^ The structure was solved with the SHELXT^27^ small molecule structure solution program and refined with the *SHELXL2018/3* program package^27^ by full-matrix least-squares minimization on *F*^2^. The *PLATON*(28) software program was employed to verify the results of the X-ray analysis. Further information about experimental parameters and data are reported in the CIF file.

For **9** and **10**, the ions cocrystallized with molecules of solvent (methanol). In the asymmetric unit, the solvent molecule is disordered over two sets of positions with site-occupancy factors of 0.341(6) and 0.659(6) for **10** and with site-occupancy factors of 0.341(9) and 0.659(9) for **9**. For **9**, the ions cocrystallized with molecules of methanol and diethyl ether in a ratio 1/1/0.5. A solvent mask^29^ was used in *Olex2* to account for the residual electron density ascribed to the disordered molecules of diethyl ether. Although not present in the final model, moiety formula and sum formula in the CIF include the atoms of those molecules of solvent leading to some alerts in the checkCIF report. The F atoms of the PF_6_^–^ counterion in **6** are disordered over two sets of positions with site-occupancy factors of 0.304(5) and 0.695(5). The solvent molecule of ethanol is also disordered over two sets of positions, with site-occupancy factors of 0.436(11) and 0.564(11). More details concerning the crystal structures and refinements can be found in the corresponding CIF files.

### Absorption and Emission Spectra

A Jasco V-750 spectrophotometer was used to obtain UV/vis the absorption spectra, while a Cary Eclipse fluorescence spectrophotometer with a high-precision quartz fluorescence cell was to record the emission spectra. To ensure accuracy, all samples were prepared using Schlenk techniques under an argon atmosphere. Furthermore, the emission spectra were initially measured by exciting at the maximum absorption wavelength of their corresponding UV/vis absorption spectra.

### Theoretical Calculations

Density functional theory (DFT) and time-dependent DFT (TD-DFT) calculations were performed using Becke’s three-parameter B3LYP exchange–correlation functional^30,31^ implemented ORCA 4.2.1.^32,33^ The basis sets used to define the atoms were LANL2DZ^34^ for Ru and def2-SVP^35^ for the other atoms. The empirical dispersion correction was taken into account using Grimme’s dispersion with Becke–Johnson damping, D3BJ.^36,37^ The solvent (acetonitrile) effects were considered within the self-consistent reaction field (SCRF) theory using the solvation model SMD of Truhlar et al.^38^ Time dependent DFT (TD-DFT)^39−41^ calculations of the lowest-lying 50 singlets and triplets were performed in the presence of the solvent for all complexes **1**–**12** with the minimum-energy geometry optimized for the ground state (S_0_).

### Electrochemical Measurements

The electrochemical measurements were conducted using portable potentiostat/galvanostat PalmSens equipment, which was controlled by the software PSTrace4 Version 4.4.2. All experiments were performed with a three-electrode cell configuration: the working electrode consisted of a glassy carbon-disc with a diameter of 3 mm; the auxiliary electrode was a platinum-wire electrode; the reference electrode was an Ag/AgCl (MF-2052 BASi) electrode (which was separated from the bulk solution by a Vycor frit). To remove oxygen from the solution, argon was bubbled for 5 min, after which a continuous positive flow of argon was maintained throughout the entire experiment. The cyclic voltammetry (CV) technique was employed to record the measurements of the ruthenium(II) complex solutions (5 × 10^–4^ M in acetonitrile) in the presence of [*n*Bu_4_N][PF_6_] (0.1 M) as the supporting electrolyte. The scan rate used for the CV measurements was 100 mV s^–1^ in a clockwise direction. At the end of each experiment, ferrocene was added as an internal reference to calibrate the potentials with respect to the redox pair ferrocenium/ferrocene (Fc^+^/Fc) under our experimental conditions. The potential for the Fc^+^/Fc redox couple was determined to be *E*_1/2_ = 0.40 V vs SCE.^42^

### Cell Lines and Assay Conditions

Human drug-sensitive CEM-CCRF and multidrug-resistant CEM/ADR5000 leukemia cell lines were cultured in RPMI-1640 medium supplemented with 10% fetal bovine serum and 1% penicillin/streptomycin (Invitrogen, Darmstadt, Germany). The cells were incubated in a humidified atmosphere of 5% CO_2_ in air at 37 °C. The characteristics of the multidrug-resistance phenotype of CEM/ADR5000 cells were previously described.^43^ All compounds were first dissolved in DMSO to obtain 20 mM stock solutions that were stored at −20 °C and then diluted 200-folds with the assay medium before use.

### Cell Proliferation Inhibition Assay

The antiproliferative activity of the 15 compounds was evaluated using the resazurin assay. CEM-CCRF cells were first exposed to the compounds at a fixed screening concentration (10 μM). To determine the IC_50_ values of the selected most active compounds, 10 different concentrations in the range 0.3–100 μM were used for each of compound. Both CEM-CCRF and CEM/ADR5000 suspension cells were treated immediately after seeding. After 72 h incubation, 20 μL 0.01% resazurin (Promega, Mannheim, Germany) was added to each well. Resazurin fluorescence was measured after 4 h incubation using an Infinite M2000 Pro plate reader (Tecan, Crailsheim, Germany) at Ex/Em = 550 nm/590 nm wavelength.^44,45^ Cell viability was calculated in comparison to DMSO employed as the negative control. The final concentration of DMSO in the assay medium was 0.5%. The anticancer drug cisplatin was used as the positive control. This experiment was performed in triplicate with six wells each for each concentration.

### Toxicity in Normal Cells

Peripheral blood was obtained from healthy donors and collected in plastic Monovette EDTA tubes, and the isolation of the mononuclear cells (i.e., human peripheral mononuclear cells “PBMC”) was accomplished using Histopaque (Sigma-Aldrich, St. Louis, MO, USA) as already reported.^46^ Subsequently, 3 mL of blood was cautiously layered over 3 mL Histopaque and centrifuged at 400*g* for 30 min at room temperature. The PBMC-containing layer at the interface between blood serum and Histopaque was transferred into a new tube and washed with PBS three times. The isolated cells were suspended in Panserin 413 medium (PAN-Biotech, Aidenbach, Germany) supplemented with 2.5% phytohemagglutinin M (PHA-M, Life Technologies, Darmstadt, Germany). Finally, cell viability was measured using the resazurin method as described above.

### Molecular Modeling Studies

The crystal structure of DNA duplex 5′-(dCGGAAATTACCG)2–3′, cocrystallized with the inhibitor **[Ru(bpy)**_**2**_**dppz]**^**2+**^ (PDB ID: 4E1U)^47^ was downloaded from the protein data bank,^48^ prepared by means of AutoDockTools 1.5.6,^49^ and used as the DNA duplex docking target. Two docking grids were generated by means of AutoGrid 4.2.6.^49^ Docking grids were centered on the experimental BP1- and BP2-bound conformations of **[Ru(bpy)**_**2**_**dppz]**^**2+**^. Grids sizes were both set to 60 points on each axis (grid spacing 0.375 Å).

Metal complexes were built into the Maestro GUI (graphical user interface)^50^ and optimized through 100 steps of B3LYP DFT calculations using LACVP basis set for ruthenium and 6-31G* for all the other atoms.

Docking simulations were performed using AutoDock 4.2.6.^49^ Parameters for ruthenium were added to the parameters file (atom_par Pt 2.75 0.080 12.000 −0.00110 0.0 0.0 0 −1 −1 4 # Non H-bonding). For each ligand, 200 Genetic Algorithm runs were run. Population size was set to 150 individuals; the maximum number of energy evaluations was set to 2,500,000. Rates of mutation and crossover rates were set to 0.02 and 0.8, respectively. Docking poses were clustered by their atomic rmsd values using a cutoff of 2.0 Å, and the clusters were finally ranked by their lowest binding energy. The lowest energy bound conformations for DNA-BP1/**10**, DNA-BP1/**7**, DNA-BP2/**10,** and DNA-BP2/**7** were then submitted to MD simulations that were set up and run using Desmond,^51^ partial charges of **10** and **7** were retrieved from the DFT calculations. Solvation was treated explicitly using the TIP3P water model^52^ and OPLS2005 was used as the force-field.^53^ The system was neutralized by the addition of 22 Na^+^ ions. Prior to the production stage, the four systems were relaxed using a previously reported protocol.^54^ At this point, 960 ns long simulations were run in the *NPT* ensemble at a temperature of 310 K using a Nose–Hoover chain thermostat and Martyna–Tobias–Klein barostat (1.01325 bar). Time steps for bonded, near, and far interactions were set to 2, 2, and 6 fs, respectively. Recording interval for MD trajectories was set to 480 ps. Except of the residues A6, T7, T18, and T19 for BP1-bound complexes, and G3, A4, A5, T20, A21, C22 for BP2-bound systems, non-H atoms were constrained by 1 kcal/mol. Atomic rmsd values of **10** and **7** over the MD trajectories were computed using the DNA structure for frames superimposition. Open source PyMOL v. 1.8.4.0 was used for visual inspection and to make molecular representations.

## Results and Discussion

A novel family of Ru(II) polypyridyl complexes [Ru(phen)_2_(X-Sal)]BF_4_ were prepared using a common procedure as reported in the Experimental Section.^10,22,55^ These complexes were characterized by elemental analysis, FT-IR, ^1^H NMR, and ^13^C NMR spectroscopies, and the molecular structure of complexes **4**, **6**, **9**, **10**, and **11** was studied by X-ray diffraction analysis. Characteristic IR bonds attributable to the C=O (aldehyde) and B–F (BF_4_^–^) stretching frequencies and their details are provided in the Experimental Section. The IR spectra exhibit a common characteristic band at ∼1600 cm^–1^ for ν(C=O), which shifts to lower wavelength compared to free aldehyde ligands.^56^ The main stretching frequency for the BF_4_^–^ appears at ∼1058 cm^–1^.^57^

The NMR spectra of all complexes are shown in Figures S1–S12. In the ^1^H NMR spectra, a signal for aldehyde proton (CHO) from substituted salicylaldehyde ligands is detected in the range of 8.50–9.50 ppm.^58^ The aromatic protons from substituted salicylaldehyde and phen ligands appear in the range of 5.50–7.95 and 7.45–9.24 ppm, respectively. As expected, the phen ligands are nonequivalent because the nonsymmetric nature of the salicylaldehyde ligand and the aromatic signals of the phen ligands appear as two mixed set of signals. The signals (s, 3H_methyl_) at 2.1 ppm, (s, 3H_methyl_) at 1.99 ppm, (s, 3H_methoxy_) at 3.60 ppm and (m, 4H_methine_) at 3.24 ppm, and (t, 6H_methyl_) at 1.00 ppm are attributed to aliphatic protons for **9**, **10**, **11**, and **12** complexes, respectively. The signal at ∼10.90 ppm assigned to phenolic proton (OH) disappears in the spectra of their complexes, indicating the deprotonation of the salicylaldehyde ligand and its coordination of oxygen atom to Ru(II) ion. In the ^13^C NMR spectra, 28 peaks in the range of 100–176 ppm are assigned to the aromatic carbon atoms and a signal at ∼190 ppm is ascribed to the aldehyde carbon atom for all complexes. The peaks observed at 20.95, 21.42, 55.24 and 44.25, 23.97 ppm are related to the carbon atom for –CH_3_ (**9**), –CH_3_ (**10**), –OCH_3_ (**11**), and −N(CH_2_CH_3_) (**12**) substituents, respectively. The existence of peaks related to aldehyde carbon and aliphatic carbon in the spectra of the complexes confirms the coordination substitution salicylaldehyde ligand to the metal ion.

### Stability Studies

**Figure 3:**
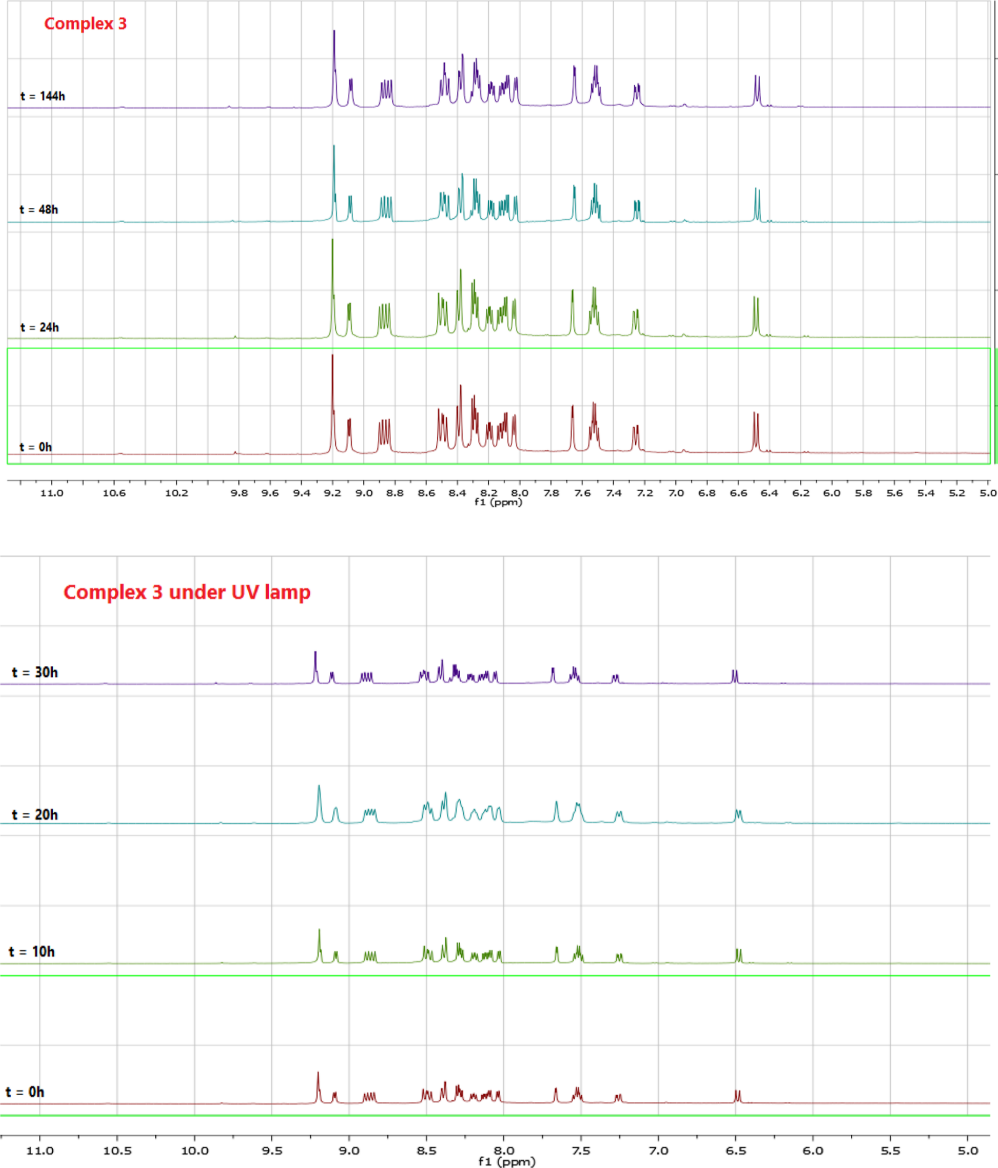
^1^H NMR spectra for the complex **3** in DMSO-*d*_6_ (up) under environmental scattered light, (down) under UV lamp [(λ = 254 nm)] at different incubation times.

To check the stability of the complexes **2**, **3**, **10**, and **11**, ^1^H NMR spectroscopy was used. The spectra were recorded in a quartz NMR tube at different incubation times in two modes, one under environmental scattered light and the other under UV lamp (λ = 254 nm). As shown in Figures 3 and S13 and S14, their stability was confirmed in both modes as no changes in the spectra were detected over time.

### X-ray Structure Analyses

**Figure 4:**
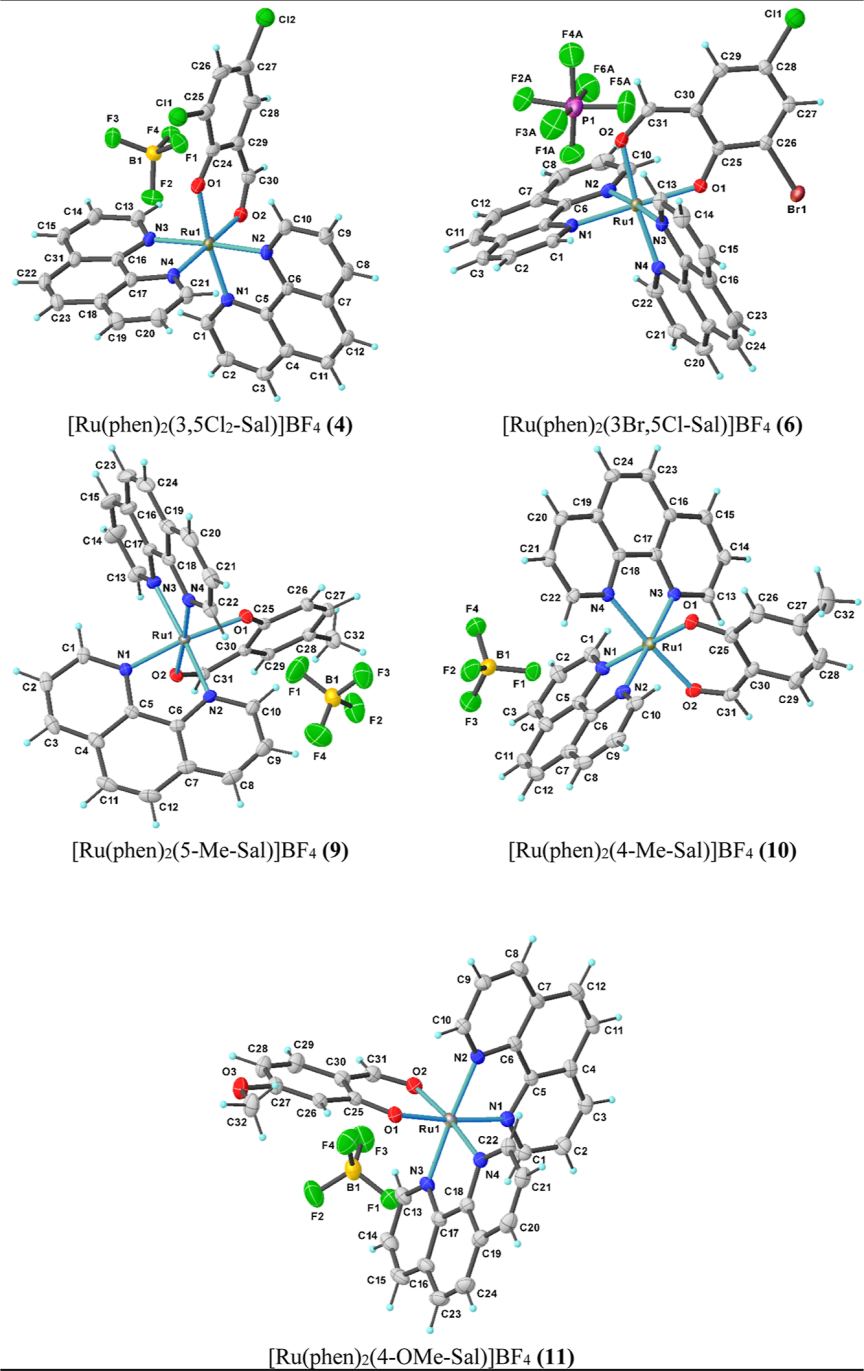
Molecular structure and atomic labeling scheme of **4**, **6**, **9**, **10**, and **11**. Thermal ellipsoids are drawn at the 50% probability level, while the hydrogen size is arbitrary. Disordered solvent molecules have been omitted for clarity.

The molecular structure complexes **4**, **6**, **9**, **10,** and **11** were confirmed by using the single-crystal X-ray diffraction technique. The X-ray molecular structure of these complexes with atom numbering scheme are shown in Figure 4. The crystallographic data and selected bond lengths and angles are displayed in Tables 1 and 2, respectively. To obtain a good crystal for structure determination, we change the counterion of complex **6** from BF_4_^–^ to PF_6_^–^, while this complex is used for biological assays with BF_4_^–^ similar to other complexes.

**Table 1:** Crystallographic Data for **4**, **6**, **9**, **10**, and **11**

	**4**	**6**	**9**	**10**	**11**
empirical formula	C_31_H_19_BCl_2_F_4_N_4_O_2_Ru	C_33_H_25_BrClF_6_N_4_O_3_PRu	C_33_H_27_BF_4_N_4_O_3_Ru	C_35_H_27_BF_4_N_4_O_3_Ru	C_35_H_32_BF_4_N_4_O_4.5_Ru
formula weight	738.28	886.97	715.46	715.46	768.52
temperature/K	160(1)	160(1)	160(1)	160(1)	160(1)
crystal system	triclinic	monoclinic	monoclinic	triclinic	triclinic
space group	*P*1̅	*P*2_1_/*n*	*P*2_1_/*n*	*P*1̅	*P*1̅
*a*/Å	10.3943(3)	14.35833(10)	10.3720(1)	10.8487(2)	11.9399(2)
*b*/Å	12.1738(4)	21.06323(19)	15.5809(1)	12.13080(10)	12.6132(2)
*c*/Å	13.4918(4)	11.02611(7)	18.9897(1)	13.1787(2)	13.6491(2)
α/deg	87.818(3)	90	90	106.9530(10)	106.2550(10)
β/deg	67.579(3)	94.3527(6)	101.407(1)	107.335(2)	115.215(2)
γ/deg	66.088(3)	90	90	95.3600(10)	101.9260(10)
volume/Å^3^	1428.93(8)	3325.04(4)	3008.21(4)	1552.46(4)	1654.91(5)
*Z*	2	4	4	2	2
ρ_calc_ g/cm^3^	1.716	1.772	1.580	1.531	1.542
μ/mm^–^^1^	6.734	7.097	4.803	4.653	4.443
*F*(000)	736.0	1760.0	1448.0	724.0	782.0
radiation	Cu K_α_ (λ = 1.54184)	Cu K_α_ (λ = 1.54184)	Cu K_α_ (λ = 1.54184)	Cu K_α_ (λ = 1.54184)	Cu K_α_ (λ = 1.54184)
2Θ range for data collection/deg	7.156 to 148.996	6.174 to 149.008	7.4 to 148.99	7.462 to 148.994	7.872 to 149
index ranges	–12 ≤ *h* ≤ 12	–17 ≤ *h* ≤ 17	–12 ≤ *h* ≤ 12	–13 ≤ *h* ≤ 13	–14 ≤ *h* ≤ 14
	–15 ≤ *k* ≤ 14	–26 ≤ *k* ≤ 26	–19 ≤ *k* ≤ 19	–15 ≤ *k* ≤ 13	–15 ≤ *k* ≤ 15
	–16 ≤ *l* ≤ 16	–12 ≤ *l* ≤ 13	–23 ≤ *l* ≤ 23	–16 ≤ *l* ≤ 16	–17 ≤ *l* ≤ 17
reflections collected	30,162	34,292	30,950	32,154	34,392
independent reflections	5823 [*R*_int_ = 0.0470]	6791 [*R*_int_ = 0.0249]	6152 [*R*_int_ = 0.0171]	6328 [*R*_int_ = 0.0290]	6748 [*R*_int_ = 0.0203]
data/restraints/parameters	5823/0/406	6791/586/542	6152/38/439	6328/38/439	6748/0/427
goodness-of-fit on *F*^2^	1.053	1.039	1.038	1.074	1.033
final *R* indexes [*I* ≥ 2σ (*I*)]	*R*_1_ = 0.0388	*R*_1_ = 0.0361	*R*_1_ = 0.0258	*R*_1_ = 0.0349	*R*_1_ = 0.0279
	w*R*_2_ = 0.1052	w*R*_2_ = 0.1005	w*R*_2_ = 0.0659	w*R*_2_ = 0.0941	w*R*_2_ = 0.0761
final *R* indexes [all data]	*R*_1_ = 0.0483	*R*_1_ = 0.0386	*R*_1_ = 0.0270	*R*_1_ = 0.0355	*R*_1_ = 0.0281
	w*R*_2_ = 0.1121	w*R*_2_ = 0.1033	w*R*_2_ = 0.0669	w*R*_2_ = 0.0945	w*R*_2_ = 0.0763
largest diff. peak/hole/e Å^–^^3^	0.98/–0.80	1.22/–0.64	0.43/–0.75	1.15/–0.68	0.60/–0.66
CCDC number	2204680	2204679	2166691	2166690	2166692

**Table 2:** Selected Bond Lengths (Å) and Angles (deg) for **4**, **6**, **9**, **10**, and **11**

	**4**		**6**		**9**		**10**		**11**	
	XRD	DFT	XRD	DFT	XRD	DFT	XRD	DFT	XRD	DFT
Ru1–N1	2.034(3)	2.076	2.038(3)	2.076	2.0357(16)	2.079	2.047(2)	2.079	2.0394(16)	2.078
Ru1–N2	2.053(3)	2.089	2.059(3)	2.088	2.0523(17)	2.090	2.051(2)	2.089	2.0573(16)	2.089
Ru1–N3	2.062(3)	2.093	2.048(3)	2.094	2.0490(17)	2.090	2.054(2)	2.090	2.0458(17)	2.090
Ru1–N4	2.032(3)	2.066	2.033(3)	2.065	2.0394(16)	2.067	2.033(2)	2.067	2.0325(17)	2.068
Ru1–O1	2.072(3)	2.085	2.051(2)	2.084	2.0526(14)	2.084	2.0548(18)	2.085	2.0643(13)	2.087
Ru1–O2	2.059(3)	2.099	2.059(2)	2.099	2.0594(13)	2.097	2.0636(18)	2.098	2.0649(14)	2.099
N1–Ru1–N2	80.17(12)	79.9	80.11(11)	79.9	80.72(6)	79.8	80.23(9)	79.8	80.20(7)	79.8
N1–Ru1–N3	98.07(12)	97.9	97.44(10)	98.2	94.82(7)	97.7	99.86(8)	97.8	96.27(7)	97.8
N1–Ru1–O1	172.66(11)	173.6	174.23(10)	173.8	173.43(6)	173.8	173.69(7)	173.8	174.17(6)	173.8
N1–Ru1–O2	85.56(11)	89.9	92.08(9)	89.9	85.07(6)	89.7	90.76(8)	89.6	88.98(6)	89.6
N2–Ru1–N3	175.88(12)	175.9	176.11(11)	176.1	173.83(7)	175.9	178.34(7)	176.0	176.23(6)	176.1
N2–Ru1–O1	92.93(11)	93.7	94.96(10)	94.0	93.57(6)	94.0	94.04(9)	94.0	93.98(6)	94.0
N2–Ru1–O2	88.82(11)	88.8	89.39(10)	88.7	89.21(6)	88.5	87.11(8)	88.5	87.82(6)	88.5
N3–Ru1–O1	88.98(11)	88.6	87.32(9)	87.9	91.11(6)	88.5	85.79(8)	88.4	89.54(6)	88.4
N3–Ru1–O2	94.77(11)	94.7	93.73(10)	94.7	94.69(6)	94.7	94.55(8)	94.7	93.47(7)	94.6
N4–Ru1–N1	91.01(12)	92.0	93.51(10)	92.4	94.90(6)	91.6	90.24(8)	91.8	94.24(6)	91.9
N4–Ru1–N2	96.05(12)	96.6	96.60(10)	96.7	95.53(7)	96.9	97.74(8)	96.9	98.47(7)	97.0
N4–Ru1–N3	80.22(12)	80.0	80.48(11)	80.0	80.52(7)	80.0	80.61(8)	79.9	80.39(7)	79.9
N4–Ru1–O1	92.24(12)	88.8	84.02(9)	88.4	88.85(6)	88.7	87.91(8)	88.6	86.28(6)	88.5
N4–Ru1–O2	173.49(11)	174.5	172.42(10)	174.4	175.19(6)	174.6	175.15(7)	174.6	173.33(6)	174.5
O1–Ru1–O2	91.82(11)	89.9	90.87(9)	89.9	91.64(5)	90.6	91.58(8)	90.6	91.11(6)	90.6

All these complexes have similar structures. The compounds **4**, **10**, and **11** were crystallized in the triclinic system with and *P*1̅ space group and compounds **6** and **9** crystallized in the monoclinic system with the *P*2_1_/*c* and P2_1_/*n* space groups, respectively.

The crystallographic data feature that the central metal ion is six-coordinated by four nitrogen atoms of two phen ligands and the aldehyde-*O* and phenol-*O* atoms from the deprotonated aldehyde ligand, making a virtually planar five and six-membered chelate ring in the distorted octahedral geometry. This structural characteristics are perfectly consistent with what has been observed for our previous set of related polypyridyl Ru(II) complexes Δ/Λ-[Ru(bpy)_2_(X,Y-Sal)]BF_4_.^22^

The Ru–N_phen_ bond lengths are in the range of 2.032(3)–2.062(3) Å, 2.033(3)–2.059(3) Å, 2.0357(16)–2.0523(17) Å, 2.033(2)–2.054(2) Å, and 2.0325(17)–2.0573(16) Å for complexes **4**, **6**, **9**, **10**, and **11**, respectively.

The shortest Ru–N bond lengths (Ru1–N1 and Ru1–N4, see Table 2) are those in which the pyridine ring nitrogen (N1 and N4) are trans to the oxygen atoms of substituted salicylaldehyde (O1 and O2), and this is consistent with the improved Ru(dπ)–phen(π*) back-bonding, which is consequent to the increase of electron density at the Ru(II) center due to the strong σ-donor effect of the deprotonated substituted salicylaldehyde ligand.

The Ru–O_aldehyde_ bond lengths are 2.059(3), 2.059(2), 2.0594(13), 2.0636(18), and 2.0649(14) Å for **4**, **6**, **9**, **10**, and **11**, respectively. The Ru–O_phenol_ bond lengths are 2.072(13), 2.052(2), 2.0526(14), 2.0548(18), and 2.0643(13) Å for **4**, **6**, **9**, **10** and **11**, respectively. The Ru–N bond trans to the Ru–O_phenol_ bond (for **4**: 2.034(3) Å, **6**: 2.039(3) Å, **9**: 2.0357(16) Å, **10**: 2.047(2) Å and **11**: 2.0394(16) Å) is slightly different than the Ru–N bond trans to Ru–O_aldehyde_ bond (**4**: 2.032(3) Å, **6**: 2.033(3) Å, **9**: 2.0394(16) Å, **10**: 2.033(2) Å, and **11**: 2.0325(17) Å.

The bite angles of the O(1)–Ru–O(2) for complexes **4**, **6**, **9**, **10**, and **11**, respectively, are 91.82(11)°, 90.86(9)°, 91.64(5)°, 91.58(8)°, and 91.11(6)°, which corresponds well with the bite angle obtained of 90.26(13)° for [Ru(bpy)_2_(Br-Sal)]BF_4_ and of 91.10(17)° for [Ru(bpy)_2_(Cl_2_–Sal)].BF_4_ in our previous work.^22^ The most obvious distortion of the ideal octahedral geometry results from the constrained N–Ru–N bite angles of the phen ligands, which are near 80° for all complexes (Table 2). It should be noted that bite angles near to 80° for phen ligands are usual for this class complex that is because of geometrical requirements of the chelate rings formed by the phen ligands.^22,59^

### Photophysical Properties

**Figure 5:**
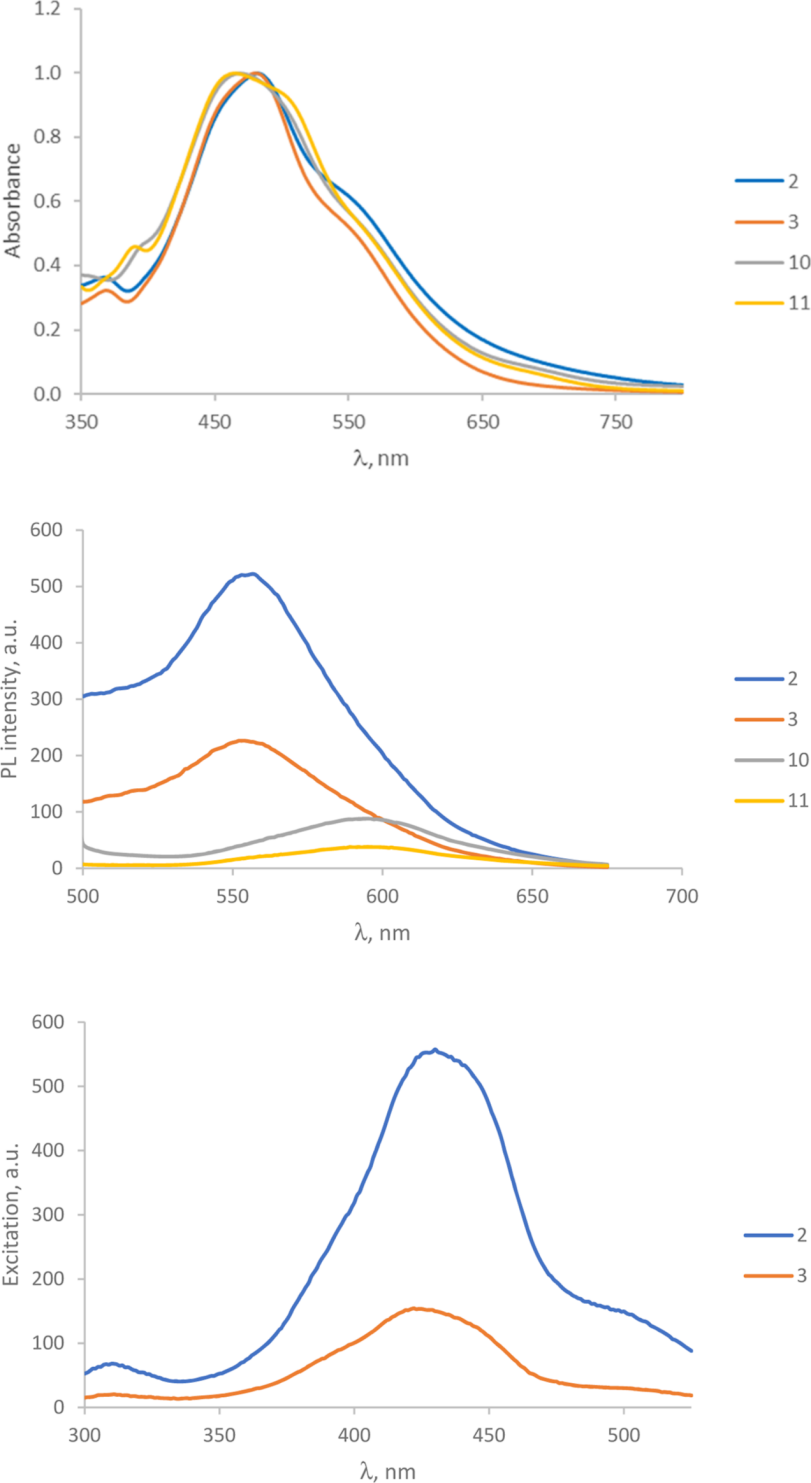
(Up) absorption spectra of 100 μM of all complexes in DMSO. (Middle) emission spectra of complexes (100 μM) in DMSO at λ_ex_ = 475 nm. (Down) scan of excitation spectra of complexes **2** and **3** in DMSO (100 μM) under fixed λ_em_ = 555 nm.

The absorption and emission spectra of compounds **2**, **3**, **10**, and **11** recorded in DMSO at room temperature as examples of all compounds are shown in Figure 5 (up). The corresponding photophysical data for these complexes are collected in Table 3. The weakest absorption bands are observed in the visible region (460–480 nm) which are attributed to MLCT transitions.^60^ Taking compound **10** as a reference, complexes **2** and **3** (with halogen in the ligand) show a bathochromic shift, while compound **11** (with methoxy-substituent) show a hypsochromic shift. Regarding the light emission, excitation of the solutions of the four compounds **2**, **3**, **10**, and **11** at 475 nm resulted in a weak visible-light emission especially for complex **11** which appears almost quenched [Figure 5 (middle)]. For compounds **2** and **3** with higher emission, the excitation spectra were scanned by fixing the λ_em_ in 550 nm to find λ_ex_ which gives us the maximum emission intensity [Figure 5 (down)]. As shown in Figure 5 (down), the λ_ex_ is about 430 nm. The values of the emission spectra are collected in Table 3 and are attributed to ^3^MLCT/^3^LLCT (see theoretical calculations). The comparison between these photophysical results (both absorption and emission) and those of the related complexes with bipyridine ligands published by us^22^ reveals a hypsochromic shift in the phenanthroline derivatives. The TD-DFT calculations (see below) show that absorptions in both series of complexes are transitions from the metal to π*-molecular orbitals of the bipyridine or phenanthroline ligands, and the energy of the transitions is higher in the case of the complexes bearing phenanthroline ligands.

**Table 3:** Photophysical Properties for Compounds **2**, **3**, **10**, and **11** Recorded in DMSO (10^–5^ M) at Room Temperature under Nitrogen Atmosphere with λ_ex_ = 475 nm

compound	λ_abs_ (nm)	λ_em_ (nm)
**2**	478	557
**3**	479	554
**10**	471	596
**11**	464	595

### Theoretical Calculations

DFT and TD-DFT calculations were carried out on the cationic of complexes (without consideration of the anion) to get a deeper understanding of their electrochemical and photophysical properties. Calculations were developed at the B3LYP/def2-SVP + LANL2DZ) level including solvent (DMSO) effects (see Experimental Section for calculation details). Table 2 gathers some calculated structural values of the computed complexes in their electronic ground states (S_0_), and the comparison with the experimental XRD values showing a good agreement between bond distances, angles, and torsional angles validating the level of theory.

**Figure 6:**
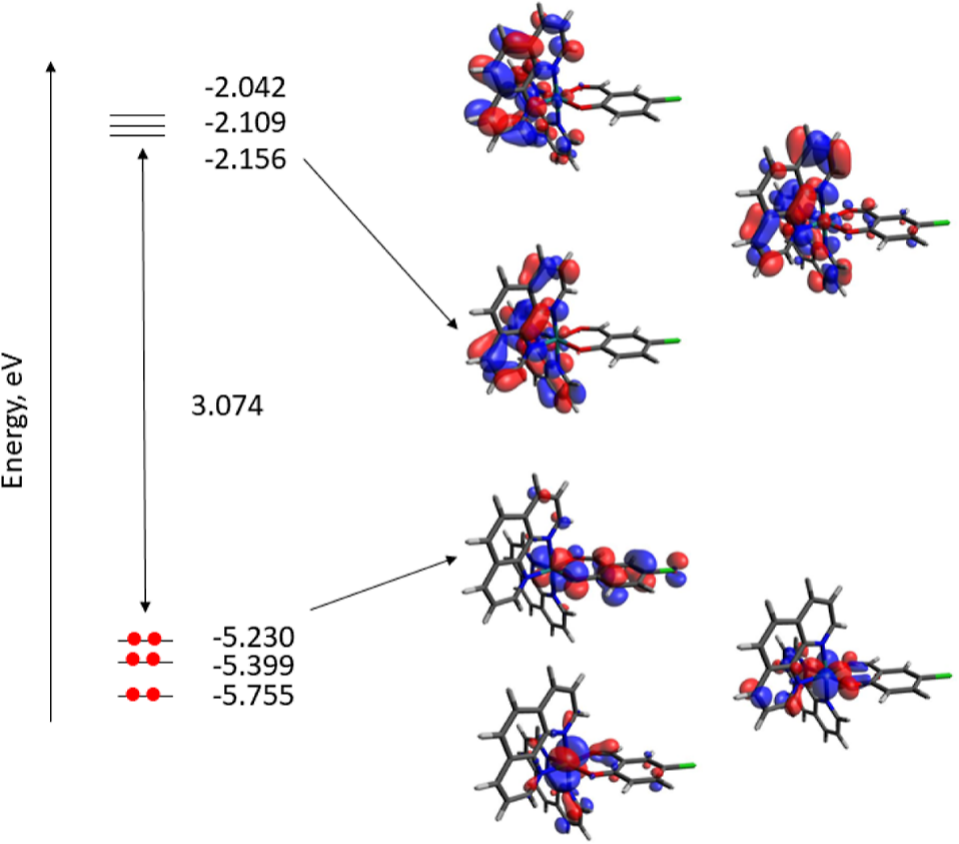
Energy levels and isosurface contour plots (0.03 au) for cation of compound **2**.

Figure 6 displays the isovalue contour plots calculated for the frontier molecular orbitals (MOs) at the electronic ground state (S_0_) of the cationic part of compound **2**. The electronic structure of complexes **1** and **3**–**12** is very similar to the one calculated for compound **2** (see Figures S15–S25). In all of them, the HOMO–LUMO gap is ranging between 2.97 and 3.27 eV. In compound **2** (as a representative example), the HOMO is contributed by the orbitals of the ruthenium atom (47.3%) and the salicylaldehyde ligand (42.9%), while the LUMO, LUMO + 1, and LUMO + 2 are mainly spread over the phenanthroline ligands (see Table S1) in a similar manner as it has been described for related complexes of ruthenium with bipyridine ligands and a chelating oxygen donor ligand.^22,61−64^

The nature of the excited states was investigated using the TD-DFT approach. The low-lying singlet and triplet states with the geometries of the ground state were calculated using this approach. Tables 4 and S2–S4 summarize the calculated excited states. As a representative example, for compound **2**, the absorption in the experimental spectrum (Figure 5 up) appeared at 478 nm is assigned to the singlet excited state S_13_ (423.2 nm) and it is a complex transition mainly composed by transitions from the HOMO – 2 to the LUMO, LUMO + 1, and LUMO + 3, and a transition HOMO – 1 → LUMO + 2, with a calculated oscillator strength of 0.1501. This band shows a shoulder at 537 nm assigned to the singlet excited state S_11_ (456.4 nm) which is mainly a double transition HOMO – 2 → LUMO and HOMO – 1 → LUMO + 3 with a calculated oscillator strength of 0.1146. For both excited states, these transitions correspond to a metal-to-ligand charge transfer (^1^MLCT) from the ruthenium center to the phenanthroline ligands. Lower energy singlet excited states displayed very low values of the oscillator strength. Similar results can be observed with compounds **3**, **10**, and **11**.

**Table 4:** Selected Singlet and Triplet Excited States Calculated at the TDDFT B3LYP/(def2-SVP + LANL2DZ) Level for Complex **2**^**+**^ in DMSO Solution^a^

complex	state	energy (eV)	λ (nm)	f.osc.	monoexcitations	nature	description
2	S_1_	2.200	563.6	0.0004	HOMO → LUMO (85)	d_π_(Ru) + π_sal_ → π*_phen_	^1^MLCT/^1^LLCT
	S_2_	2.228	556.4	0.0036	HOMO → LUMO + 1 (67)	d_π_(Ru) + π_sal_ → π*_phen_	^1^MLCT/^1^LLCT
	S_3_	2.252	550.6	0.0212	HOMO– 1 → LUMO (70)	d_π_(Ru) → π*_phen_	^1^MLCT
					HOMO– 1 → LUMO + 1 (15)	d_π_(Ru) → π*_phen_	^1^MLCT
	S_4_	2.324	533.4	0.0140	HOMO → LUMO + 1 (16)	d_π_(Ru) + π_sal_ → π*_phen_	^1^MLCT/^1^LLCT
					HOMO → LUMO + 2 (44)	d_π_(Ru) + π_sal_ → π*_phen_	^1^MLCT/^1^LLCT
					HOMO → LUMO + 3 (25)	d_π_(Ru) + π_sal_ → π*_phen_	^1^MLCT/^1^LLCT
	S_5_	2.338	530.4	0.0059	HOMO– 1 → LUMO + 1 (42)	d_π_(Ru) → π*_phen_	^1^MLCT
					HOMO → LUMO + 2 (39)	d_π_(Ru) + π_sal_ → π*_phen_	^1^MLCT/^1^LLCT
	S_11_	2.717	456.4	0.1146	HOMO– 2 → LUMO (49)	d_π_(Ru) → π*_phen_	^1^MLCT
					HOMO– 1 → LUMO + 3 (15)	d_π_(Ru) → π*_phen_	^1^MLCT
	S_13_	2.930	423.2	0.1501	HOMO– 2 → LUMO (17)	d_π_(Ru) → π*_phen_	^1^MLCT
					HOMO– 2 → LUMO + 1 (29)	d_π_(Ru) → π*_phen_	^1^MLCT
					HOMO– 2 → LUMO + 3 (19)	d_π_(Ru) → π*_phen_	^1^MLCT
					HOMO– 1 → LUMO + 2 (15)	d_π_(Ru) → π*_phen_	^1^MLCT
	T_1_	1.853	669.1		HOMO– 1 → LUMO (41)	d_π_(Ru) → π*_phen_	^3^MLCT
					HOMO → LUMO (25)	d_π_(Ru) + π_sal_ → π*_phen_	^3^MLCT/^3^LLCT
					HOMO → LUMO + 1 (19)	d_π_(Ru) + π_sal_ → π*_phen_	^3^MLCT/^3^LLCT
	T_2_	1.909	649.5		HOMO → LUMO (16)	d_π_(Ru) + π_sal_ → π*_phen_	^3^MLCT/^3^LLCT
					HOMO → LUMO + 1 (49)	d_π_(Ru) + π_sal_ → π*_phen_	^3^MLCT/^3^LLCT
	T_3_	1.991	622.7		HOMO → LUMO + 1 (18)	d_π_(Ru) + π_sal_ → π*_phen_	^3^MLCT
					HOMO → LUMO + 4 (73)	d_π_(Ru) + π_sal_ → π*_sal_	^3^MLCT/^3^LC

a Vertical excitation energies (*E*), dominant monoexcitations with contributions (within parentheses) of >15%, the nature of the electronic transition, and the description of the excited state are summarized.

The emission spectra for complexes **2**, **3**, **10**, and **11** are shown in Figure 5 (middle). The theoretical values are underestimated, especially for complexes **10** and **11**. For complex **2**, the unstructured signal at 557 nm is assigned to the calculated excited state T_1_ (669.1 nm) which corresponds mainly to transitions from the HOMO – 1 and the HOMO to the LUMO and the LUMO + 1. Since the HOMO – 1 is mainly located on the ruthenium center and the HOMO is located on the ruthenium center and salicyl ligand (while the LUMO and the LUMO + 1 are located on the phenanthroline ligands), this transition can be described as a metal-to-ligand charge transfer (^3^MLCT) along with a ligand-to-ligand charge transfer (^3^LLCT). Compound **3** shows a similar unstructured signal at a slightly higher energy than compound **2** (554 nm). This band can be assigned to the excited state T_1_, although the calculated energy is underestimated (667.8 nm, see Table S2). This excited state corresponds to transitions HOMO → LUMO and LUMO + 1 → LUMO + 2. These transitions can be described as a combination ^3^MLCT/^3^LLCT. Compound **10** displays a broad band at about 596 nm that can be assigned to the excited state T_1_. As in compound **2**, this excited state corresponds to transitions HOMO – 1 → LUMO and HOMO → LUMO + 1 also described as ^3^MLCT/^3^LLCT. Compound **11** shows a broad and weak band at 595 nm. This band can be assigned to the calculated excited state T_2_ (681.0 nm, see Table S4) and corresponds to transitions HOMO → LUMO and HOMO → LUMO + 1 (^3^MLCT/^3^LLCT).

### Electrochemical Measurements

**Figure 7:**
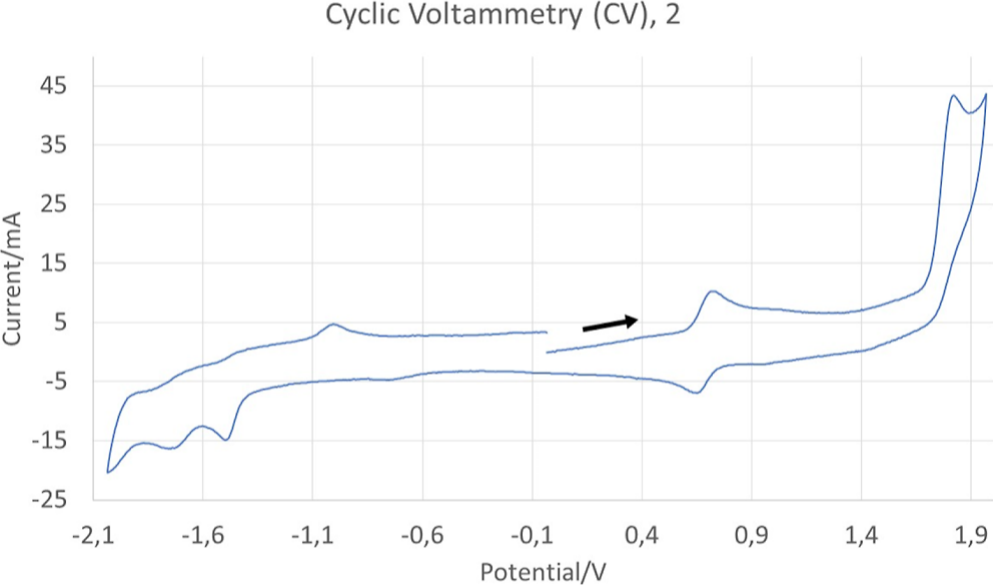
Cyclic voltammogram of compound **2** in acetonitrile solution (5 × 10^–4^ M) recorded with a scan rate of 0.10 V·s^–1^. The arrow indicates the starting point and the sense of the scan.

The electrochemical properties of compounds **2**, **3**, **10**, and **11** were examined by cyclic voltammetry (CV) in acetonitrile solutions (5 × 10^–4^ M) using [^*n*^Bu_4_N][PF_6_] (0.1 M) as the supporting electrolyte and a three-electrode setup, which incorporates a glassy carbon working electrode. The solutions were deaerated by bubbling argon. Potentials are given versus the ferrocenium/ferrocene (Fc^+^/Fc) couple and the resulting cyclic voltammograms are shown in Figures 7 and S26–S28 (see data in Table 5).

**Table 5:** Cyclic Voltammetry Data for Compounds **2**, **3**, **10**, and **11** versus Fc^+^/Fc in Acetonitrile Solution (5 × 10^–4^ M)^a^

compound	*E*_1/2_^ox^ (V)	*E*_1/2_^red1^ (V)	*E*_1/2_^red2^ (V)
**2**	+0.69 (r)	–1.50 (ir)	–1.73 (ir)
**3**	+0.68 (r)	–1.49 (ir)	–1.77 (ir)
**10**	+0.62 (r)	–1.55 (ir)	–1.79 (ir)
**11**	+0.61 (r)	–1.56 (qr)	–1.80 (qr)

a Measured using 0.1 M [^*n*^Bu_4_N][PF_6_] as the supporting electrolyte and a scan rate of 0.10 V·s^–1^ (r = reversible, qr = quasi-reversible, ir = irreversible). *E*°(Fc/Fc^+^) = 0.400 V vs SCE.

The anodic region of the voltammograms features a single reversible wave of at *E*_1/2_^ox^ = 0.61–0.69 V. It is known that for Ru(II) complexes, the first oxidation process is normally centered onto the metal^65^ For these compounds, it is reasonable to admit that the oxidation process can involve both the ruthenium center and the salicylaldehyde ligand as the HOMO is spread over this ligand and the metal. In addition, the experimental values of *E*_1/2_^ox^ are in good agreement with the calculated values of the energies of the HOMOs of these compounds because complexes with a more negative calculated value of the energy of the HOMO (**2** and **3**) display higher values of *E*_1/2_^ox^. All complexes display two irreversible or quasi-reversible reduction waves that are involving the phenanthroline ligands as the LUMO is mainly centered in these chelating ligands.^65,66^ The small additional anodic peak that appears in compounds **2**, **3**, and **10** can be due to the formation of a new product after the irreversible reduction process. This peak does not appear in compound **11** because the reductions are quasi-irreversible and the new product is not formed.

### Biological Assessments

**Figure 8:**
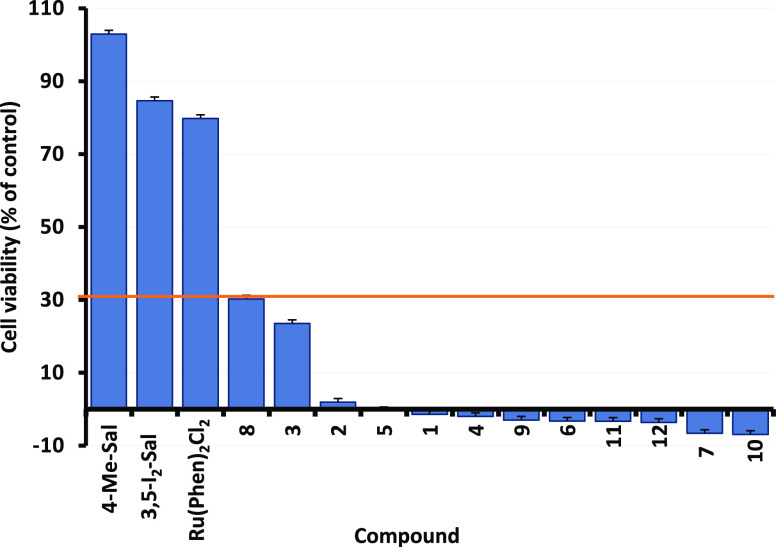
Cytotoxicity (depicted as % of residual cell viability) of the 15 compounds toward CCRF-CEM leukemic cells at 10 μM as measured by the resazurin reduction method. All data are presented as mean ± SE of three independent experiments.

On the basis of our ongoing research work dealing with Ru-based complexes,^22,67^ we determined to evaluate this newly synthesized panel of compounds as anticancer agents. Specifically, we employed two leukemic cell lines well established in our previous works, namely CCRF-CEM (drug-sensitive) and CEM/ADR5000 (CCRF-CEM multidrug-resistant subcell line). The compounds underwent a preliminary screening (resazurin method) at 10 μM against the drug-sensitive cell line CCRF-CEM. Two auxiliary salicylaldehyde ligands with different pattern of substitution and the starting **Ru(phen)**_**2**_**Cl**_**2**_ complex were selected as controls. We did not use the standard Ru-based complex **NAMI-A** as a positive control as our previous studies demonstrated that this compound exerts negligible cytotoxic effects against the two selected leukemic cell lines.^67^Figure 8 shows the screening results sorted by decreasing cell viability (waterfall plot). From this screening test emerged that all Ru-complexes but **Ru(phen)**_**2**_**Cl**_**2**_ exert an outstanding antiproliferative activity (cutoff point of 30% cell viability), highlighting the essential role of the presence of the auxiliary salicylaldehyde ligand for the biological activity. Additionally, this test evidenced the importance of the substitution pattern at this ligand as in the case of the 5-NO_2_-derivative (**8**) and 5-Br-derivative (**3**) some residual cell viability (∼30 and ∼23%, respectively) was detected. Overall, we can state that compounds bearing a 4-EDG and 3,5-dihalogen substitution pattern at the auxiliary ligand show a superior biological activity profile as anticancer agents. No significant cytotoxic activity was detected for both auxiliary salicylaldehyde ligands. Data are summarized in Table 6.

**Table 6:** Results of the Screening Assay at 10 μM against CCRF-CEM Leukemic Cells Reported as % of Residual Cell Viability

compound	cell viability % (±SD)
**1**	–1.02 ± 2.01
**2**	1.93 ± 1.74
**3**	23.51 ± 1.83
**4**	–0.25 ± 1.62
**5**	–0.40 ± 5.03
**6**	–0.58 ± 1.10
**7**	–3.11 ± 1.43
**8**	30.27 ± 3.03
**9**	–2.98 ± 1.39
**10**	–3.06 ± 0.96
**11**	–2.74 ± 1.64
**12**	–1.05 ± 0.40
**Ru(phen**_**)2**_**Cl**_**2**_	79.79 ± 2.33
4-Me-Sal	97.13 ± 3.20
3,5-I_2_-Sal	84.68 ± 5.75

**Figure 9:**
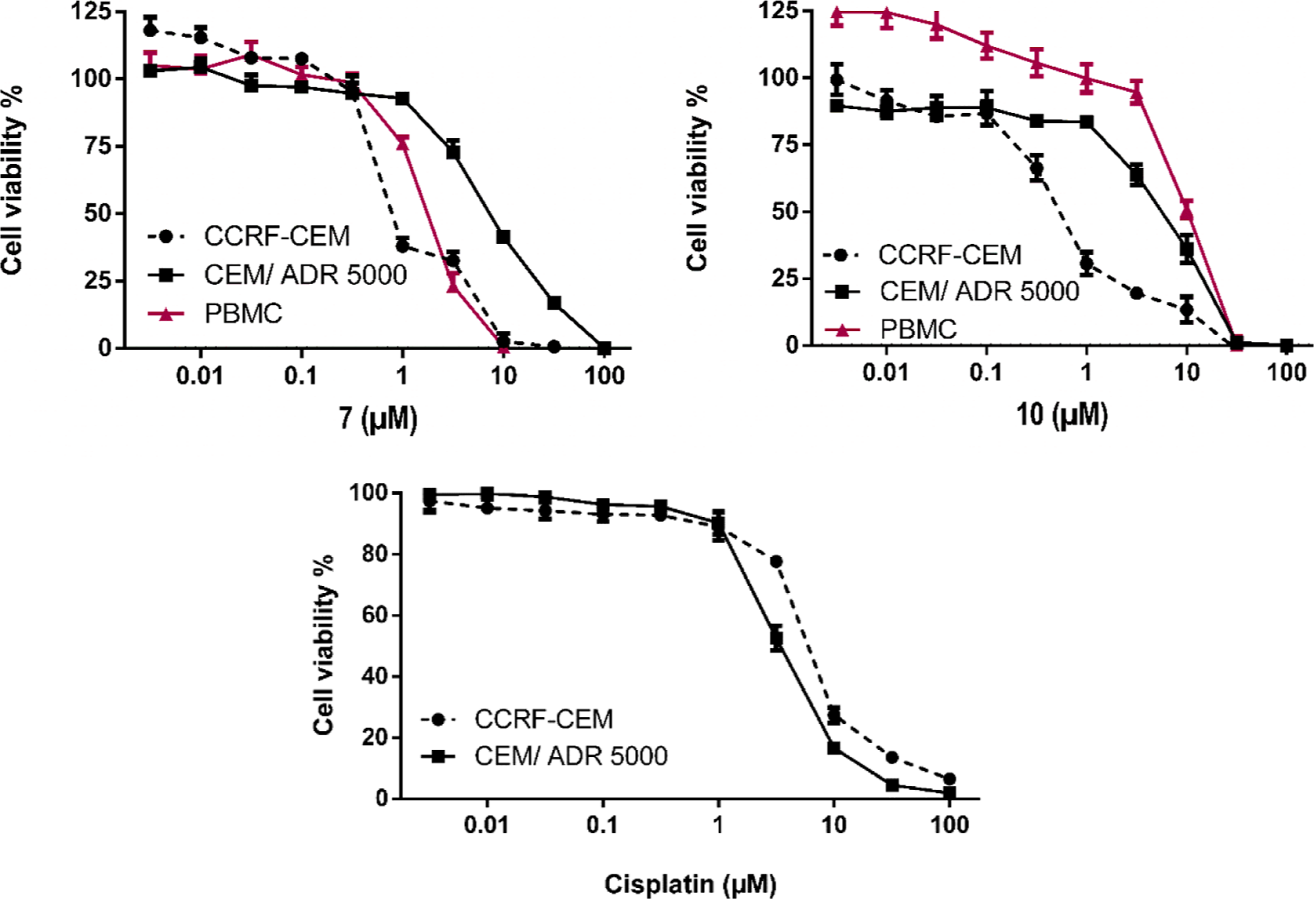
Cytotoxicity profile of the top two most active compounds **7** and **10** toward drug-sensitive parental CCRF-CEM tumor cells and their *P*-glycoprotein (MDR1/ABCB1)-expressing, multidrug-resistant subline CEM/ADR5000 as determined by resazurin reduction assays. Moreover, human peripheral mononuclear cells (PBMC) were investigated as normal counterparts to the leukemia cell lines. Cisplatin was used as a positive control to verify the multidrug resistance phenotype of the CEM/ADR5000 cells. All data are presented as mean ± SE of three independent experiments.

The top two compounds, i.e., the 4-Me-derivative (**10**) and 3,5-I_2_-derivative (**7**), were selected for continuous assays (IC_50_ determination), calculation of resistance ratio, and selectivity index [SI; evaluated on human peripheral blood mononuclear cells (PBMC)]. The dose–response curves of these two top compounds and the reference drug (cisplatin) are shown in Figure 9,^68^ and on the basis of these, their IC_50_ values (Table 7) were calculated.

**Table 7:** Cytotoxicity Data of **7** and **10** toward Drug-Sensitive CCRF-CEM, Multidrug-Resistant CEM/ADR5000, and Healthy PBMC Cells Determined by Resazurin Reduction Assay^a^

compound	CCRF-CEM		CEM/ADR5000		PBMC		degree of resistance
	IC_50_ (μM)	SD	IC_50_ (μM)	SD	IC_50_ (μM)	SD	
**7**	0.78	0.02	7.17	0.53	1.72	0.09	9.19
**10**	0.52	0.08	5.56	1	10.15	0.73	10.69
cisplatin	5.82	0.16	3.28	0.41			0.56

a All values are expressed as mean ± standard deviation (SD) of three independent experiments. The degree of resistance was calculated by dividing the IC_50_ value of resistant cells by that of sensitive cells.

Both selected Ru(II) complexes displayed antiproliferative activity in the submicromolar range against CCRF-CEM cells and in the micromolar range against CEM/ADR5000 cells. Then, the IC_50_ values have been used to calculate the degrees of resistance. Remarkably, the cross-resistance of CEM/ADR5000 to the selected two compounds was much less to that of the reference drug. Specifically, the degrees of resistance were 9.19- and 10.69-fold for **7** and **10**, respectively. Interestingly, in the cytotoxicity assays performed on PBMC, these two compounds displayed IC_50_ values higher than those measured in sensitive wild-type CCRF leukemia cells, i.e., 1.72 ± 0.09 μM (SI = 2.2) and 10.08 ± 0.65 μM (SI = 19.5) for **7** and **10**, respectively. The 4-Me-derivative (**10**) displayed also higher IC_50_ values toward PBMC as compared to multidrug-resistant CEM/ADR5000 leukemia cells (10.08 μM vs 5.56 μM), whereas the 3,5-I_2_-derivative (**7**) revealed IC_50_ values toward PBMC that were between those of the two leukemic cells (1.72 μM_PBMC_ vs 0.78 μM_CCRF-CEM_ and 7.17 μM_CEM/ADR5000_). This indicates that these two compounds may show some tumor specific inhibition (Table 7).

An approximate comparison between the biological outcomes achieved in this work with those of previous work dealing with related polypyridyl Ru(II) complexes (Δ/Λ-[Ru(bpy)_2_(X,Y-Sal)]BF_4_) suggests that this type of compounds may have a better applicability as anticancer agents in the treatment of hematological malignancies with respect to solid tumors as we obtained submicromolar IC_50_ values toward the drug-sensitive strain of leukemia cells (CCRF-CEM) and SI up to 19.5 (evaluated on PBMC cells) for the [Ru(phen)_2_(X-Sal)]BF_4_-type complexes of the present work versus low-micromolar IC_50_ values toward A2780 (ovarian carcinoma), A549 (lung carcinoma), and SW480 (colon adenocarcinoma) cells and SI up to 6.1 (evaluated on HeK293 cells) for the Δ/Λ-[Ru(bpy)_2_(X,Y-Sal)]BF_4_-type complexes of the previous work.^22^ With regards to the influence of the substitution pattern at the auxiliary salicyl ligand on the antiproliferative activity, a straightforward comparison between the two sets of compounds cannot be done as previous work dealt only with halogens and evidenced the trend dihalogenated > monohalogenated and Br > Cl for the Δ/Λ-[Ru(bpy)_2_(X,Y-Sal)]BF_4_-type complexes, whereas a more exhaustive substitution pattern was present in this work for the [Ru(phen)_2_(X-Sal)]BF_4_-type complexes. Previous work also indicated that these polypyridyl Ru(II) complexes are able to induce cell cycle arrest in the G0/G1 phase and apoptosis and that only those ones bearing Br as a substituent at the auxiliary ligand are related with increase of ROS levels and mitochondrial dysfunction.^22^ Since the new complexes are very much alike to the former ones, we determined not to repeat these assays and move forward with the molecular docking studies (see hereinafter) on the intended DNA target.

### Molecular Modeling Studies

**Figure 10:**
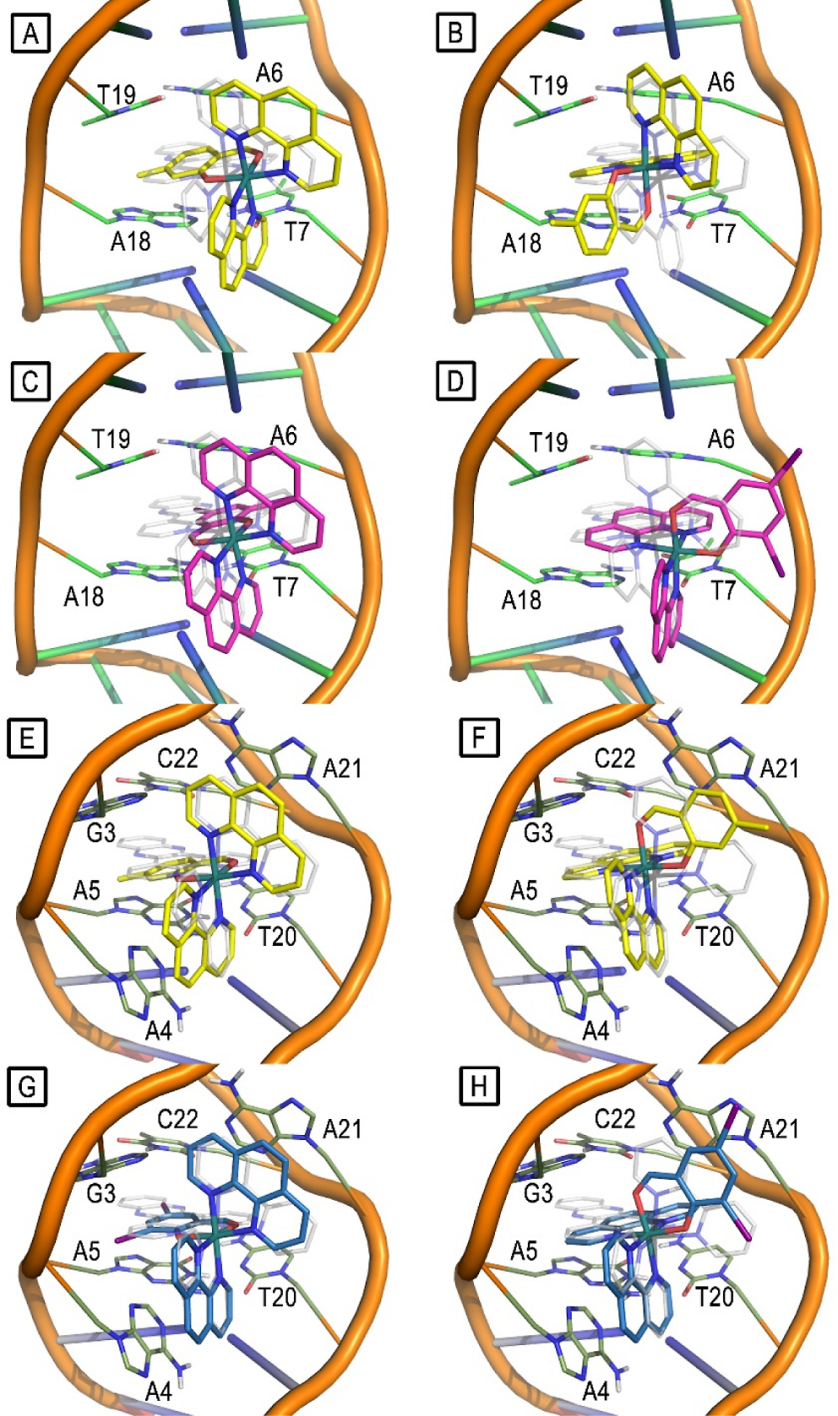
Salicyl-orientated (panel A) and aryl-orientated (panel B) docking poses of complex **10** (yellow sticks) bound to BP1 (green sticks). Salicyl-orientated (panel C) and aryl-orientated (panel D) docking poses of complex **7** (magenta sticks) bound to BP1 (green sticks). Salicyl-orientated (panel E) and aryl-orientated (panel F) docking poses of **10** (yellow sticks) bound to BP2 (olive sticks). Salicyl-orientated (panel G) and aryl-orientated (panel H) docking poses of complex **7** (magenta sticks) bound to BP2 (olive sticks). DNA phosphodiester backbone is represented in orange cartoons. **[Ru(bpy)**_**2**_**dppz]**^**2+**^ experimental position is depicted for reference, in every panel, as white transparent sticks.

In order to investigate the cytotoxicity mechanisms exerted by our compounds, molecular modeling studies were carried out by using as a model a 12-mer oligonucleotide sequence of DNA duplex cocrystallized with the Ru-based complex **[Ru(bpy)**_**2**_**dppz]**^**2+**^ (PDB: 4E1U). In this target structure, two main binding sites which can accommodate metal complexes are observed: a first binding pocket (BP1) formed by the well-matched DNA base pairs A6-T19 and T7-A18, and a binding pocket (BP2) formed by both the well-matched DNA base pairs G3-C22 and A5-T20 and the mismatched A4-A21 base pair. DNA mismatches constitute a well-known anticancer target as deficiencies in DNA mismatch repair have been associated with high rates of gene mutation and insurgence of several types of cancers.^69,70^ Compounds **10** and **7** were submitted to molecular docking simulations, and the obtained docking poses suggested that the two complexes are potentially able to interact with both BP1 and BP2, mimicking the crystallographic position of the complex **[Ru(bpy)**_**2**_**dppz]**^**2+**^.^51^ In particular, **10** and **7** assume two main binding modes: salicyl-orientation (a) and aryl-orientation (b), for both BP1 and BP2. At BP1, in binding mode (a), the salicylaldehyde group is partially intercalated within the bases A6-T19 and T7-A18 and the two phenanthroline rings create stacking interactions with the unpaired bases A9 and A21 (Figure 10, panels A, C); in binding mode (b), one of the two phenanthroline rings is intercalated between the bases A6-T19 and T7-A18, while the second phenanthroline ring and the salicylaldehyde moiety interact with bases A9 and A21 (Figure 10, panels B, D). At BP2, in binding mode, (a) the salicylaldehyde moiety is placed between the bases G3-C22 and A5-T20 and the phenanthroline rings interact with the mismatched bases A4-A21 (Figure 10, panels E, G); in binding mode (b), one of the two phenanthroline rings is intercalated between the bases G3-C22 and A5-T20, while the second phenanthroline ring and the salicylaldehyde ring create stacking interactions with the bases A4 and A21 (Figure 10, panels F, H).

**Figure 11:**
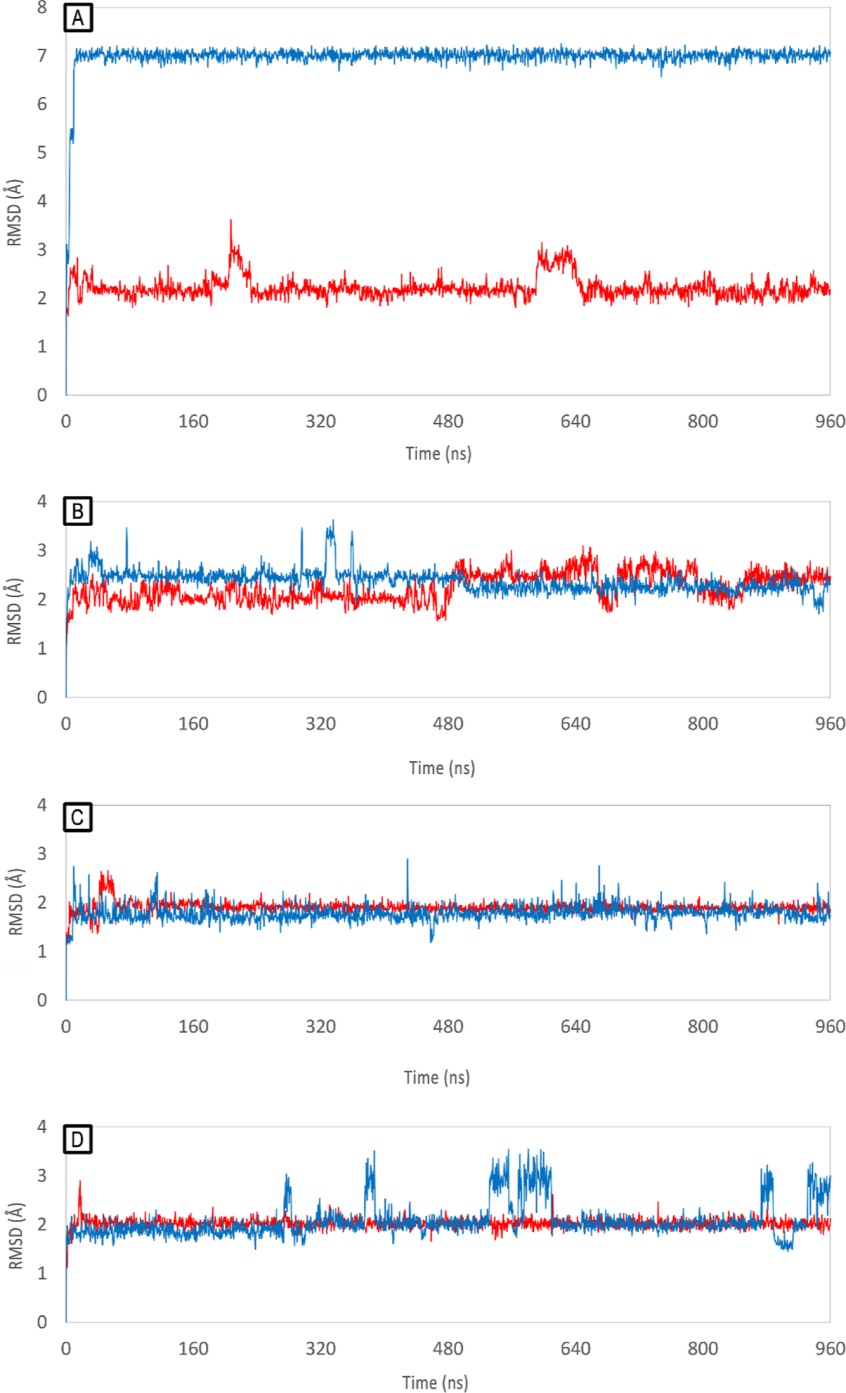
rmsd variations over 960 ns-long MD simulation of BP1-bound complex **10** (panel A); BP2-bound complex **10** (panel C); BP1-bound complex **7** (panel B) and BP2-bound complex **7** (panel D). Red lines represent salicyl-oriented binding mode and blue lines represent aryl-oriented binding mode.

Docking-predicted poses of **10** and **7** were used as starting points for molecular dynamics simulations, in order to investigate the dynamics of the two binding modes. Comparison of the compounds RMSD variations during the simulations (Figure 11) suggests that **10** and **7** preferentially interact at BP2 (Figure 11, panels C, D) rather than to BP1 (Figure 11, panels A, B), as the rmsd values calculated for the latter one are less stable during the simulation. The simulations show that the intercalation at BP1 occurs in less a buried region compared to BP2, since the steric hindrance of the phosphodiesteric backbone and the smaller size of BP1 hamper a deep intercalation of the metal complexes through the bases. This substantially influences the interaction of aryl-oriented poses, because the shape of the aryl group is less suitable for deep intercalation than the salicylaldehyde group. Indeed, the aryl-oriented poses of both complexes remain on the lower groove of the BP1 site, stabilized by stacking with the bases A9 and A21, without penetrating between the base pairs A6-T19 and T7-A18. On the other hand, the aryl-oriented complexes are able to intercalate deeper on the BP2 site but, as evident from minor rmsd fluctuations of the salicyl-orientation (Figure 11, red lines) versus aryl-orientation (Figure 11, blue lines), the salicyl-orientation appears the most favored. Further proofs of the selectivity of salicyl-orientation toward BP2 is obtained by checking the rmsd values in Figure 11, panel A. During the simulation of the aryl-oriented pose of complex **10** on BP1 (Figure 11, panel A, blue line) the complex escapes from the site and binds to BP2 by intercalation of its salicylaldehyde moiety. Based on these studies and in accordance with biological data reported above, we can hypothesize that the higher SI of compound **10** compared to compound **7** (i.e., 19.5 vs 2.2, respectively) is due to the higher propensity of the former to intercalate DNA BP2 which contains mismatched base pairs.

## Conclusions

In this work, we synthesized and characterized (elemental analysis and spectroscopic methods) a new panel of Ru(II) polypyridyl complexes, with general formula [Ru(phen)_2_(X-Sal)]BF_4,_ which were designed as anticancer agents. Single-crystal X-ray diffraction analysis performed on five of them showed that these complexes possess a six-coordinated structure arranged around the metal center and an overall distorted octahedral geometry.

All these complexes have been theoretically studied by quantum chemical calculations. The geometries of the complexes are in good agreement with the experimental structures determined by X-ray diffraction. All complexes display a similar electronic structure, with the HOMO, HOMO – 1, and HOMO – 2 located in the ruthenium center (the HOMO displays an additional significant participation of the salicyl ligand) and the LUMO, LUMO + 1, and LUMO + 2 located over the phenanthroline ligands. The TD-DFT calculations have been used to assign the bands observed in the absorption and emission spectra. The absorption bands correspond to ^1^MLCT from the ruthenium center to the phenanthroline ligands. The emission bands correspond to ^3^MLCT/^3^LLCT because the HOMO is involved in these transitions, and this orbital is spread over the ruthenium center and the salicyl ligand.

In vitro biological assessments carried out on two leukemic cell lines, i.e., the CCRF-CEM cell line and its multidrug-resistant counterpart CEM/ADR500, highlighted that these complexes are endowed with remarkable cytotoxicity (IC_50_ values in the submicromolar/low-micromolar range) and intriguing SI [up to 19.5 for the complex [Ru(phen)_2_(4-Me-Sal)]BF_4_ (**10**)) evaluated on PBMC]. The simultaneous presence of the metal ion and the salicyl auxiliary ligand turned out to be essential for the antiproliferative activity since both the ligands selected as controls and the complex lacking such auxiliary ligand, i.e., **Ru(phen)**_**2**_**Cl**_**2**_, were inactive in the screening test at 10 μM. Moreover, the substitution pattern at the salicyl ligand also plays an important role on the biological outcome as the complexes bearing 4-EDG and 3,5-dihalogen substitution displayed superior antiproliferative activity. The in silico studies, consistently with what observed in the biological assessments, suggest that the salicylaldehyde moiety might drive the binding of the complexes toward duplex DNA mismatched base pairs highlighting its essential role in the anticancer activity.

## Supporting Information Available

^1^The Supporting Information is available free of charge at https://pubs.acs.org/doi/10.1021/acs.inorgchem.3c03414.

H NMR spectra of all complexes, 2D-NMR spectra of complexes **2**, **3**, **10**, and **11**, electronic structures of complexes **1** and **3**–**12**, cyclic voltammograms of complexes **3**, **10**, and **11**, fluorescence spectra of complexes **2**, **3**, **10**, and **11**, and data of theoretical calculations (PDF)
