# Supplementary material for: Investigating the Cytotoxicity of Ru(II) Polypyridyl Complexes by Changing the Electronic Structure of Salicylaldehyde Ligands
Source: Inorg Chem. 2023 Dec 29;63(2):1083–101. doi: 10.1021/acs.inorgchem.3c03414 (PMC10792608; doi:10.1021/acs.inorgchem.3c03414)
Supplement: Supplementary file 1 — ic3c03414_si_001.pdf [file ic3c03414_si_001.pdf]

SUPPORTING INFORMATION

Investigating the cytotoxicity of Ru(II) polypyridyl complexes by changing the electronic structure of salicylaldehyde ligands

Maryam Taghizadeh Shool[a], Hadi Amiri Rudbari[a,*], José V. Cuevas-Vicario[b,*], Andrea Rodríguez-Rubio[b], Claudio Stagno[c], Nunzio Iraci[c], Thomas Efferth[d], Ejlal A. Omer[d], Tanja Schirmeister[e], Olivier Blacque[f], Nakisa Moini[g], Esmail Sheibani[a], Nicola Micale[c,*]

[a] *Department of Chemistry, University of Isfahan, Isfahan 81746-73441, Iran.*

[b] *Departamento de Química, Facultad de Ciencias, Universidad de Burgos, Plaza Misael Bañuelos s/n, 09001, Burgos, Spain.*

[c] *Department of Chemical, Biological, Pharmaceutical and Environmental Sciences, University of Messina, Viale Ferdinando Stagno D'Alcontres 31, I-98166 Messina, Italy.*

[d] *Department of Pharmaceutical Biology, Institute of Pharmaceutical and Biomedical Sciences, Johannes Gutenberg University, Staudinger Weg 5, 55128 Mainz, Germany.*

[e] *Department of Medicinal Chemistry, Institute of Pharmaceutical and Biomedical Sciences, Johannes Gutenberg University, Staudinger Weg 5, 55128 Mainz, Germany.*

[f] *Department of Chemistry, University of Zurich, Winterthurerstrasse 190, CH-8057, Zurich, Switzerland.*

[g] *Department of Chemistry, Faculty Chemistry Alzahra University, P.O. Box 1993891176, Vanak Tehran, Iran.*

* Corresponding authors.

E-mail addresses: h.a.rudbari@sci.ui.ac.ir, hamiri1358@gmail.com (H. Amiri Rudbari), nmicale@unime.it (N. Micale), jvcv@ubu.es (J. V. Cuevas-Vicario)

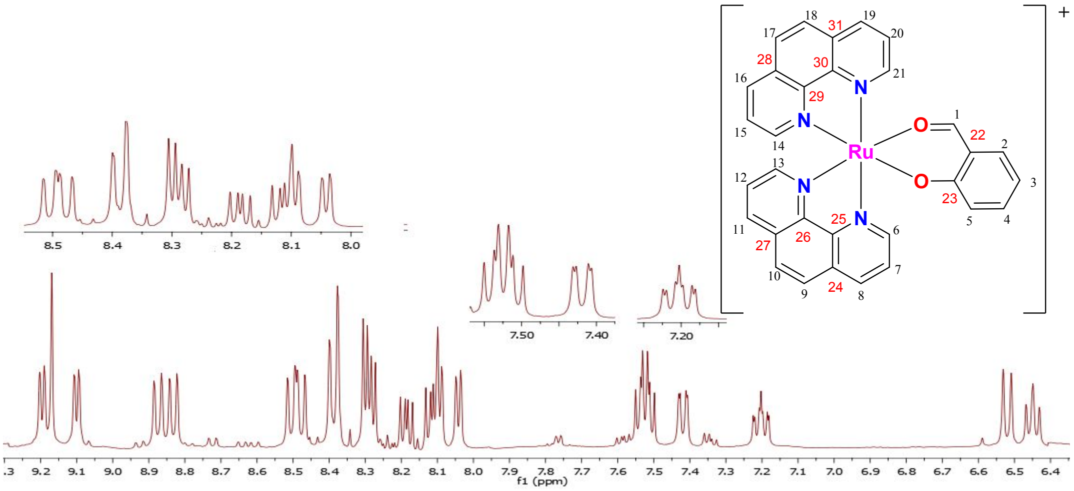

Fig. S1 ^{1}H-NMR spectra of complex **1** in DMSO.

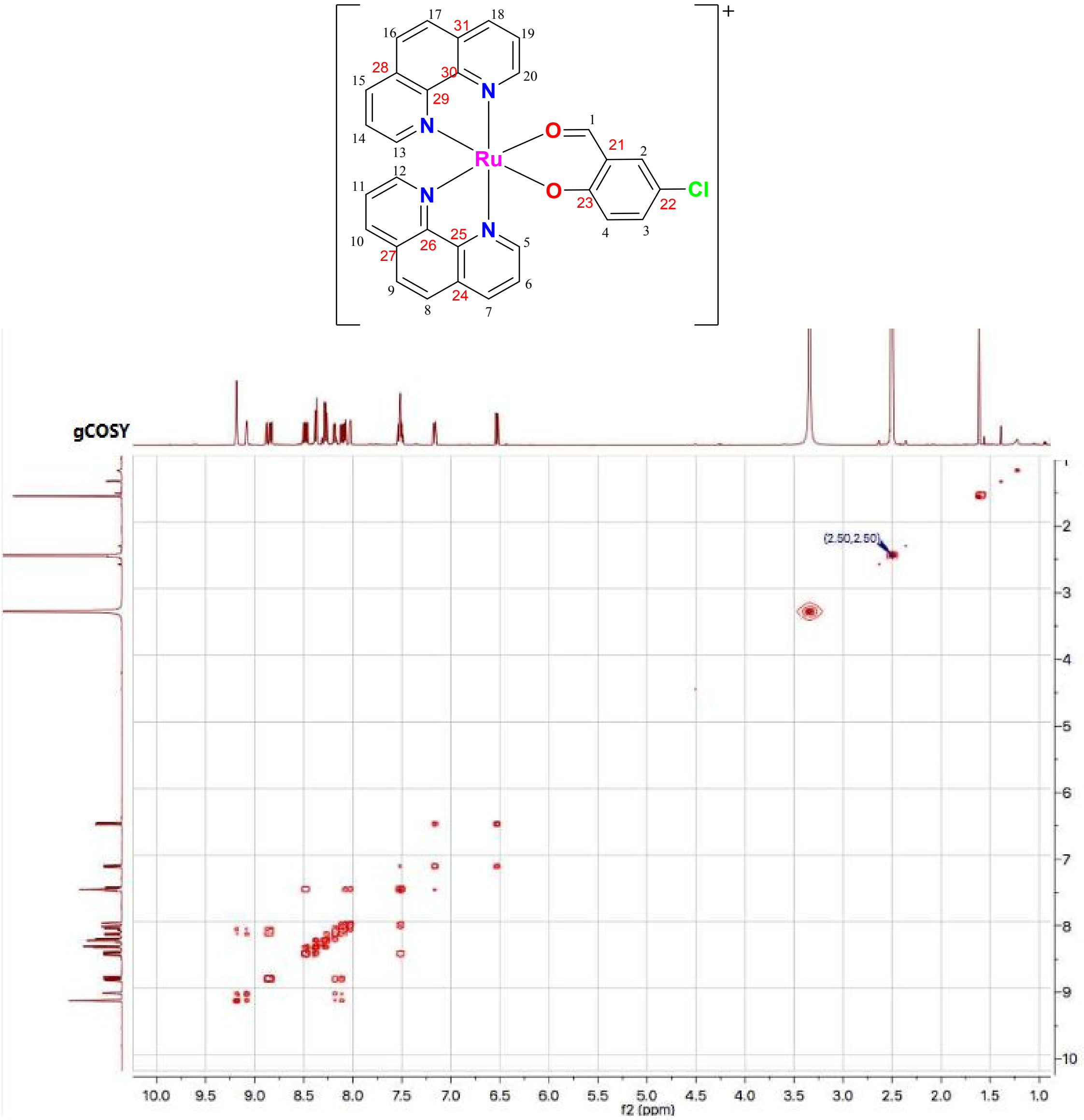

gHMBC

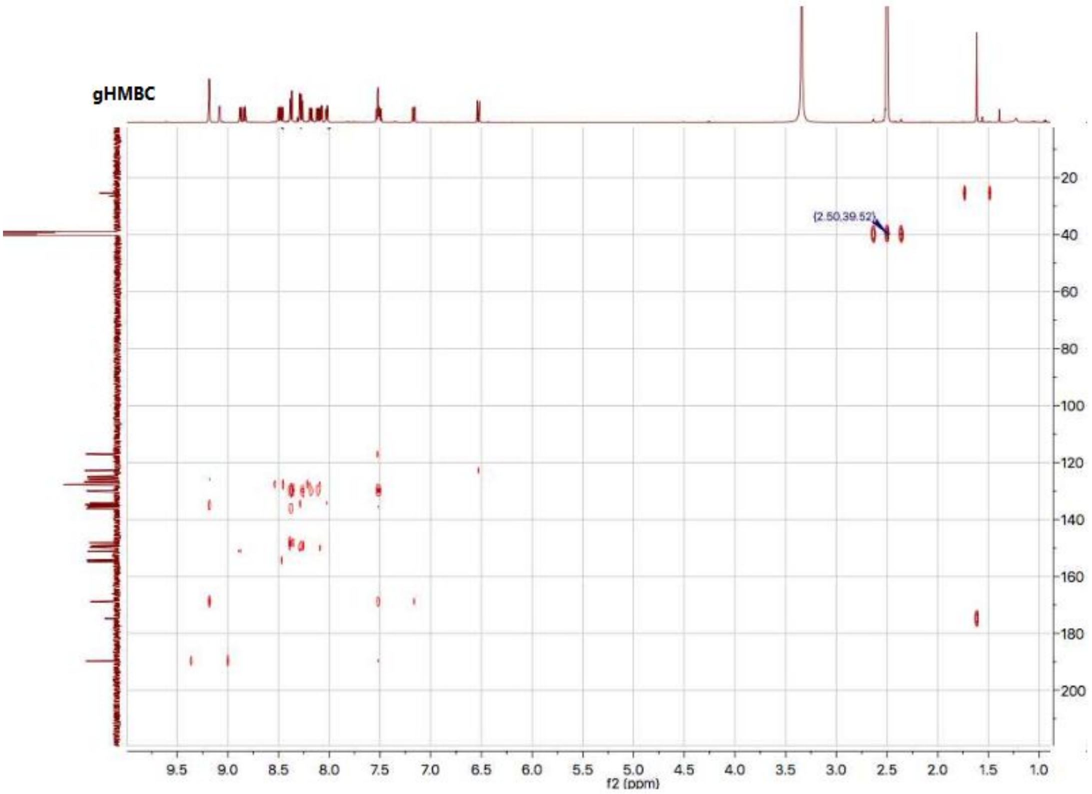

gHSQC

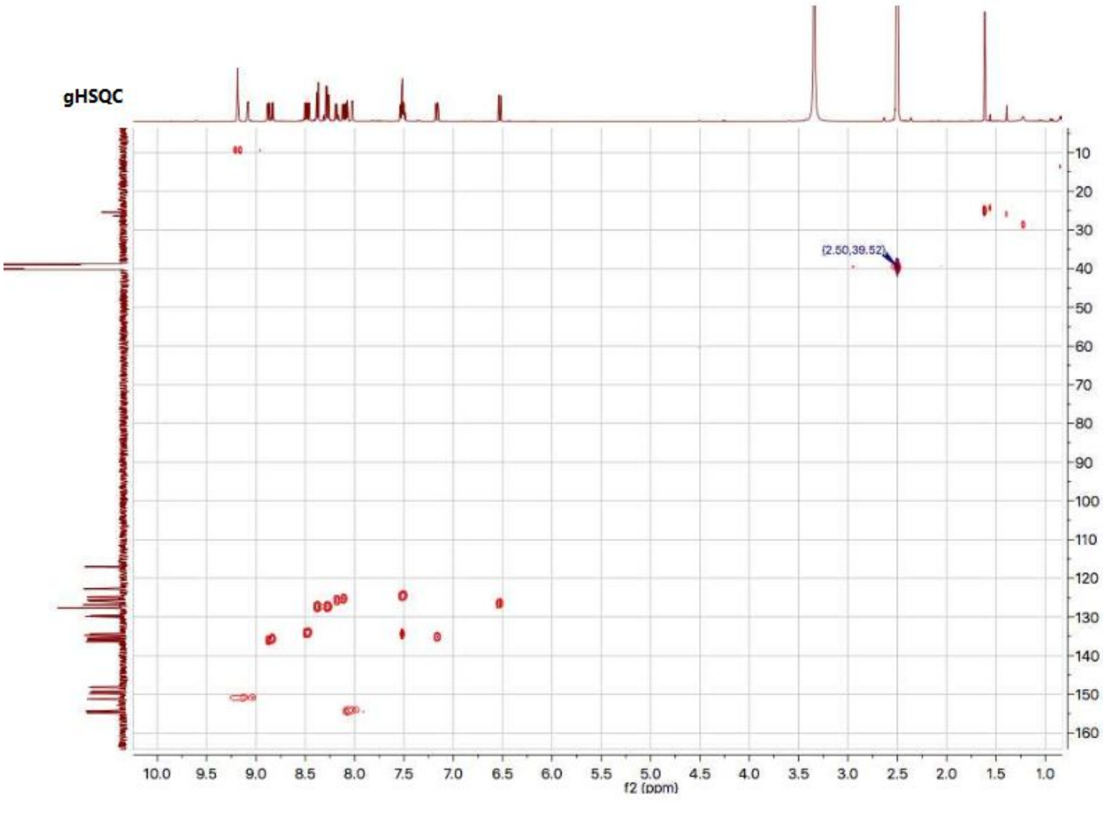

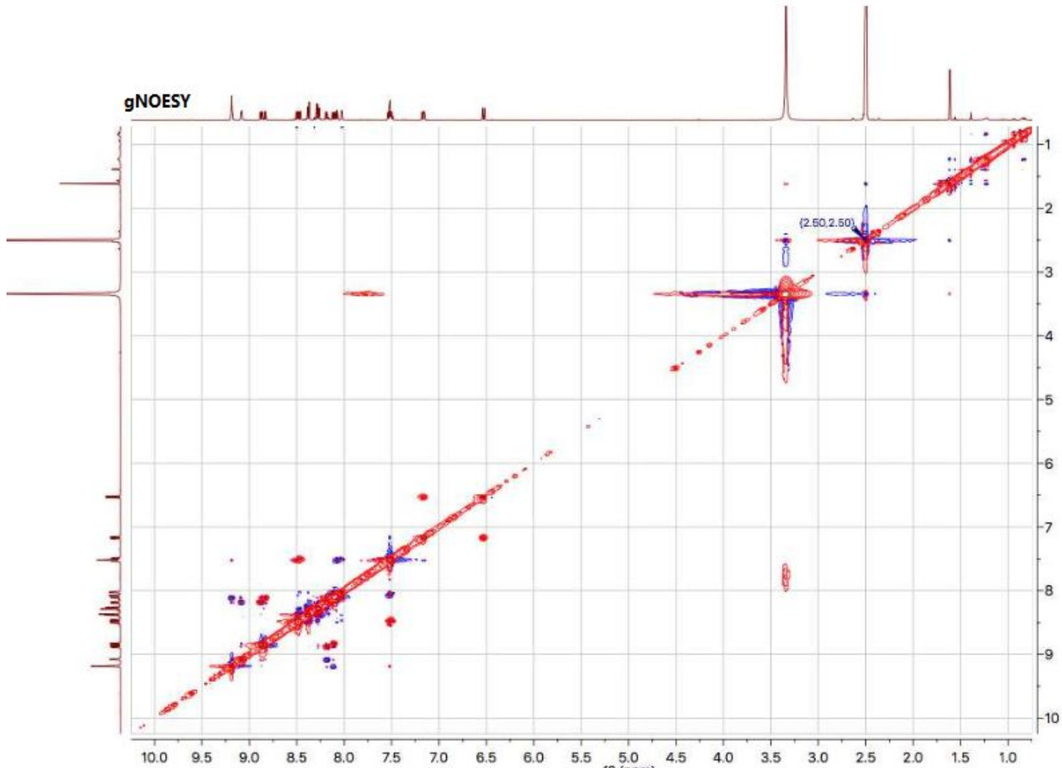

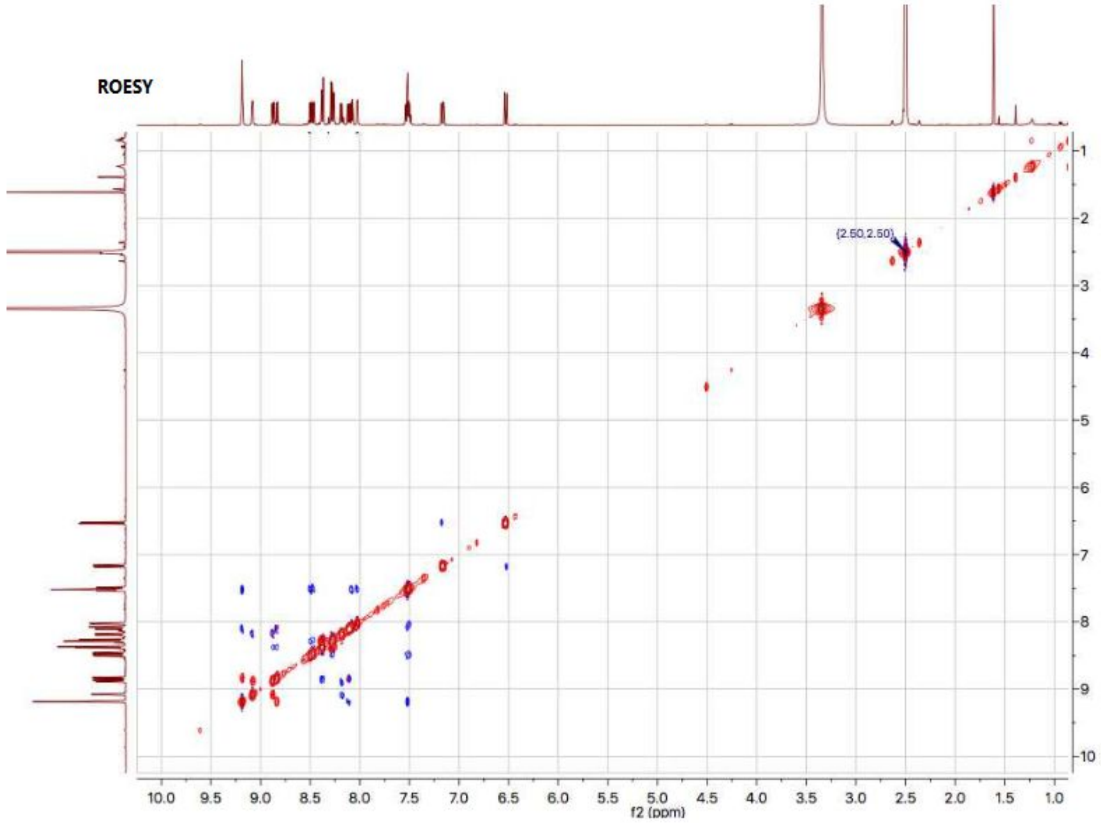

Fig. S2 ^{1}H-NMR and 2D NMR spectra of complex **2** in DMSO.

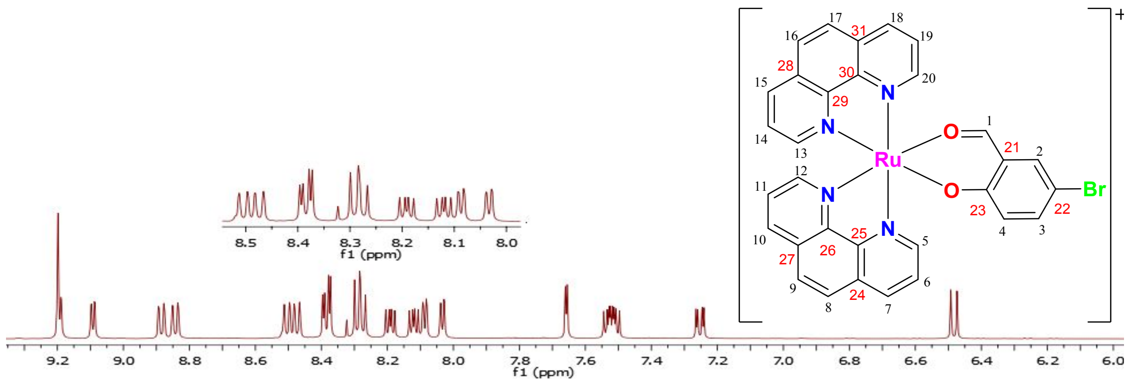
Ru
Br
N
O
f1 (ppm)

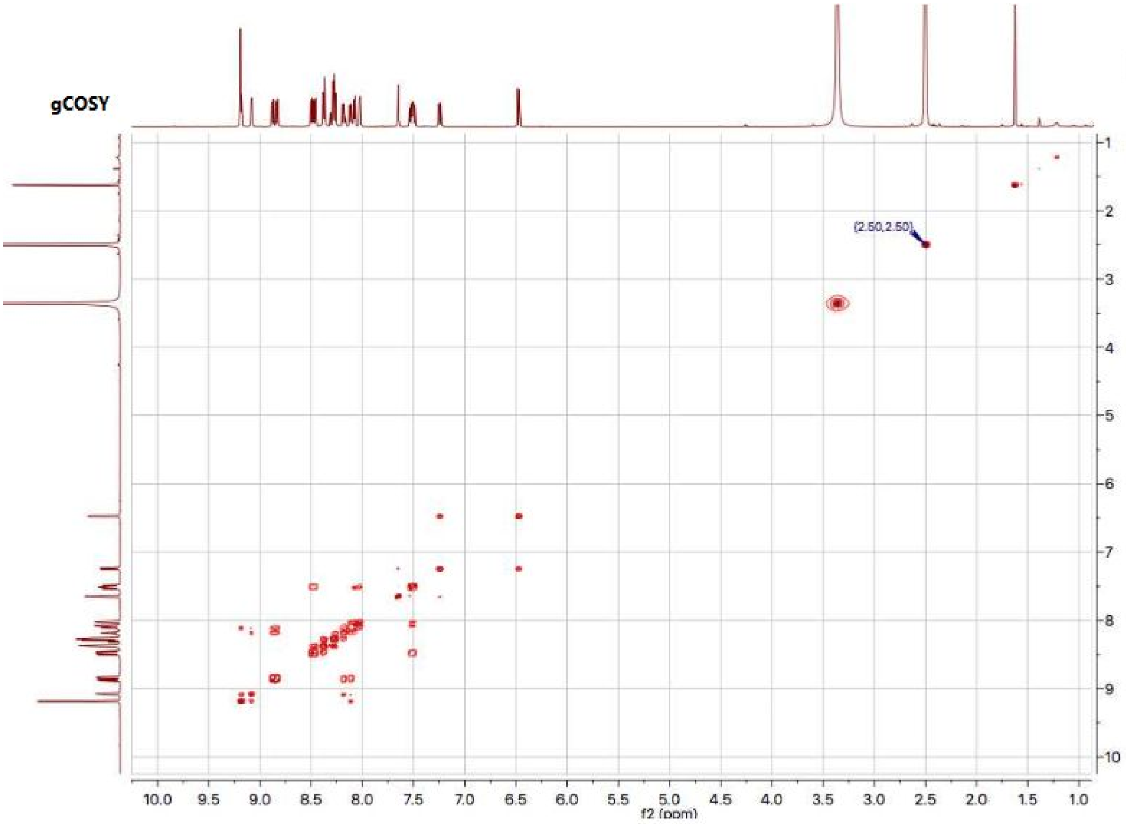
gCOSY
(2.50,2.50)
f2 (ppm)

gNOESY

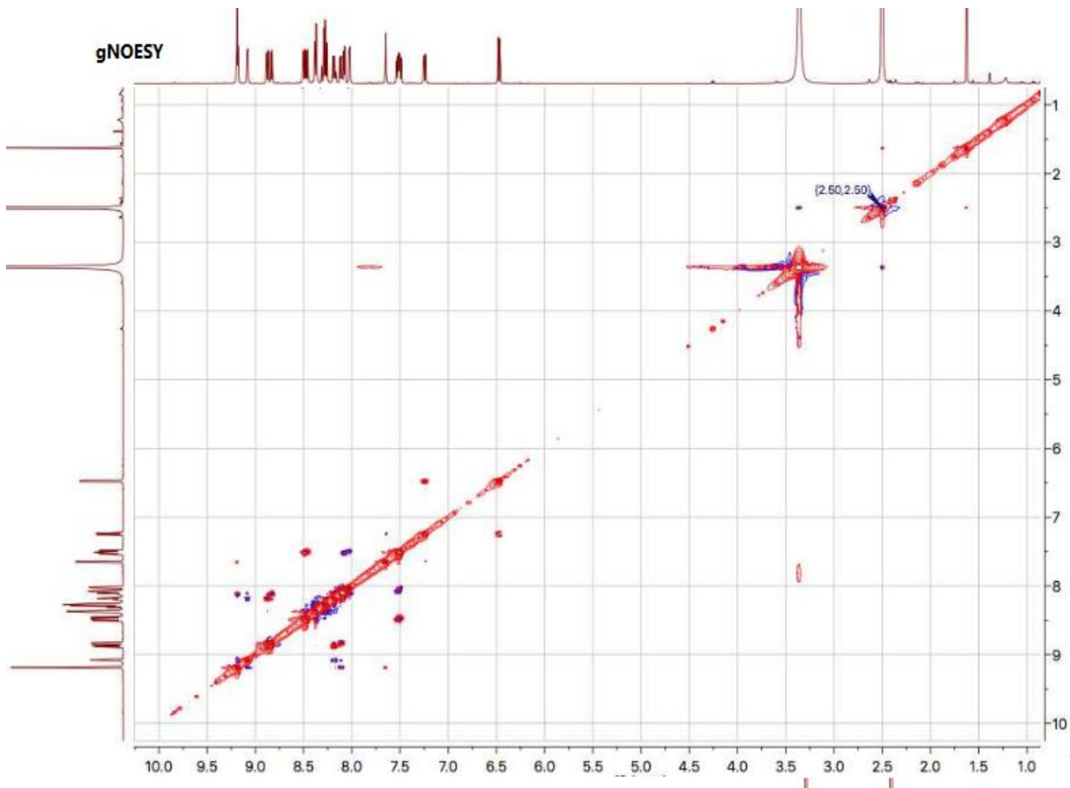

gHSQC

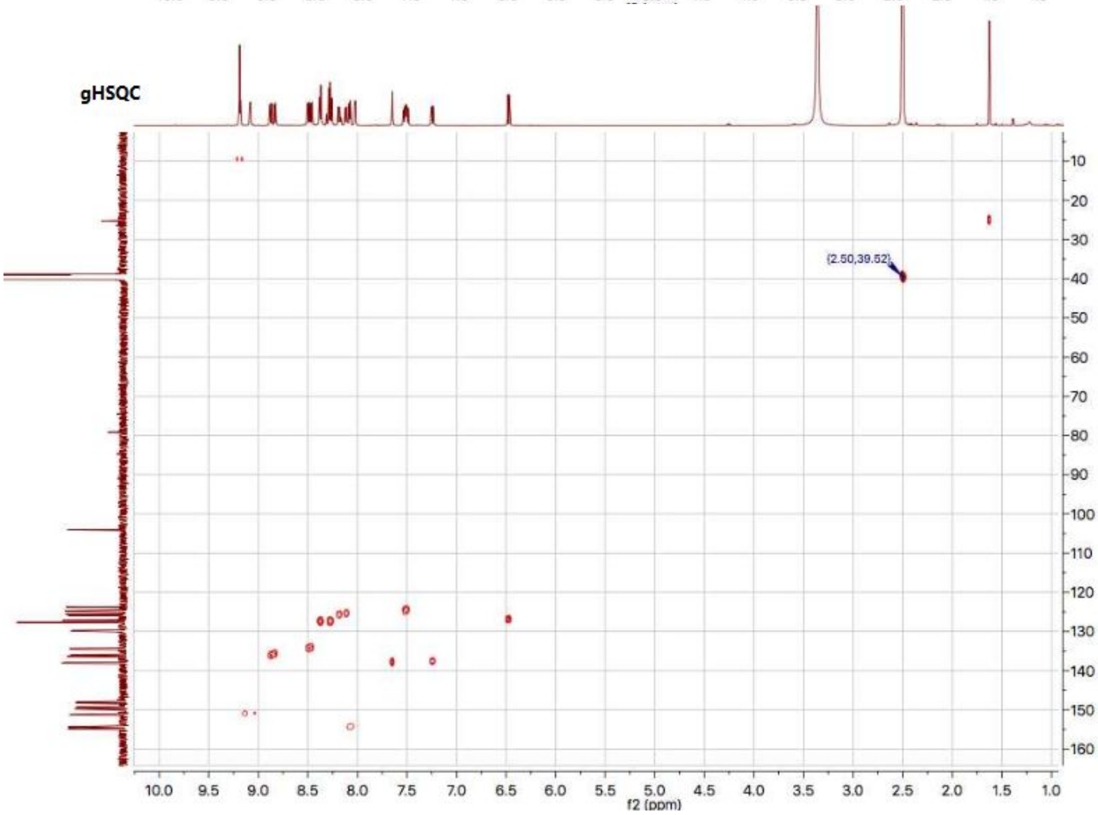

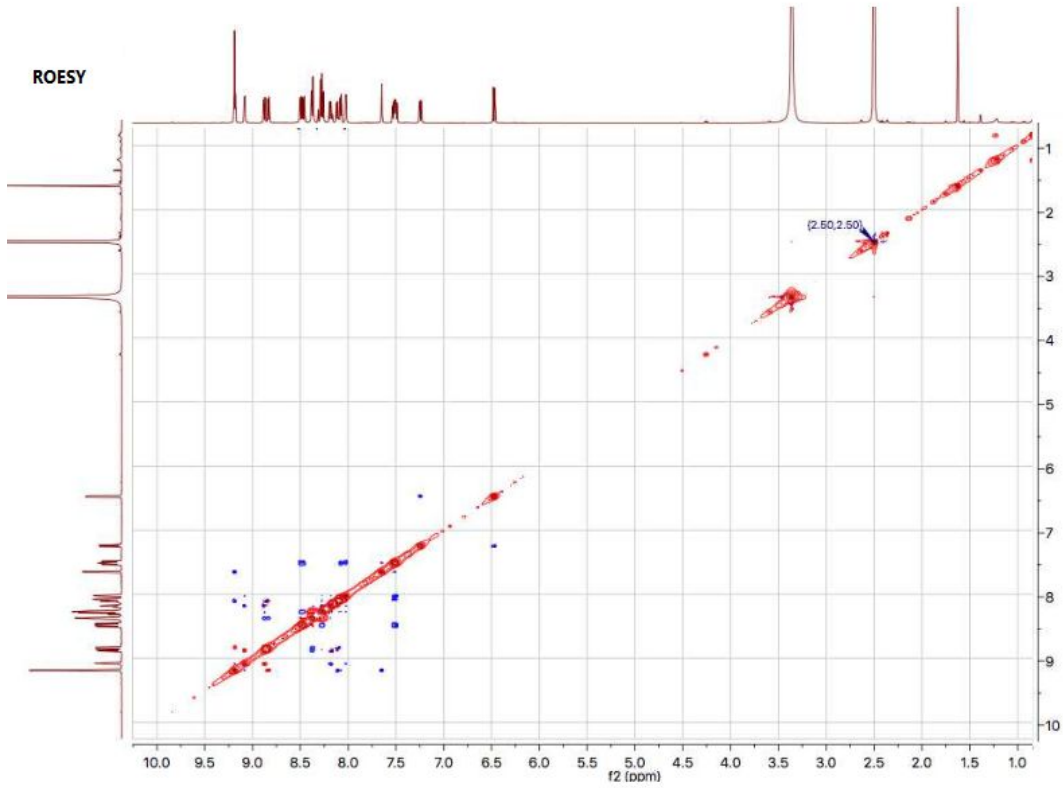

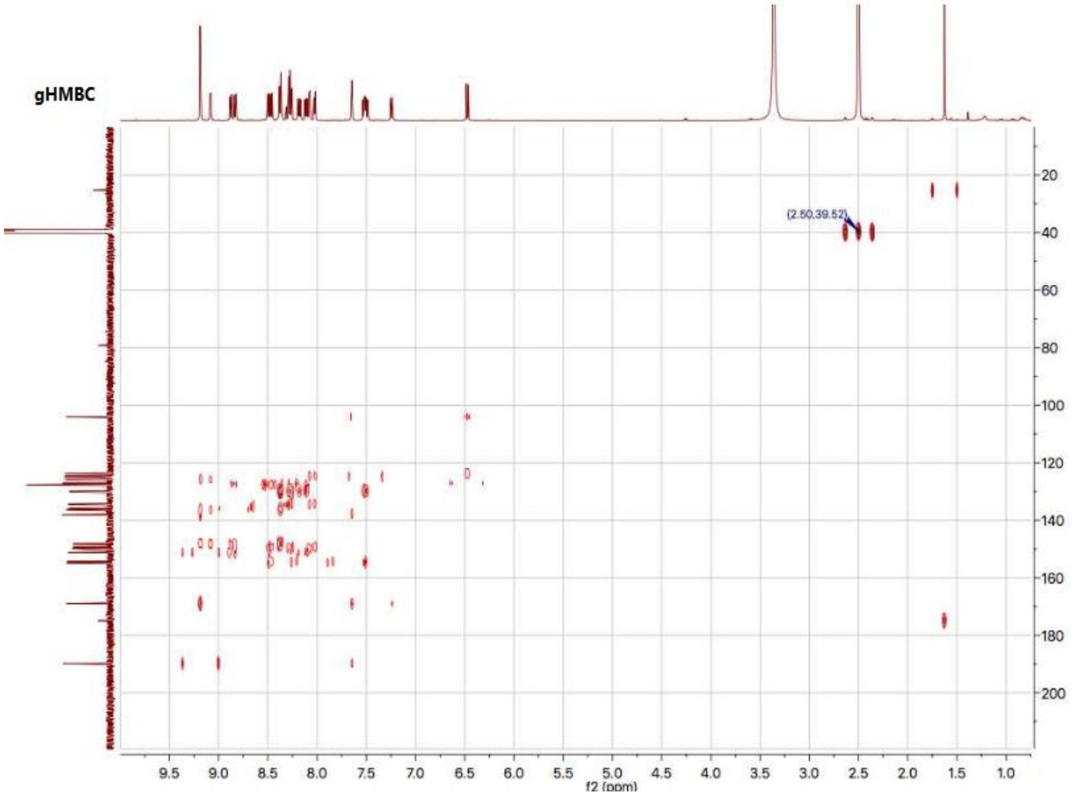

Fig. S3 ^{1}H-NMR and 2D-NMR spectra of complex **3** in DMSO.

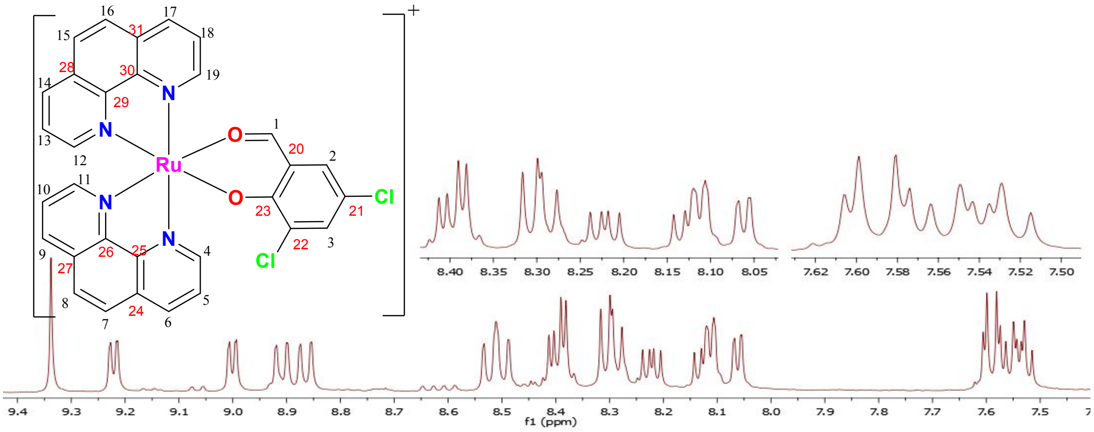

Fig. S4 ^{1}H-NMR spectra of complex **4** in DMSO.

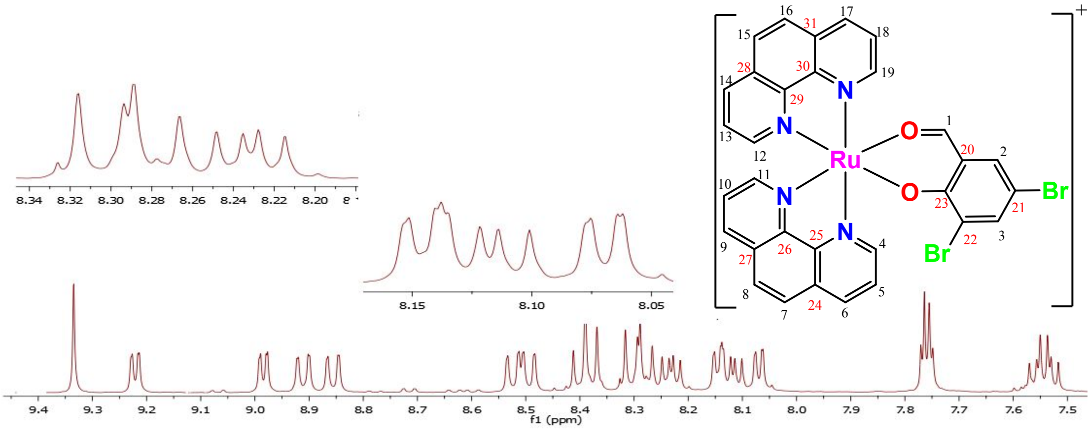

Fig. S5 ^{1}H-NMR spectra of complex **5** in DMSO.

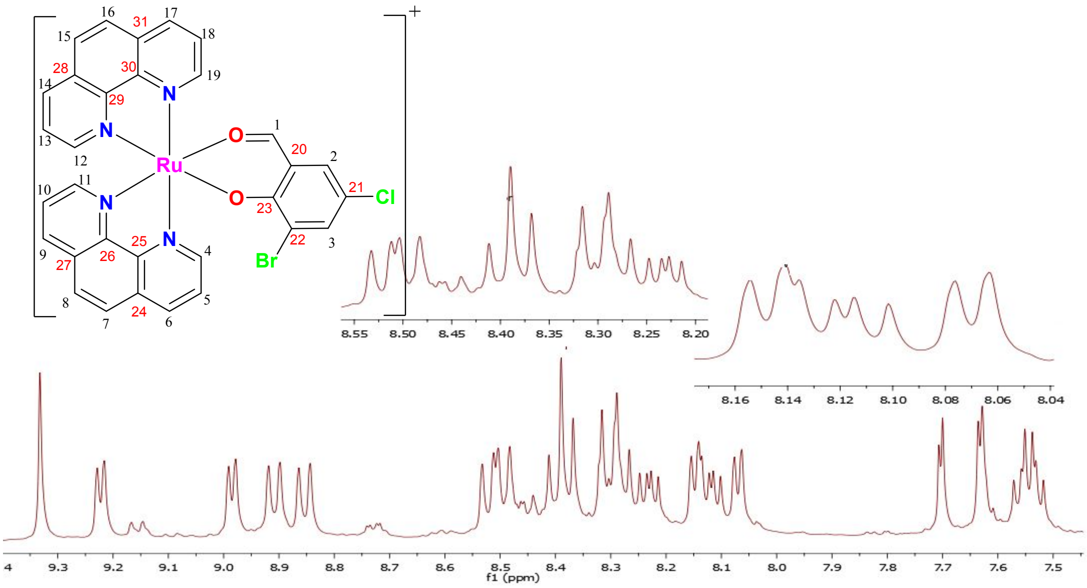

Fig. S6 ^{1}H-NMR spectra of complex **6** in DMSO.

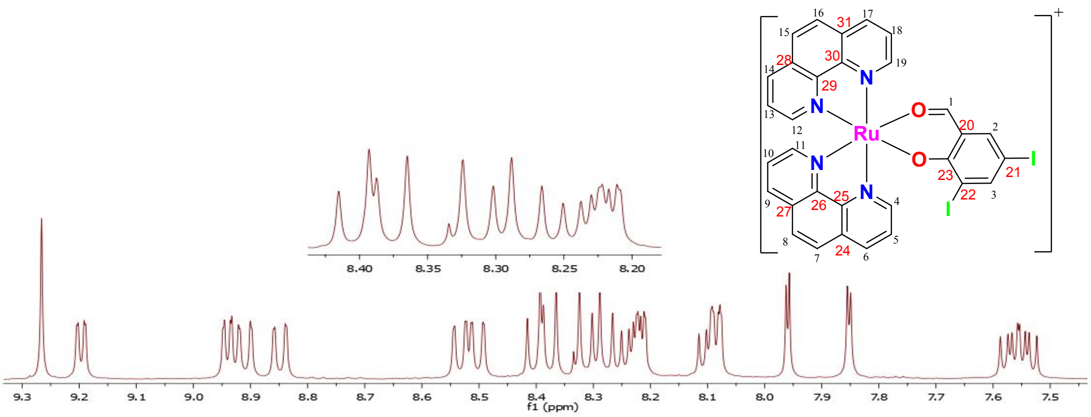

Fig. S7 ^{1}H-NMR spectra of complex **7** in DMSO.

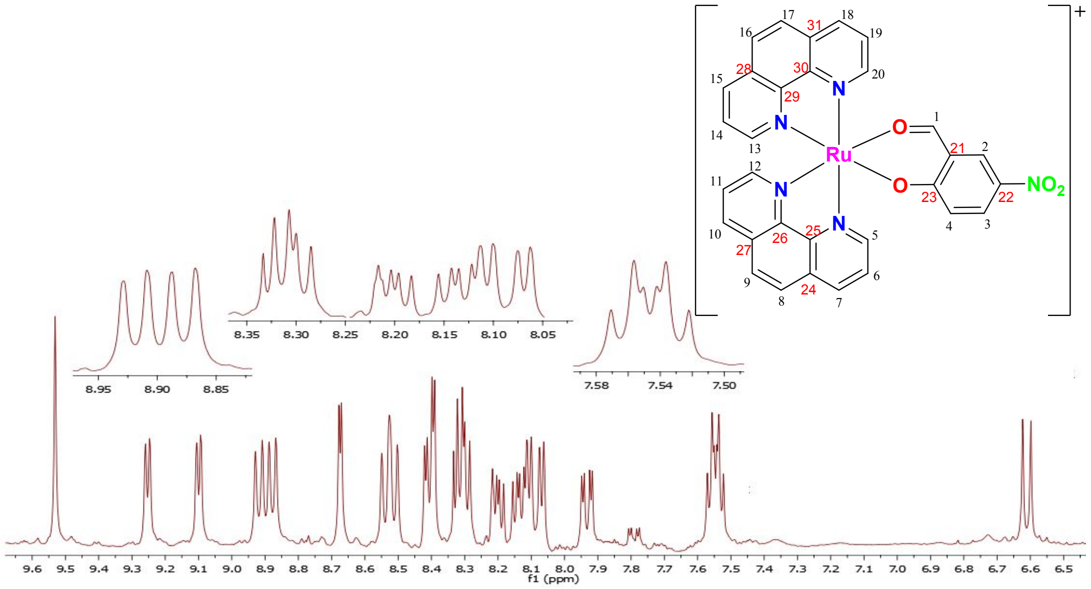

Fig. S8 ^{1}H-NMR spectra of complex **8** in DMSO.

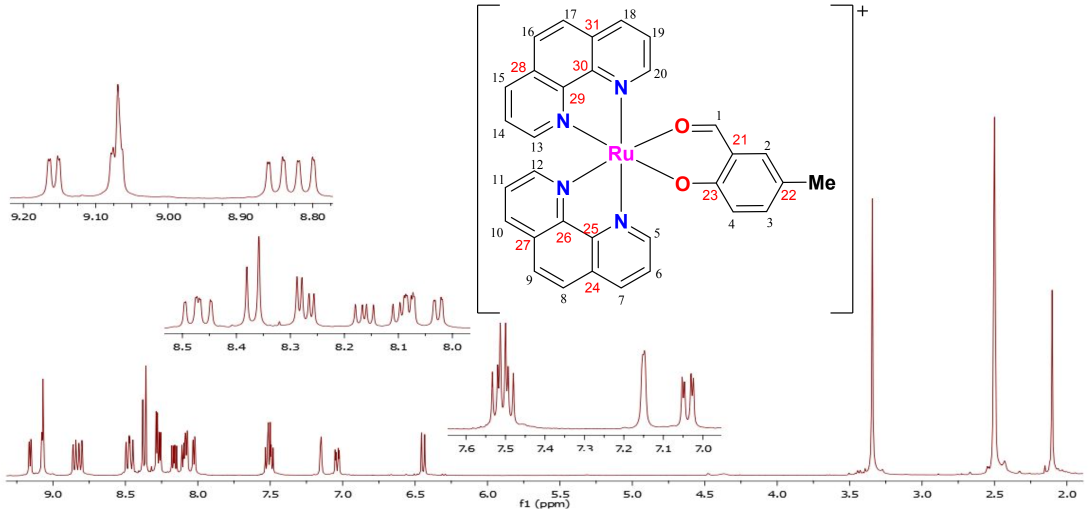

Fig. S9 ^{1}H-NMR spectra of complex **9** in DMSO.

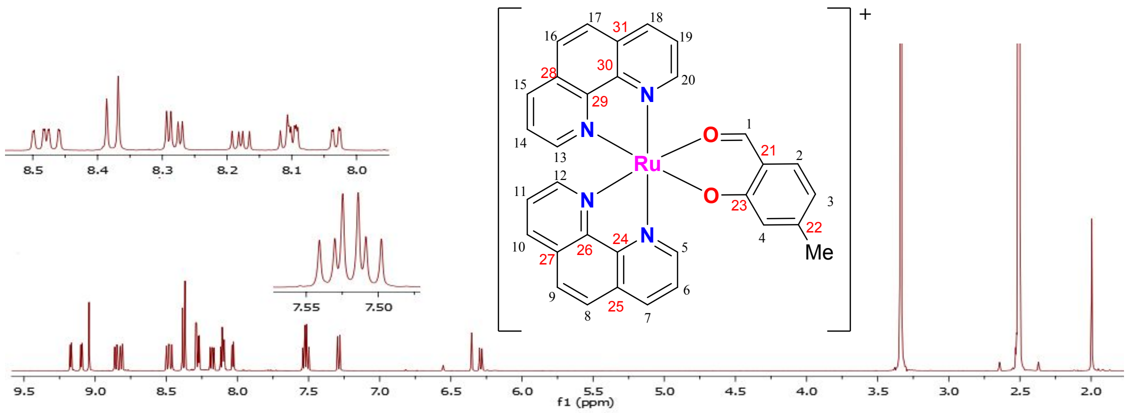
+
Ru
N
O
Me
f1 (ppm)

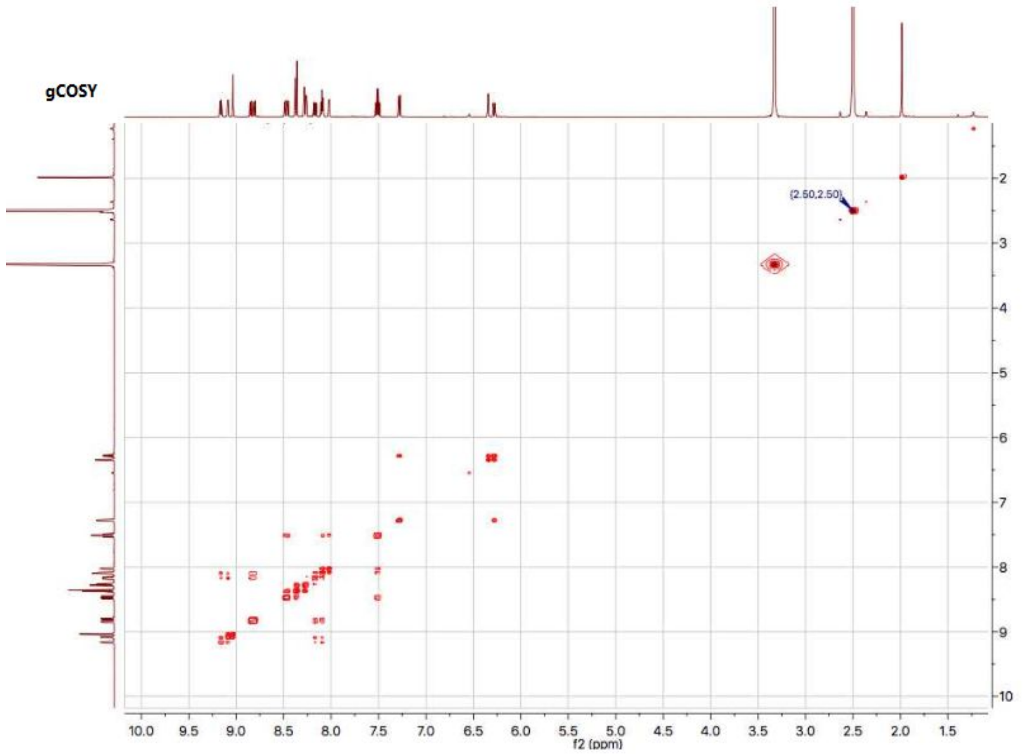
gCOSY
(2.50,2.50)
f2 (ppm)

gHMBC

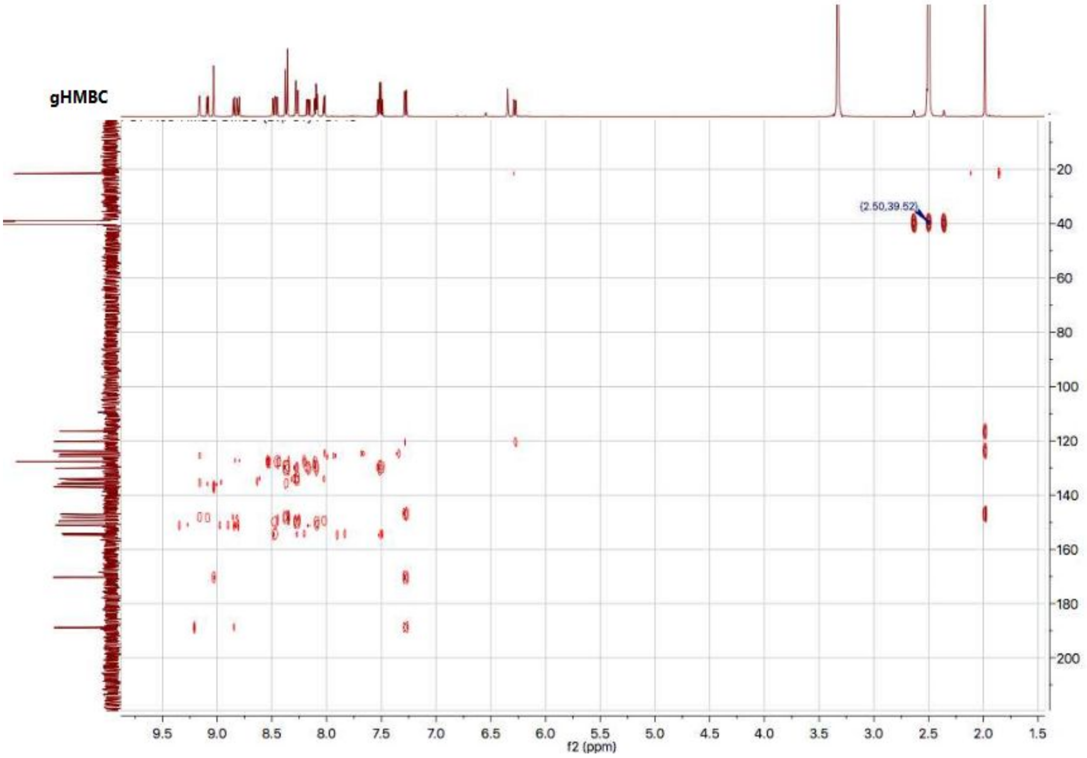

gHSQC

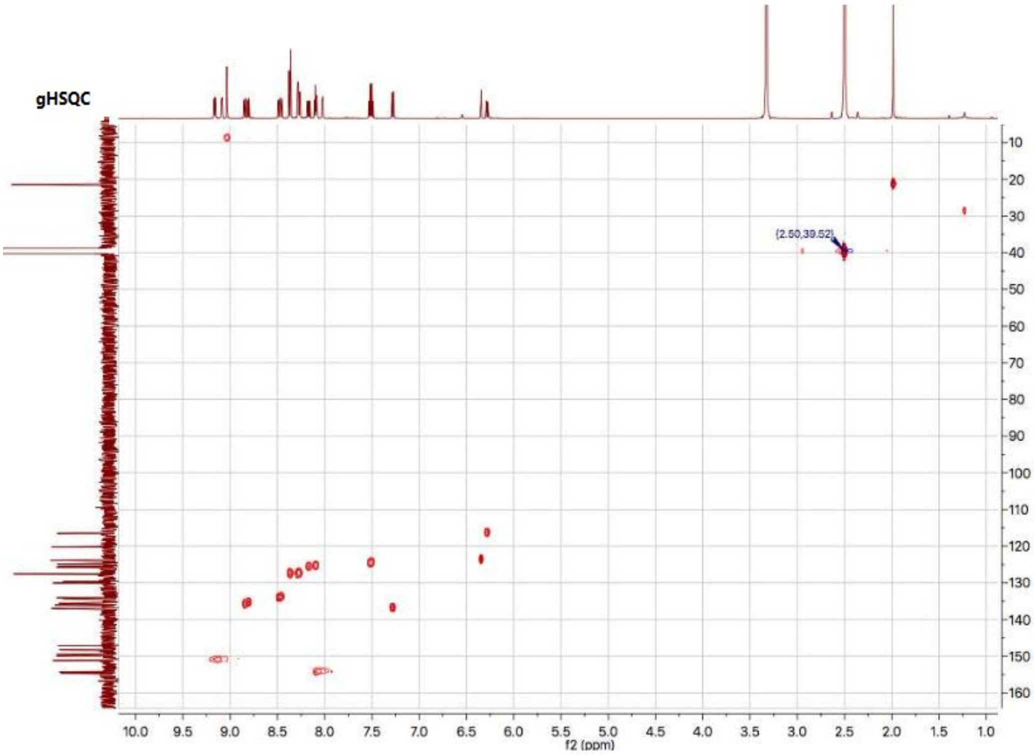

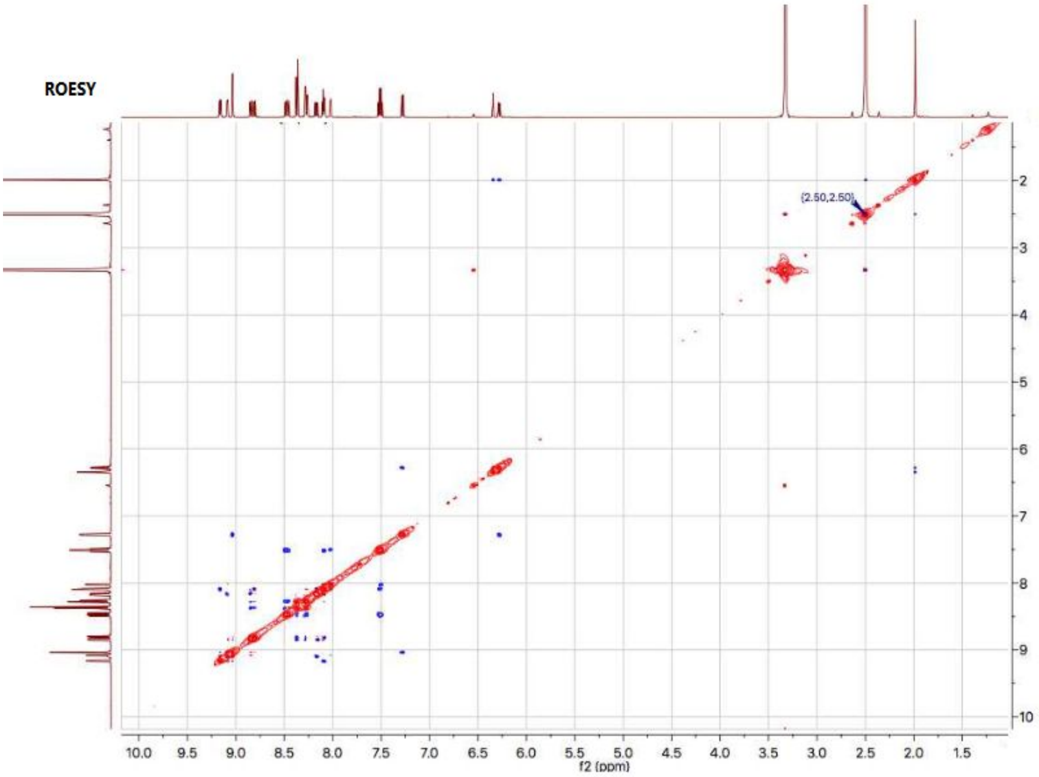

Fig. S10 ^{1}H-NMR and 2D-NMR spectra of complex **10** in DMSO.

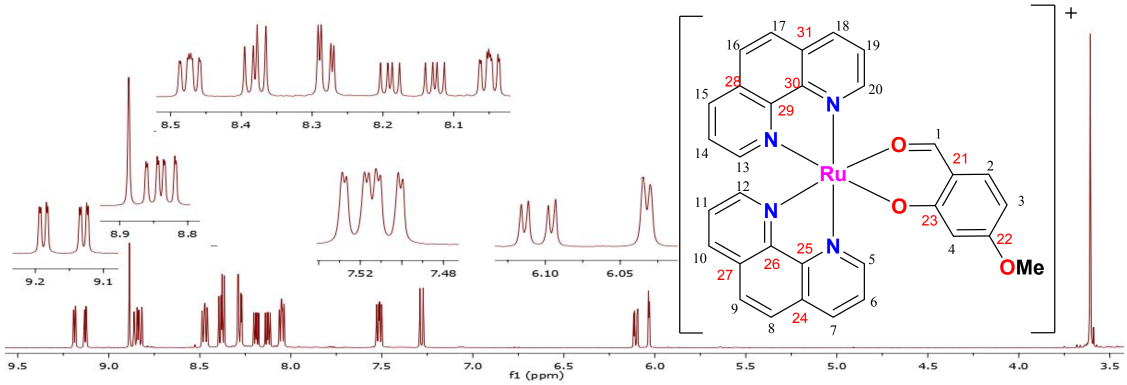

gCOSY

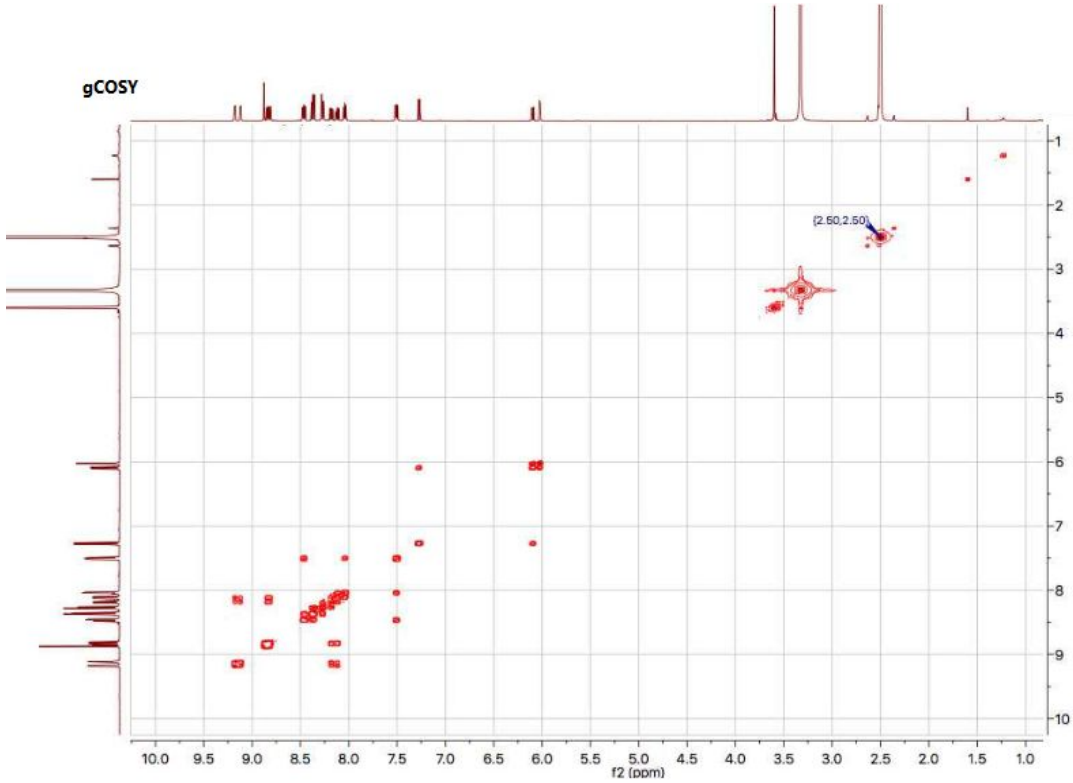

gHMBC

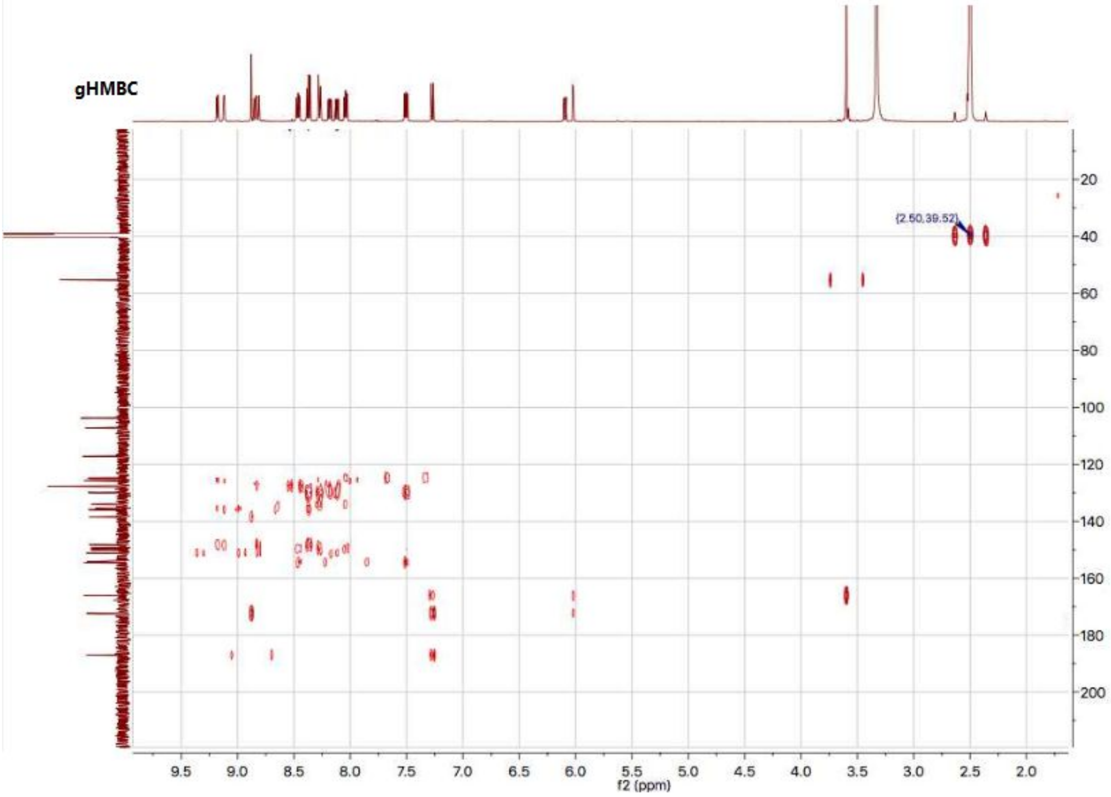

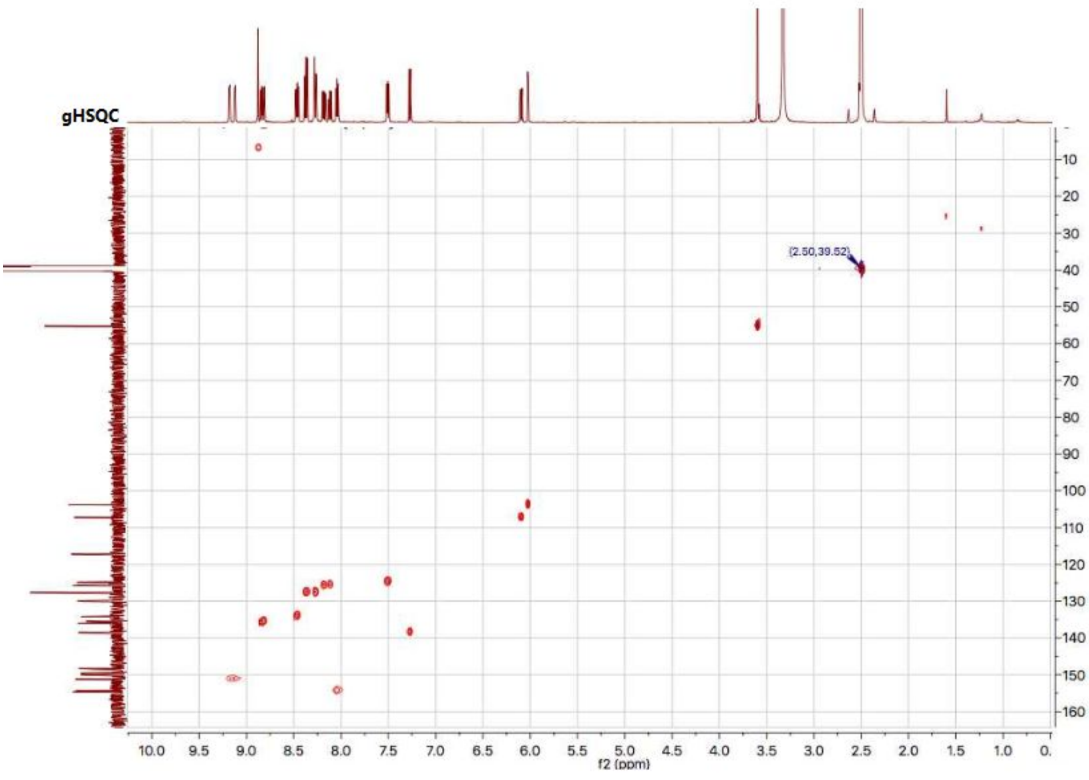

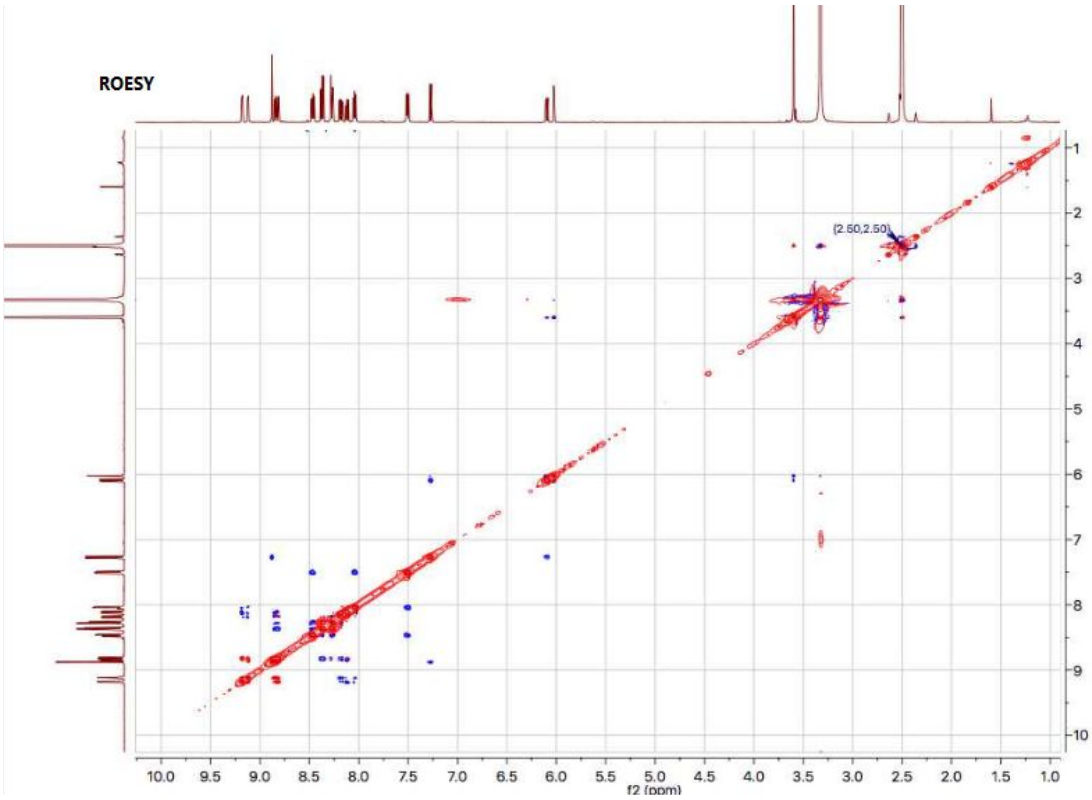

Fig. S11 ^{1}H-NMR and 2D-NMR spectra of complex **11** in DMSO.

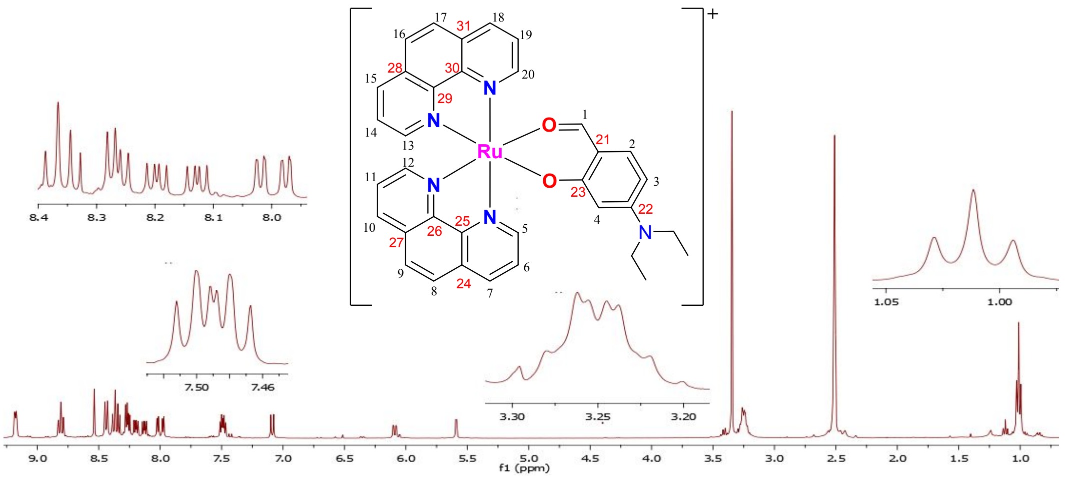

Fig. S12 ^{1}H-NMR spectra of complex **12** in DMSO.

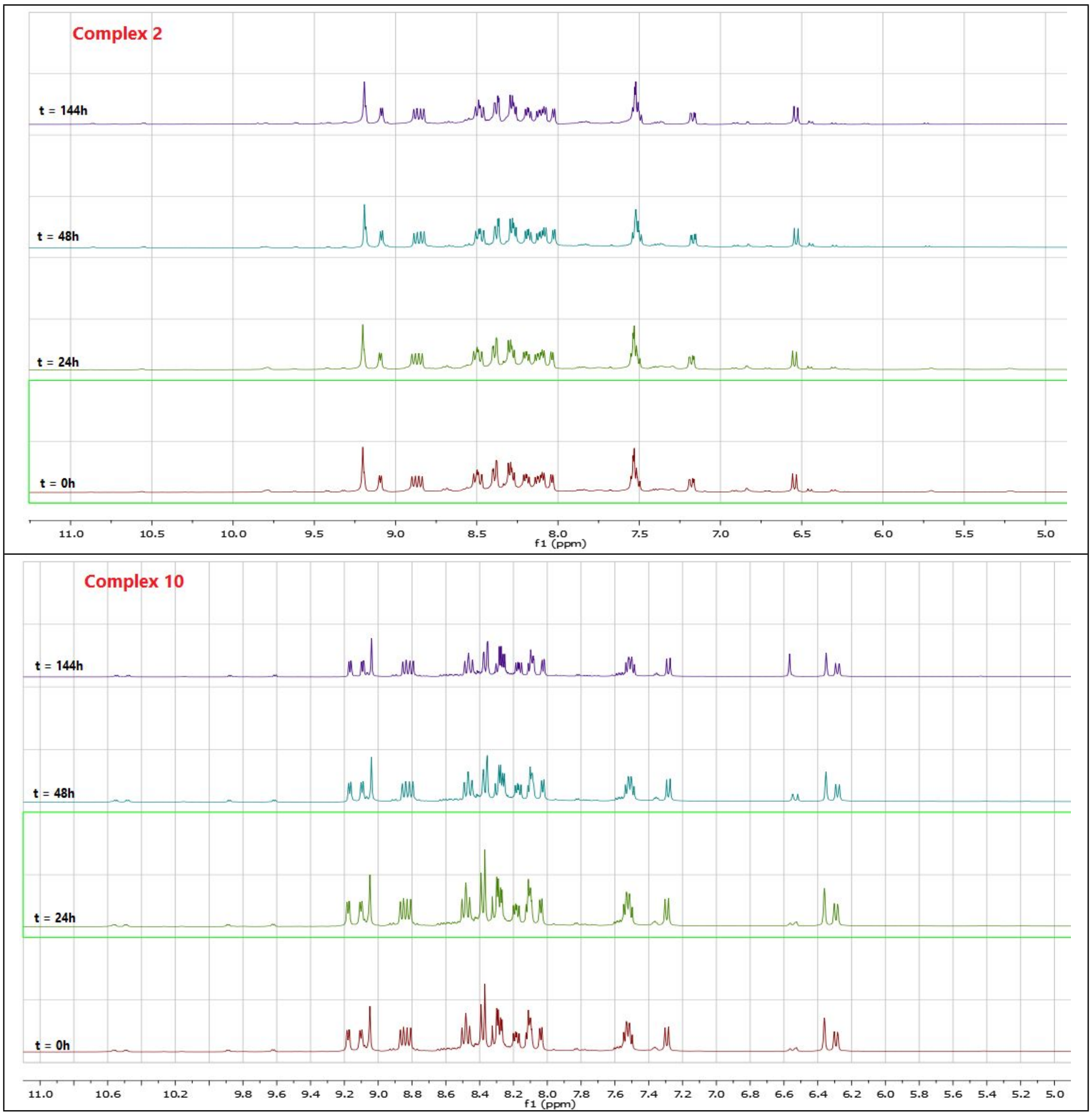

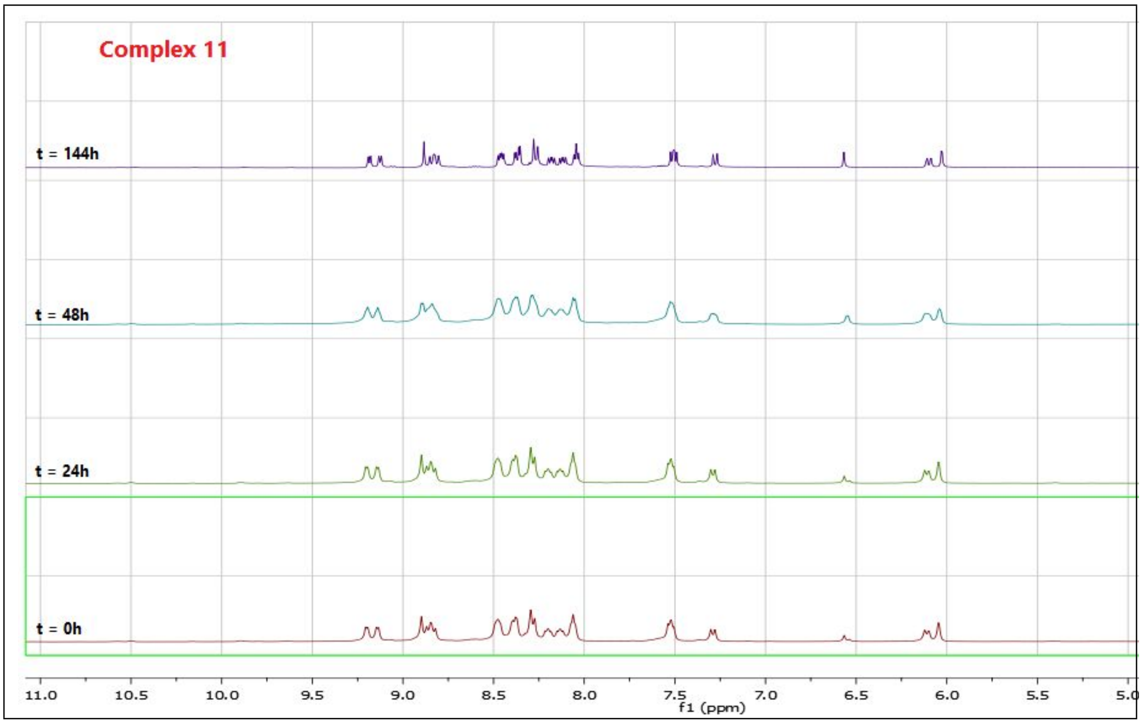

Fig. S13 ^{1}H NMR spectra for complexes 2, 10 and 11 in DMSO-d6 recorded at different incubation times under environmental scattered light.

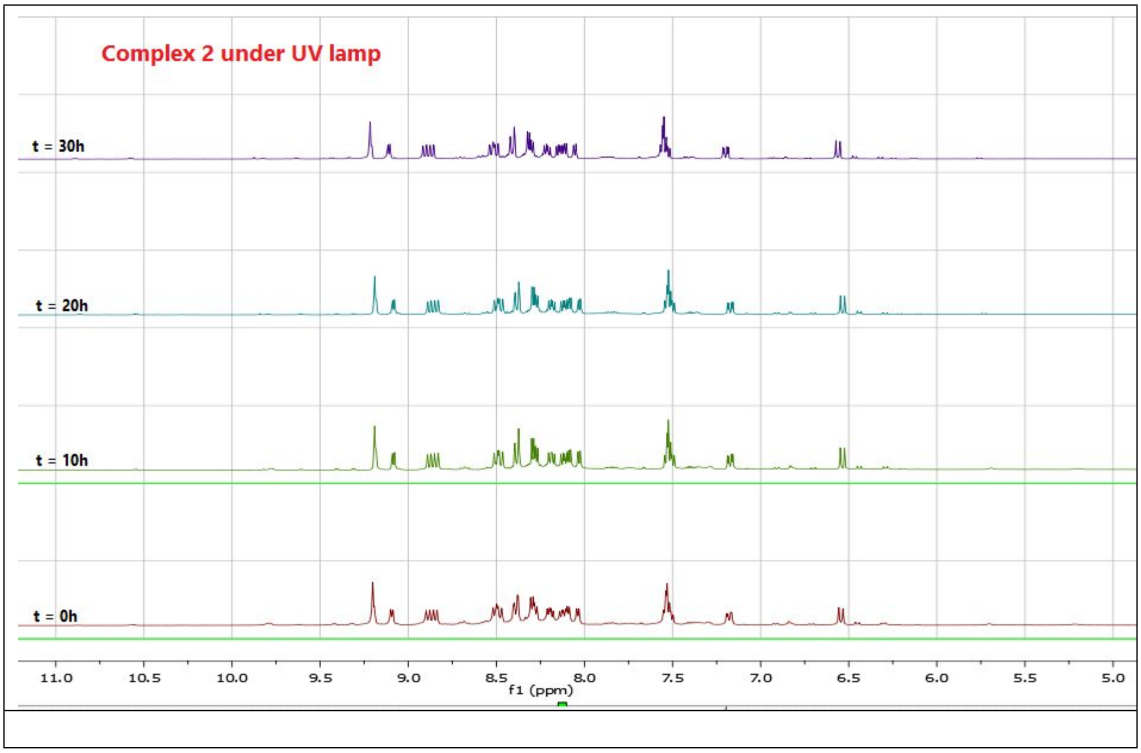

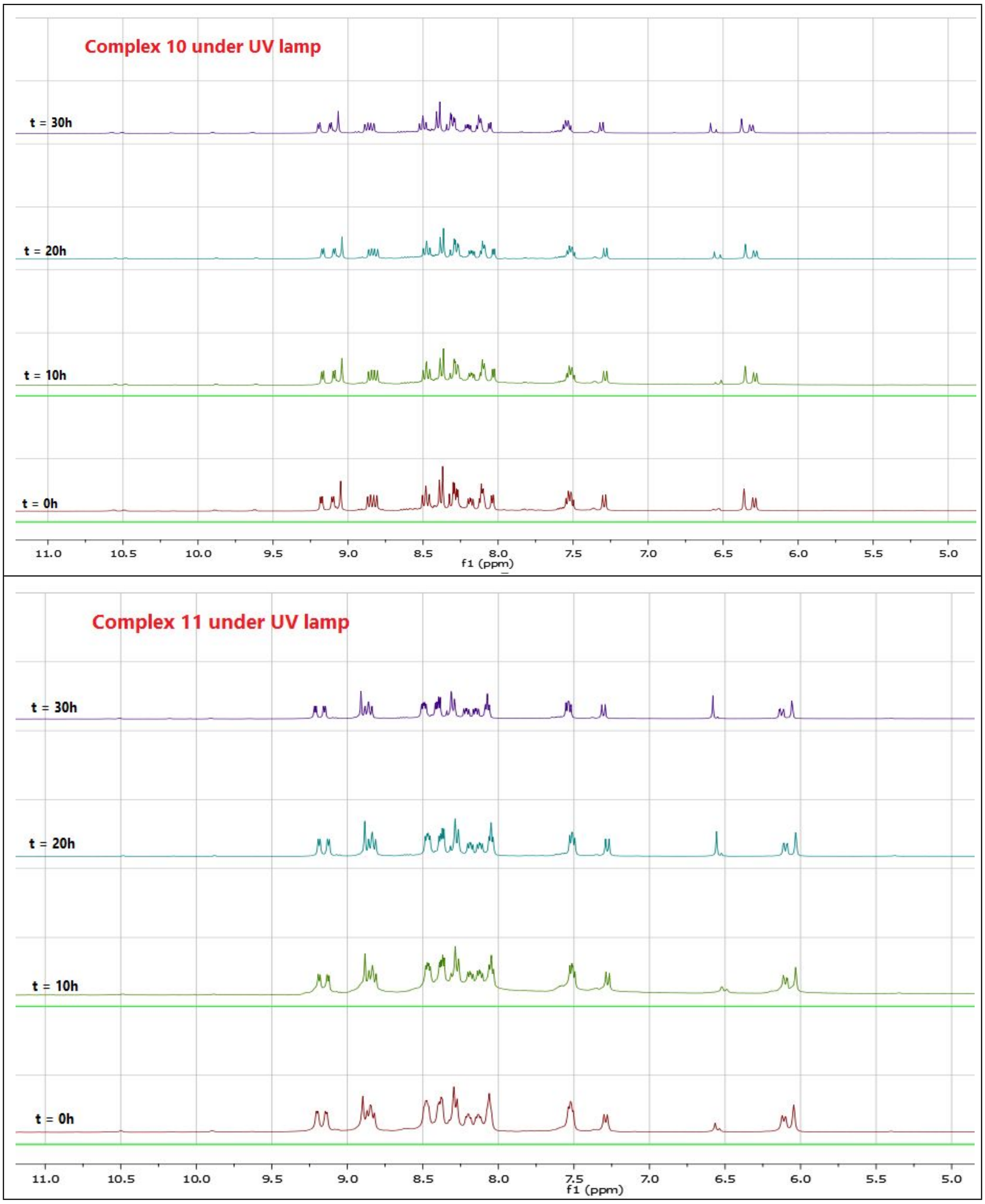

Fig. S14 ^{1}H NMR spectra for the complexes 2, 10 and 11 in DMSO-d6 recorded under UV lamp (λ = 254 nm) at different incubation times.

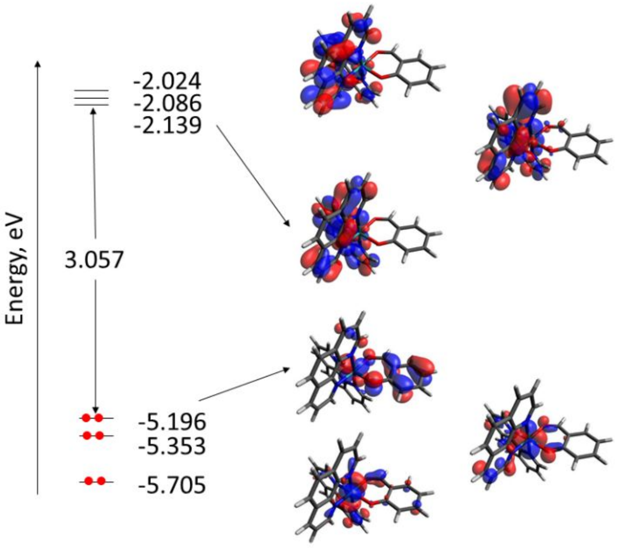

Fig. S15 Energy levels and isosurface contour plots (0.03 a.u.) for cation of compound **1.**

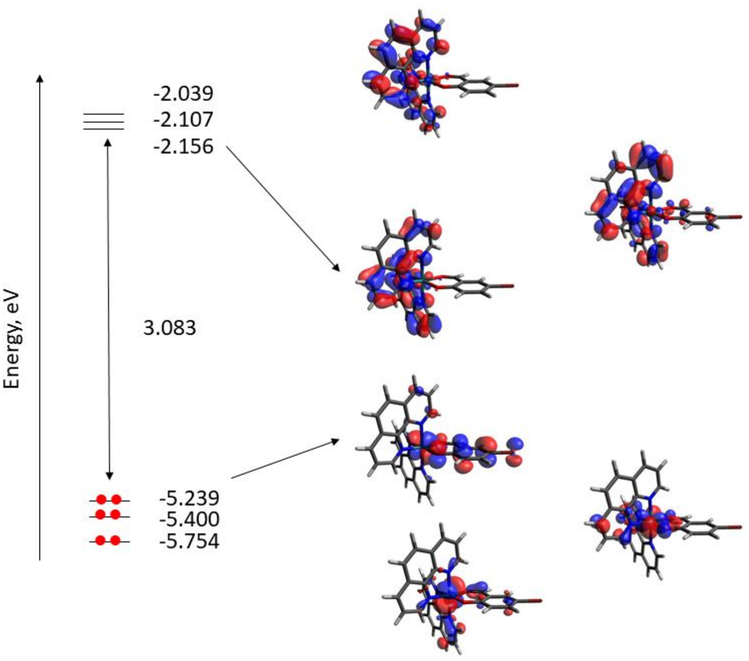

Fig. S16 Energy levels and isosurface contour plots (0.03 a.u.) for cation of compound **3.**

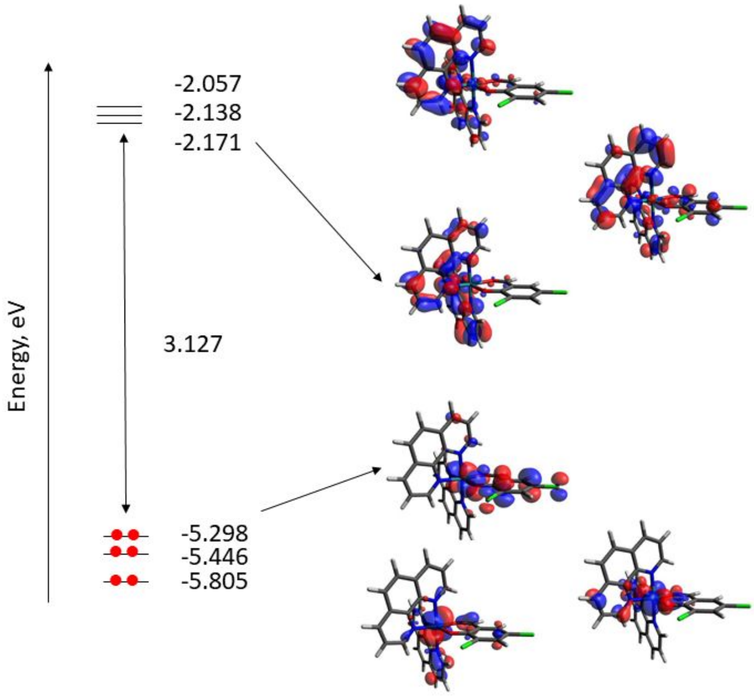

Fig. S17 Energy levels and isosurface contour plots (0.03 a.u.) for cation of compound **4**.

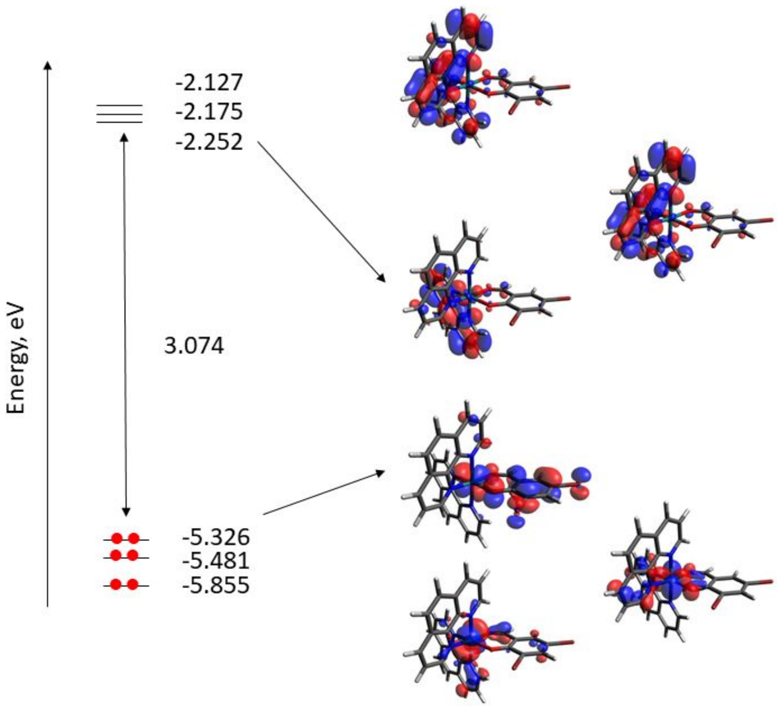

Fig. S18 Energy levels and isosurface contour plots (0.03 a.u.) for cation of compound **5**.

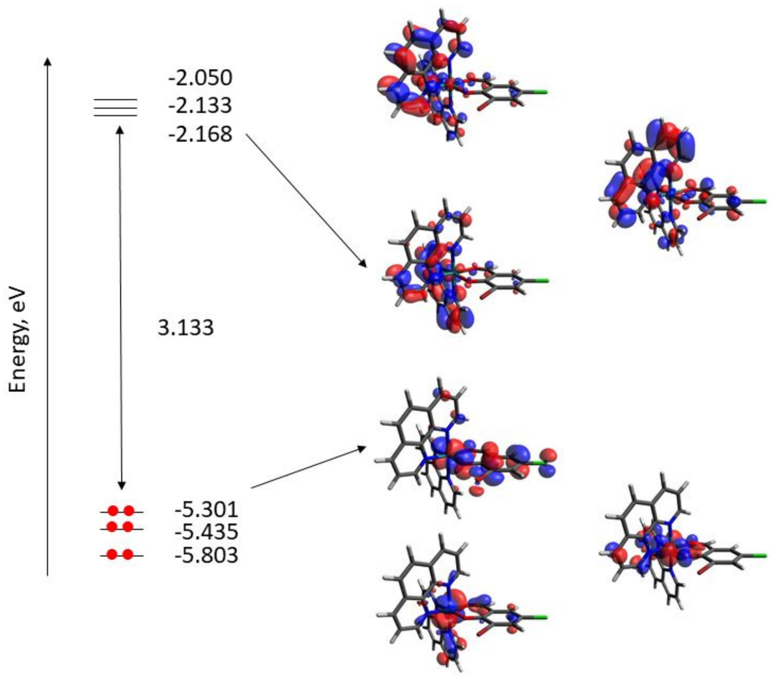

Fig. S19 Energy levels and isosurface contour plots (0.03 a.u.) for cation of compound **6**.

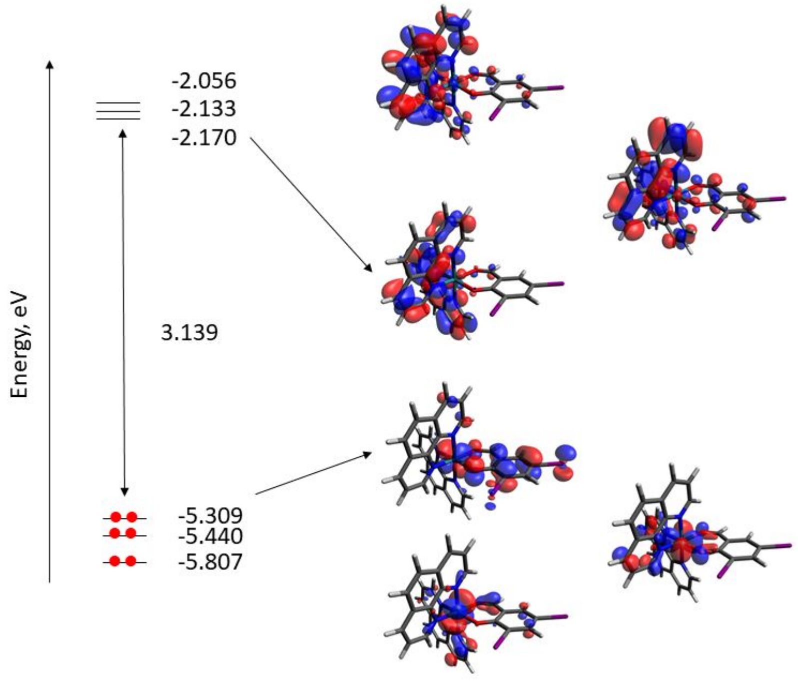

Fig. S20 Energy levels and isosurface contour plots (0.03 a.u.) for cation of compound **7**.

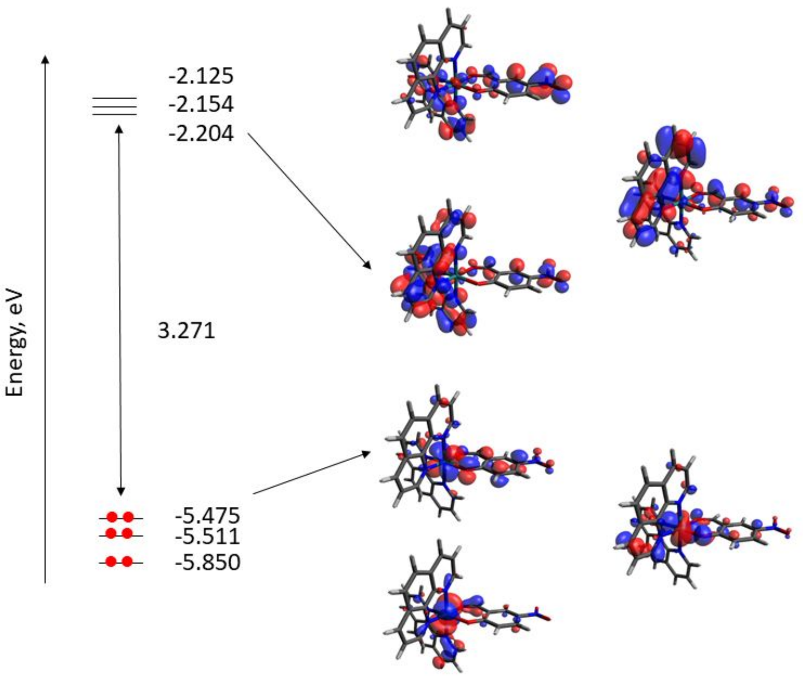

Fig. S21 Energy levels and isosurface contour plots (0.03 a.u.) for cation of compound **8**.

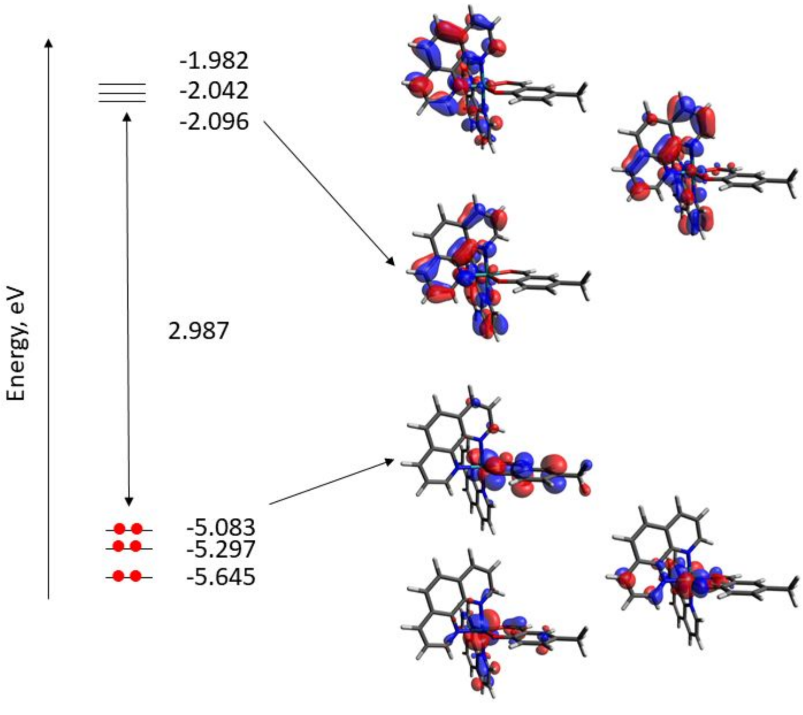

Fig. S22 Energy levels and isosurface contour plots (0.03 a.u.) for cation of compound **9**.

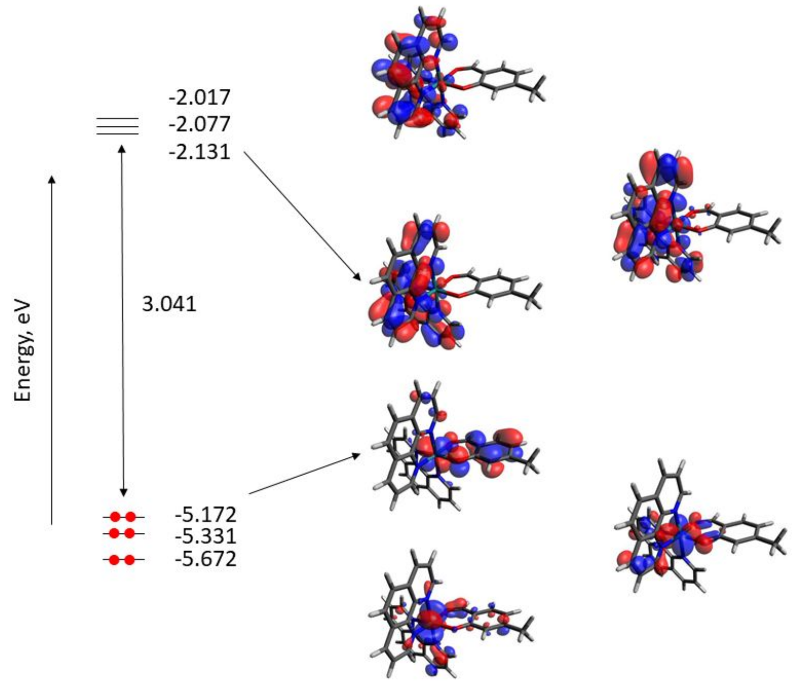

Fig. S23 Energy levels and isosurface contour plots (0.03 a.u.) for cation of compound **10**.

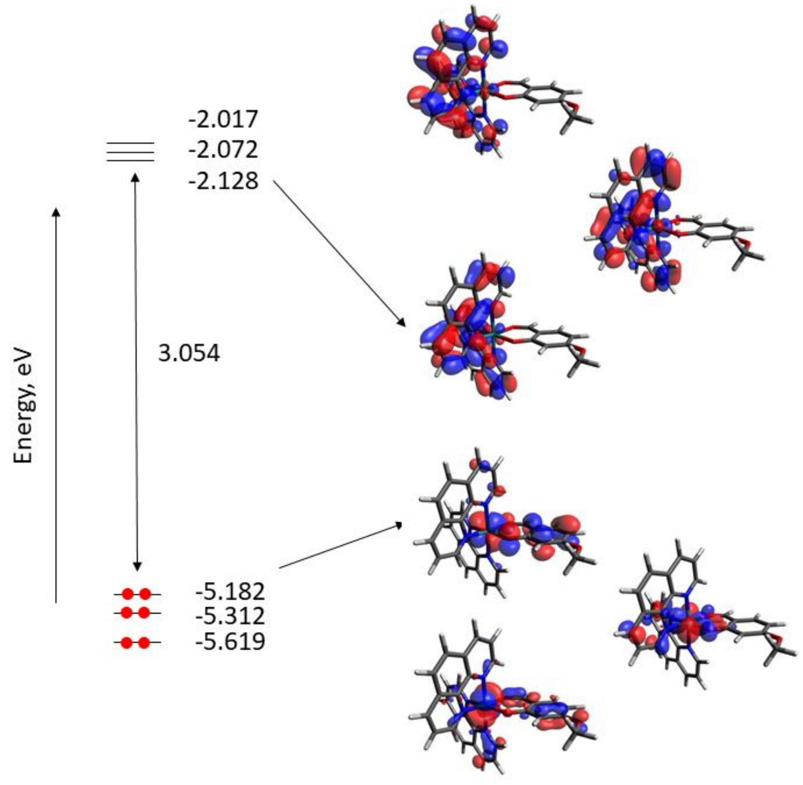

Fig. S24 Energy levels and isosurface contour plots (0.03 a.u.) for cation of compound **11**.

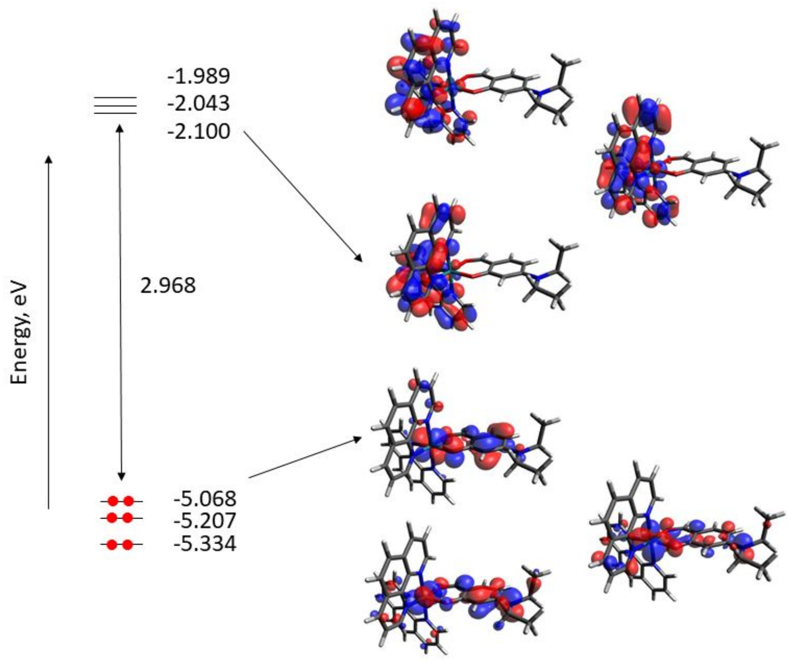

Fig. S25 Energy levels and isosurface contour plots (0.03 a.u.) for cation of compound **12**.

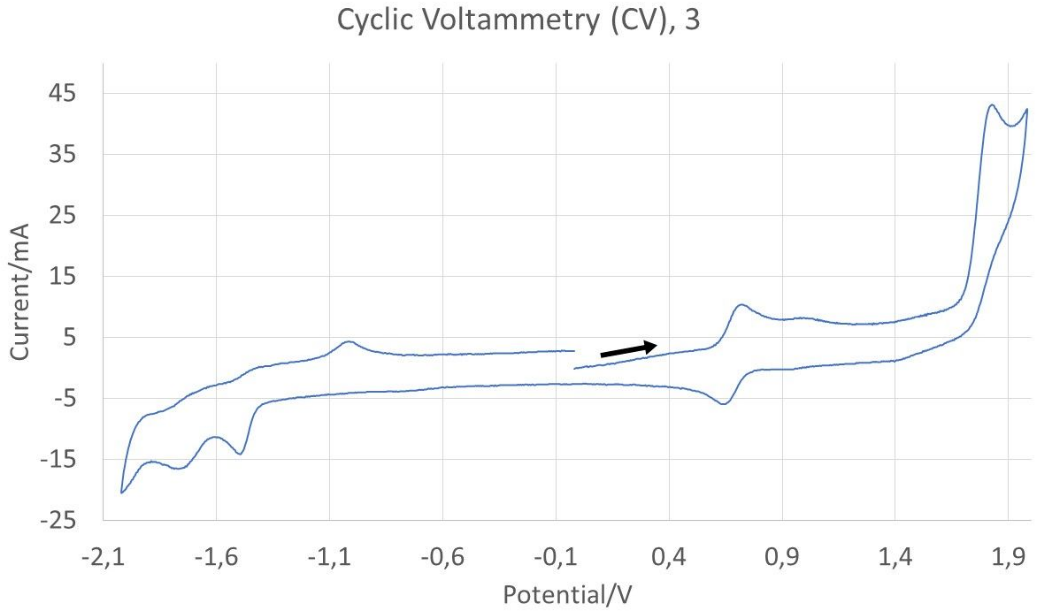

Fig. S26 Cyclic voltammogram of compound **3** in acetonitrile solution (5×10^{-4} M) recorded with scan rate of 0.10 $V{\cdot}s^{-1}$. The arrow indicates the starting point and the sense of the scan.

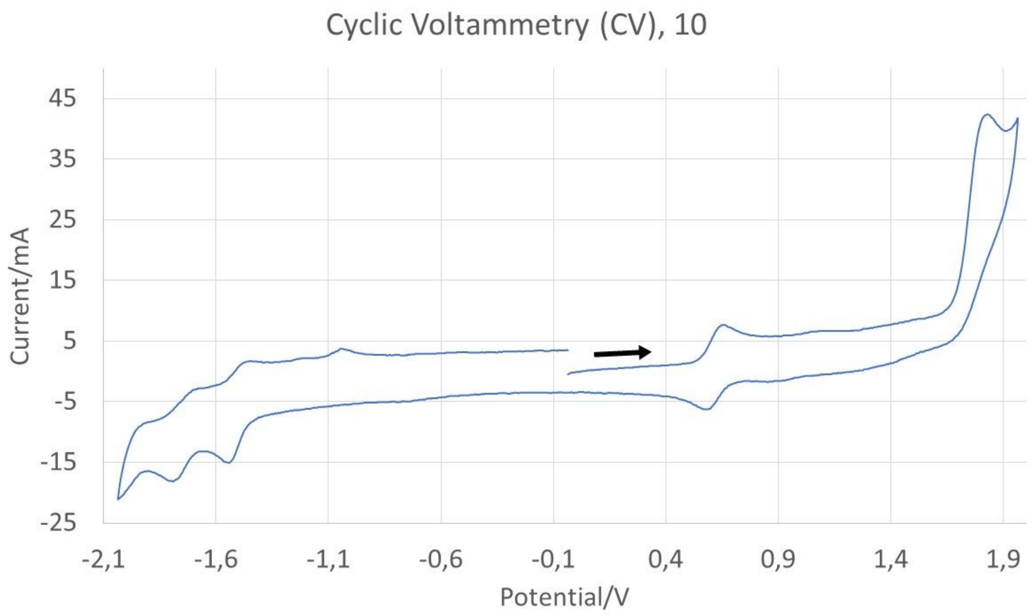

Fig. S27 Cyclic voltammogram of compound **10** in acetonitrile solution (5 × 10^{-4} M) recorded with scan rate of 0.10 V·s^{-1}. The arrow indicates the starting point and the sense of the scan.

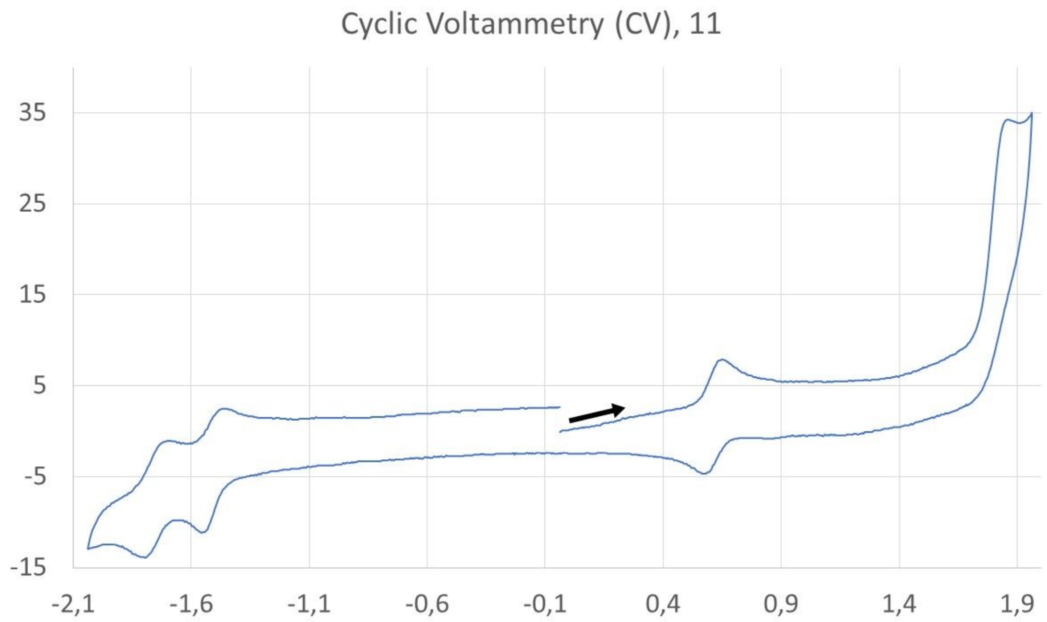

Fig. S28 Cyclic voltammogram of compound **11** in acetonitrile solution (5 × 10^{-4} M) recorded with scan rate of 0.10 V·s^{-1}. The arrow indicates the starting point and the sense of the scan.

Table S1. Participation of the atomic orbitals (grouped by metal and ligands) in the molecular orbitals.

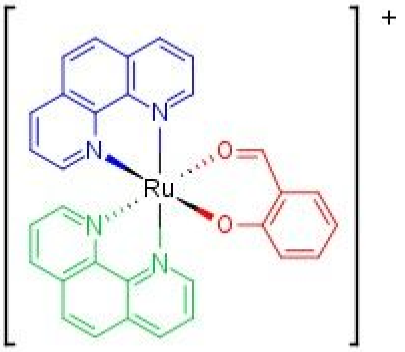

L1, Blue
L2, Green
L3, Red

	H-4	H-3	H-2	H-1	HOMO	LUMO	L+1	L+2	L+3	L+4
**1**										
Ru	3,80	38,43	77,27	73,75	50,79	4,82	5,90	3,04	0,55	3,95
L1	23,47	8,59	5,76	8,30	5,14	37,11	55,24	49,04	47,53	7,05
L2	71,37	4,00	9,73	7,05	5,44	57,20	35,36	46,51	51,43	5,11
L3	1,35	48,99	7,24	10,90	38,63	0,86	3,50	1,41	0,50	83,89
**2**										
Ru	4,05	38,31	77,53	73,98	47,30	4,74	5,42	3,08	0,58	4,31
L1	62,92	7,53	5,83	8,24	4,80	38,30	54,41	56,30	36,37	9,89
L2	31,79	3,74	9,80	7,13	5,03	55,89	33,52	38,32	62,03	5,85
L3	1,24	50,43	6,84	10,65	42,87	1,08	6,65	2,31	1,02	79,95
**3**										
Ru	4,15	37,44	77,51	74,24	46,70	4,68	5,38	3,11	0,60	4,35
L1	78,62	7,11	5,85	8,04	4,95	35,30	57,06	52,36	40,09	10,50
L2	15,52	3,60	9,82	7,29	4,83	58,69	30,67	42,14	58,04	6,05
L3	1,70	51,86	6,82	10,42	43,52	1,33	6,89	2,39	1,28	79,10
**4**										
Ru	3,79	36,76	77,23	73,31	46,89	4,50	4,45	3,80	0,90	4,54
L1	50,07	7,48	5,63	8,32	4,56	29,20	60,17	55,00	26,41	22,61
L2	44,27	3,44	10,04	7,13	5,25	63,14	20,03	36,14	68,57	7,18
L3	1,88	52,32	7,10	11,24	43,29	3,17	15,35	5,07	4,12	65,66
**5**										
Ru	5,25	38,62	77,08	73,49	43,24	4,98	2,16	5,20	1,91	3,96
L1	89,42	7,42	5,47	7,78	4,84	7,56	46,76	41,42	74,42	24,05
L2	1,85	3,32	9,86	7,45	4,60	83,23	45,36	51,49	9,20	4,87
L3	3,48	50,64	7,59	11,28	47,32	4,23	5,72	1,89	14,47	67,13
**6**										
Ru	3,23	36,30	77,08	73,48	46,31	4,41	4,44	4,04	1,14	4,20
L1	51,89	7,37	5,51	7,95	4,89	23,47	65,68	54,95	22,40	26,79
L2	40,07	3,32	10,05	7,39	4,91	67,08	16,22	34,59	70,46	6,70

L3	4,81	53,02	7,35	11,18	43,89	5,04	13,67	6,41	6,00	62,31
**7**										
Ru	1,14	36,14	76,79	45,15	4,46	4,59	3,77	1,08	4,15	1,14
L1	0,57	6,51	5,43	4,81	30,77	59,93	59,55	18,96	24,36	0,57
L2	1,86	2,85	10,08	4,57	61,26	22,36	29,82	75,16	6,64	1,86
L3	96,43	54,50	7,70	45,47	3,51	13,12	6,87	4,80	64,85	96,43
**8**										
Ru	5,45	22,22	77,68	69,75	65,97	2,91	4,40	4,25	2,52	0,19
L1	7,00	10,84	5,83	9,39	4,98	28,48	61,24	6,29	45,52	43,32
L2	86,23	3,51	10,09	5,69	7,05	48,65	8,59	35,29	46,25	50,37
L3	1,32	63,43	6,40	15,17	22,00	19,95	25,78	54,18	5,72	6,12
**9**										
Ru	4,06	43,91	77,10	73,95	45,01	4,83	6,03	2,95	0,56	3,95
L1	3,01	7,99	5,84	8,13	4,76	41,57	51,45	55,07	40,96	6,77
L2	90,76	4,10	9,65	7,13	5,06	52,84	39,11	40,56	58,16	5,12
L3	2,17	44,01	7,40	10,80	45,16	0,76	3,42	1,42	0,32	84,17
**10**										
Ru	3,53	39,11	76,31	73,77	50,16	4,88	6,04	3,09	0,55	3,65
L1	34,23	8,31	5,80	8,23	5,15	37,96	54,05	48,27	49,40	6,32
L2	45,96	4,14	9,54	7,04	5,40	56,38	37,22	47,36	49,73	4,80
L3	16,28	48,44	8,35	10,97	39,30	0,77	2,70	1,28	0,33	85,23
**11**										
Ru	8,41	35,41	72,25	73,45	52,48	4,93	6,16	3,20	0,57	3,26
L1	4,67	7,01	5,81	8,25	5,25	41,91	50,49	53,19	44,51	5,75
L2	3,03	4,59	8,97	6,87	5,57	52,49	41,37	42,39	54,71	4,53
L3	83,89	52,99	12,97	11,43	36,70	0,67	1,97	1,22	0,21	86,46
**12**										
Ru	38,73	51,30	44,02	62,30	49,53	5,03	6,05	3,64	0,67	2,89
L1	8,66	3,83	4,54	7,16	5,27	38,95	51,82	47,56	51,88	5,35
L2	3,82	7,20	6,49	5,09	5,61	55,32	40,53	47,55	47,25	4,20
L3	48,79	37,67	44,94	25,45	39,60	0,71	1,60	1,25	0,21	87,55

Table S2. Selected singlet and triplet excited states calculated at the TDDFT B3LYP/(def2-SVP + LANL2DZ) level for complex **3**$^+$ in DMSO solution[a]

Comp.	State	Energy(eV)	λ(nm)	f.osc.	Monoexcitations	Nature	Description
**3**	S_1	2.198	564.1	0.0032	HOMO → LUMO (18)	d_{π}(Ru) + π_{sal} → π^*_{phen}	^{1}MLCT/^{1}LLCT
					HOMO → LUMO+1 (58)	d_{π}(Ru) + π_{sal} → π^*_{phen}	^{1}MLCT/^{1}LLCT
	S_2	2.221	558.3	0.0010	HOMO → LUMO (65)	d_{π}(Ru) + π_{sal} → π^*_{phen}	^{1}MLCT/^{1}LLCT

	S_3	2.261	548.3	0.0233	HOMO-1 → LUMO (68)<br>HOMO-1 → LUMO+1 (15)	d_{π}(Ru) → π^*_{phen}<br>d_{π}(Ru) → π^*_{phen}	^{1}MLCT<br>^{1}MLCT
	S_4	2.344	528.9	0.0090	HOMO-1 → LUMO+1 (49)<br>HOMO → LUMO+2 (27)	d_{π}(Ru) → π^*_{phen}<br>d_{π}(Ru) + π_{sal} → π^*_{phen}	^{1}MLCT<br>^{1}MLCT/^{1}LLCT
	S_5	2.348	528.0	0.0146	HOMO → LUMO+1 (19)<br>HOMO → LUMO+2 (56)<br>HOMO → LUMO+3 (16)	d_{π}(Ru) + π_{sal} → π^*_{phen}<br>d_{π}(Ru) + π_{sal} → π^*_{phen}<br>d_{π}(Ru) + π_{sal} → π^*_{phen}	^{1}MLCT/^{1}LLCT<br>^{1}MLCT/^{1}LLCT<br>^{1}MLCT/^{1}LLCT
	S_{11}	2.720	455.8	0.1146	HOMO-2 → LUMO (47)<br>HOMO-1 → LUMO+3 (15)	d_{π}(Ru) → π^*_{phen}<br>d_{π}(Ru) → π^*_{phen}	^{1}MLCT<br>^{1}MLCT
	S_{13}	2.908	426.4	0.1568	HOMO-2 → LUMO (16)<br>HOMO-2 → LUMO+1 (22)<br>HOMO-1 → LUMO+2 (17)	d_{π}(Ru) → π^*_{phen}<br>d_{π}(Ru) → π^*_{phen}<br>d_{π}(Ru) → π^*_{phen}	^{1}MLCT<br>^{1}MLCT<br>^{1}MLCT
	T_1	1.857	667.8	----	HOMO → LUMO (33)<br>HOMO → LUMO+1 (29)<br>HOMO → LUMO+2 (22)	d_{π}(Ru) + π_{sal} → π^*_{phen}<br>d_{π}(Ru) + π_{sal} → π^*_{phen}<br>d_{π}(Ru) + π_{sal} → π^*_{phen}	^{3}MLCT/^{3}LLCT<br>^{3}MLCT/^{3}LLCT<br>^{3}MLCT/^{3}LLCT
	T_2	1.991	622.8	----	HOMO → LUMO+1 (41)	d_{π}(Ru) + π_{sal} → π^*_{phen}	^{3}MLCT/^{3}LLCT
	T_3	2.003	619.1	----	HOMO → LUMO+1 (18)<br>HOMO → LUMO+4 (71)	d_{π}(Ru) + π_{sal} → π^*_{phen}<br>d_{π}(Ru) + π_{sal} → π^*_{sal}	^{3}MLCT/^{3}LLCT<br>^{3}MLCT/^{3}LC

[a]Vertical excitation energies (*E*), dominant monoexcitations with contributions (within parentheses) of >15%, the nature of the electronic transition, and the description of the excited state are summarized.

Table S3. Selected singlet and triplet excited states calculated at the TDDFT B3LYP/(def2-SVP + LANL2DZ) level for complex **10**$^{+}$ in DMSO solution[a]

Comp.	State	Energy(eV)	λ(nm)	f.osc.	Monoexcitations	Nature	Description
**10**	S_1	2.162	573.6	0.0021	HOMO → LUMO (84)	$d_{\pi}(Ru) + \pi_{sal} \rightarrow \pi^*_{phen}$	^{1}MLCT/^{1}LLCT
	S_2	2.186	567.1	0.0034	HOMO → LUMO+1 (68)	$d_{\pi}(Ru) + \pi_{sal} \rightarrow \pi^*_{phen}$	^{1}MLCT/^{1}LLCT
	S_3	2.200	563.5	0.020	HOMO-1 → LUMO (69)<br>HOMO-1 → LUMO+1 (20)	$d_{\pi}(Ru) \rightarrow \pi^*_{phen}$<br>$d_{\pi}(Ru) \rightarrow \pi^*_{phen}$	^{1}MLCT<br>^{1}MLCT
	S_4	2.251	550.7	0.0117	HOMO → LUMO+1 (16)<br>HOMO → LUMO+2 (45)<br>HOMO → LUMO+3 (25)	$d_{\pi}(Ru) + \pi_{sal} \rightarrow \pi^*_{phen}$<br>$d_{\pi}(Ru) + \pi_{sal} \rightarrow \pi^*_{phen}$<br>$d_{\pi}(Ru) + \pi_{sal} \rightarrow \pi^*_{phen}$	^{1}MLCT/^{1}LLCT<br>^{1}MLCT/^{1}LLCT<br>^{1}MLCT/^{1}LLCT
	S_5	2.281	543.5	0.0070	HOMO-1 → LUMO+1 (39)<br>HOMO → LUMO+2 (39)	$d_{\pi}(Ru) \rightarrow \pi^*_{phen}$<br>$d_{\pi}(Ru) + \pi_{sal} \rightarrow \pi^*_{phen}$	^{1}MLCT<br>^{1}MLCT/^{1}LLCT
	S_9	2.673	463.9	0.1278	HOMO-2 → LUMO (45)<br>HOMO-1 → LUMO+3 (18)	$d_{\pi}(Ru) \rightarrow \pi^*_{phen}$<br>$d_{\pi}(Ru) \rightarrow \pi^*_{phen}$	^{1}MLCT<br>^{1}MLCT
	S_{13}	2.875	431.3	0.0792	HOMO-2 → LUMO+3 (49)	$d_{\pi}(Ru) \rightarrow \pi^*_{phen}$	^{1}MLCT
	S_{14}	2.920	424.6	0.0951	HOMO-2 → LUMO+1 (27)<br>HOMO-2 → LUMO+3 (41))	$d_{\pi}(Ru) \rightarrow \pi^*_{phen}$<br>$d_{\pi}(Ru) \rightarrow \pi^*_{phen}$	^{1}MLCT<br>^{1}MLCT
	T_1	1.863	665.4	----	HOMO-1 → LUMO (50)<br>HOMO → LUMO (22)<br>HOMO → LUMO+1 (22)	$d_{\pi}(Ru) \rightarrow \pi^*_{phen}$<br>$d_{\pi}(Ru) + \pi_{sal} \rightarrow \pi^*_{phen}$<br>$d_{\pi}(Ru) + \pi_{sal} \rightarrow \pi^*_{phen}$	^{3}MLCT<br>^{3}MLCT/^{3}LLCT<br>^{3}MLCT/^{3}LLCT
	T_2	1.886	657.3	----	HOMO → LUMO+1 (64)	$d_{\pi}(Ru) + \pi_{sal} \rightarrow \pi^*_{phen}$	^{3}MLCT/^{3}LLCT
	T_3	1.967	630.3	----	HOMO-1 → LUMO+1 (43)<br>HOMO-1 → LUMO+2 (21)<br>HOMO → LUMO (32)	$d_{\pi}(Ru) \rightarrow \pi^*_{phen}$<br>$d_{\pi}(Ru) \rightarrow \pi^*_{phen}$<br>$d_{\pi}(Ru) + \pi_{sal} \rightarrow \pi^*_{phen}$	^{3}MLCT<br>^{3}MLCT<br>^{3}MLCT/^{3}LLCT

[a]Vertical excitation energies (*E*), dominant monoexcitations with contributions (within parentheses) of >15%, the nature of the electronic transition, and the description of the excited state are summarized.

Table S4. Lowest singlet and triplet excited states calculated at the TDDFT B3LYP/(def2-SVP + LANL2DZ) level for complex **11⁺** in DMSO solution[a]

Complex	State	Energy(eV)	λ(nm)	f.osc.	Monoexcitations	Nature	Description
**11**	S_1	2.064	600.8	0.0040	HOMO → LUMO (73)<br>HOMO → LUMO+1 (18)	$d_\pi(Ru) + \pi_{sal} \rightarrow \pi^*_{phen}$<br>$d_\pi(Ru) + \pi_{sal} \rightarrow \pi^*_{phen}$	^{1}MLCT/^{1}LLCT<br>^{1}MLCT/^{1}LLCT
	S_2	2.114	586.5	0.0012	HOMO-1 → LUMO (23)<br>HOMO → LUMO+1 (50)	$d_\pi(Ru) \rightarrow \pi^*_{phen}$<br>$d_\pi(Ru) + \pi_{sal} \rightarrow \pi^*_{phen}$	^{1}MLCT<br>^{1}MLCT/^{1}LLCT
	S_3	2.180	568.8	0.0163	HOMO-1 → LUMO (59)<br>HOMO-1 → LUMO+1 (26)	$d_\pi(Ru) \rightarrow \pi^*_{phen}$<br>$d_\pi(Ru) \rightarrow \pi^*_{phen}$	^{1}MLCT<br>^{1}MLCT
	S_4	2.251	550.7	0.0102	HOMO → LUMO+1 (16)<br>HOMO → LUMO+2 (58)<br>HOMO → LUMO+3 (18)	$d_\pi(Ru) + \pi_{sal} \rightarrow \pi^*_{phen}$<br>$d_\pi(Ru) + \pi_{sal} \rightarrow \pi^*_{phen}$<br>$d_\pi(Ru) + \pi_{sal} \rightarrow \pi^*_{phen}$	^{1}MLCT/^{1}LLCT<br>^{1}MLCT/^{1}LLCT<br>^{1}MLCT/^{1}LLCT
	S_5	2.260	548.6	0.0102	HOMO-1 → LUMO+1 (41)<br>HOMO → LUMO+2 (26)	$d_\pi(Ru) \rightarrow \pi^*_{phen}$<br>$d_\pi(Ru) + \pi_{sal} \rightarrow \pi^*_{phen}$	^{1}MLCT<br>^{1}MLCT/^{1}LLCT
	S_9	2.642	469.4	0.1525	HOMO-2 → LUMO (51)<br>HOMO-1 → LUMO+2 (23)	$d_\pi(Ru) \rightarrow \pi^*_{phen}$<br>$d_\pi(Ru) \rightarrow \pi^*_{phen}$	^{1}MLCT<br>^{1}MLCT
	S_{12}	2.849	435.2	0.0669	HOMO → LUMO+4 (78)	$d_\pi(Ru) + \pi_{sal} \rightarrow \pi^*_{sal}$	^{1}MLCT/^{1}LC
	S_{14}	2.994	414.2	0.0807	HOMO-2 → LUMO+1 (29)<br>HOMO → LUMO+4 (17)	$d_\pi(Ru) \rightarrow \pi^*_{phen}$<br>$d_\pi(Ru) + \pi_{sal} \rightarrow \pi^*_{saln}$	^{1}MLCT<br>^{1}MLCT/^{1}LC
	T_1	1.761	703.9	----	HOMO-1 → LUMO (38)<br>HOMO → LUMO (26)<br>HOMO → LUMO+1 (27)	$d_\pi(Ru) \rightarrow \pi^*_{phen}$<br>$d_\pi(Ru) + \pi_{sal} \rightarrow \pi^*_{phen}$<br>$d_\pi(Ru) + \pi_{sal} \rightarrow \pi^*_{phen}$	^{3}MLCT<br>^{3}MLCT/^{3}LLCT<br>^{3}MLCT/^{3}LLCT
	T_2	1.821	681.0	----	HOMO → LUMO (17)<br>HOMO → LUMO+1 (61)	$d_\pi(Ru) + \pi_{sal} \rightarrow \pi^*_{phen}$<br>$d_\pi(Ru) + \pi_{sal} \rightarrow \pi^*_{phen}$	^{3}MLCT/^{3}LLCT<br>^{3}MLCT/^{3}LLCT
	T_3	1.964	631.3	----	HOMO-1 → LUMO+1 (51)<br>HOMO-1 → LUMO+2 (26)<br>HOMO → LUMO (22)	$d_\pi(Ru) \rightarrow \pi^*_{phen}$<br>$d_\pi(Ru) \rightarrow \pi^*_{phen}$<br>$d_\pi(Ru) + \pi_{sal} \rightarrow \pi^*_{phen}$	^{3}MLCT<br>^{3}MLCT<br>^{3}MLCT/^{3}LC

[a]Vertical excitation energies (*E*), dominant monoexcitations with contributions (within parentheses) of >15%, the nature of the electronic transition, and the description of the excited state are summarized.
